# Recent Advances in Zinc Oxide Nanostructures with Antimicrobial Activities

**DOI:** 10.3390/ijms21228836

**Published:** 2020-11-22

**Authors:** Yuchao Li, Chengzhu Liao, Sie Chin Tjong

**Affiliations:** 1 Department of Materials Science and Engineering, Liaocheng University, Liaocheng 252000, China; liyuchao@lcu.edu.cn; 2 Department of Materials Science and Engineering, Southern University of Science and Technology, Shenzhen 518055, China; 3 Department of Physics, City University of Hong Kong, Tat Chee Avenue, Kowloon, Hong Kong, China

**Keywords:** semiconducting oxide, synthesis, heterostructure, antimicrobial activity, photocatalytic activity, free radicals, zinc ion, contact killing, phytocompounds, hemolysis

## Abstract

This article reviews the recent developments in the synthesis, antibacterial activity, and visible-light photocatalytic bacterial inactivation of nano-zinc oxide. Polycrystalline wurtzite ZnO nanostructures with a hexagonal lattice having different shapes can be synthesized by means of vapor-, liquid-, and solid-phase processing techniques. Among these, ZnO hierarchical nanostructures prepared from the liquid phase route are commonly used for antimicrobial activity. In particular, plant extract-mediated biosynthesis is a single step process for preparing nano-ZnO without using surfactants and toxic chemicals. The phytochemical molecules of natural plant extracts are attractive agents for reducing and stabilizing zinc ions of zinc salt precursors to form green ZnO nanostructures. The peel extracts of certain citrus fruits like grapefruits, lemons and oranges, acting as excellent chelating agents for zinc ions. Furthermore, phytochemicals of the plant extracts capped on ZnO nanomaterials are very effective for killing various bacterial strains, leading to low minimum inhibitory concentration (MIC) values. Bioactive phytocompounds from green ZnO also inhibit hemolysis of *Staphylococcus aureus* infected red blood cells and inflammatory activity of mammalian immune system. In general, three mechanisms have been adopted to explain bactericidal activity of ZnO nanomaterials, including direct contact killing, reactive oxygen species (ROS) production, and released zinc ion inactivation. These toxic effects lead to the destruction of bacterial membrane, denaturation of enzyme, inhibition of cellular respiration and deoxyribonucleic acid replication, causing leakage of the cytoplasmic content and eventual cell death. Meanwhile, antimicrobial activity of doped and modified ZnO nanomaterials under visible light can be attributed to photogeneration of ROS on their surfaces. Thus particular attention is paid to the design and synthesis of visible light-activated ZnO photocatalysts with antibacterial properties

## 1. Introduction

Poor hygiene and ineffective hospital infection control practice contribute to the transmission of nosocomial pathogens. Antibiotics are the drugs commonly used for combating microorganisms. However, the overuse of antibiotics in humans and animals has led to the emergence of multidrug resistant (MDR) bacteria [1,2,3,4]. *Staphylococcus aureus* and *Staphylococcus epidermidis* are opportunistic pathogens that can cause surgical wound infections, bloodstream infections and infections associated with indwelling medical devices. They are capable of developing resistance to multiple antibiotics. Infections caused by MDR bacteria such as methicillin resistant *Staphylococcus aureus* (MRSA) and methicillin-resistant *Staphylococcus epidermidis* (MRSE) are difficult to treat, leading to high morbidity and mortality in patients [5,6,7]. Thus the spread of MDR bacteria poses a serious threat to public health globally. This motivates the researchers to develop various novel nanomaterials as alternatives to antibiotics for the treatment of MDR bacterial infections [8,9].

**Figure 1:**
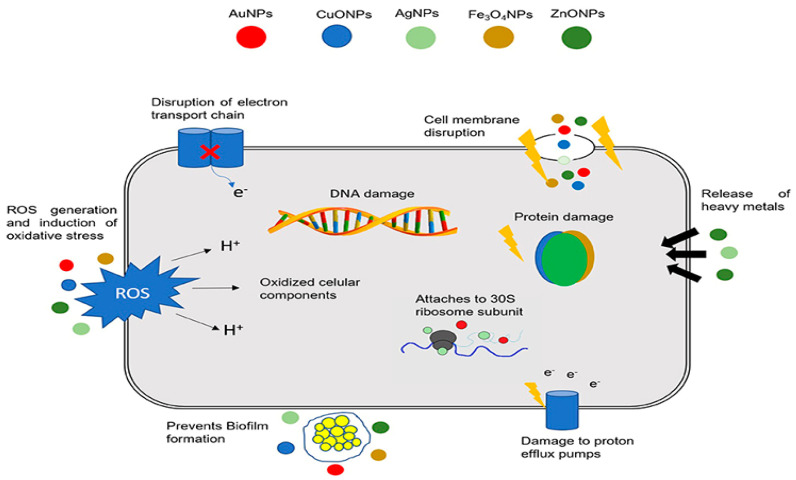
Antibacterial mechanisms of different nanoparticles (NPs) in fighting MDR bacteria. AuNPs: gold NPs, CuONPs: copper oxide NPs; AgNPs: silver NPs; Fe_3_O_4_NPs: iron oxide NPs, and ZnONPs: zinc oxide NPs. Reproduced from [16] under the terms of the Creative Commons Attribution License (CC BY).

Nanomaterials with enhanced chemical, physical and mechanical characteristics than their bulk micromaterials have received considerable attention in materials science community recently [10,11,12,13,14,15,16,17]. Metal and metal oxide nanoparticles including silver, zinc, copper, zinc oxide, titanium dioxide, copper oxide, and iron oxide are known to exhibit antimicrobial properties against various bacterial strains [13,16,17,18,19,20,21,22,23,24,25,26,27,28,29,30,31]. Several mechanisms have been proposed for bactericidal activities of those nanoparticles (NPs) (Figure 1). These include: (a) the disruption of bacterial cell membrane and electron transport chain; (b) the generation of reactive oxygen species (ROS) and induction of oxidative stress, resulting from the interaction of NPs with bacterial cell membrane, and cellular components such as deoxyribonucleic acid (DNA) and protein; (c) released metal ions from metal or metal oxide NPs in intracellular environment. Those ions can react with the functional groups of nucleic acid and protein such as –SH, –NH and –COOH [14,16,25]. As an example, silver nanoparticles (AgNPs) can interact with bacteria by releasing zero-valent metal (Ag^0^) or Ag^+^ ions, so creating ROS and oxidative stress. In this perspective, AgNPs are widely used in various commercial and healthcare products such as textiles, cosmetics, coatings, food packaging and wound dressings [14,32,33]. However, AgNPs are toxic, and would reduce the viability of several types of human cells in size- and concentration-dependent manners [14,34].

**Figure 2:**
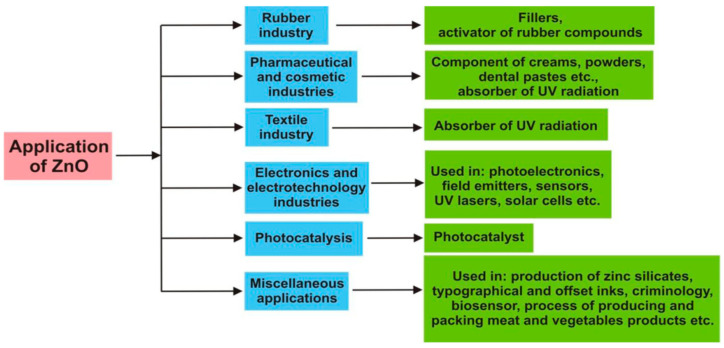
Schematic representation showing versatile applications of ZnO nanoparticles in chemical, electronic, textile, pharmaceutical and cosmetic industries. Reproduced from [35] the terms of the Creative Commons Attribution License (CC BY).

Zinc oxide (ZnO) with versatile properties is currently used for a wide range of chemical, electronic, textile, pharmaceutical and medical applications, including fillers of rubber composites, sunscreens, coatings, optoelectronic devices, sensors, photocatalysts and antimicrobial wound dressings. In rubber industry, ZnO is often employed as an additive for the sulfur vulcanization of rubbers, thereby enhancing the efficiency of the cross-linking system (Figure 2) [21,35]. ZnO is an n-type semiconductor due to the presence of zinc interstitials and oxygen vacancies in its crystalline lattice. It has a large exciton binding energy of 60 meV, and a wide bandgap energy of 3.37 eV [36]. Moreover, ZnO exhibits a high dielectric permittivity (κ = 8.66), thus making it an attractive material for forming gate dielectric in semiconductor devices [37]. Generally, hafnium oxide (HfO_2_) with a larger bandgap (5.3–5.7 eV) is commonly used as a high-κ gate insulating material in semiconductors [38]. So HfO_2_ and ZnO dielectrics can effectively replace conventional thermally grown SiO_2_ gate oxide [37]. ZnO with a wide bandgap absorb ultraviolet (UV) light effectively, giving rise to antibacterial and UV-protection properties. In this respect, ZnO nanostructures show great potential for use in photodetectors, laser diodes, sensors, and solar cells [39,40,41]. Moreover, ZnO NPs can be incorporated into polymers, textile or cotton fibers, thereby producing UV-absorbing packaging films and fabrics for food and medical textile applications [42,43,44,45,46]. By utilizing polymers with several beneficial characteristics such as ease of processing, good moldability, and mechanical properties [47,48,49,50,51,52,53,54], materials scientists are capable of developing functional nanocomposites with UV-shielding properties. More importantly, nano-ZnO exhibits selective toxicity to bacteria, rendering its great potential for antibacterial applications in many fields such as water disinfection, food preservation and medical wound dressings [27,43,44,45,46,55].

ZnO also exhibits additional attractive properties such as low cost, high photochemical stability and photocatalytic activity [56]. In general, irradiation of a photocatalytic semiconductor with photons of equal or higher energy than its bandgap, electron in the valence band (VB) jumps across the large bandgap into the conduction band (CB), creating a positively charged hole in the VB simultaneously. The photoinduced electron (*e*^−^)-hole (*h^+^*) pair then migrates to the semiconductor surface that provides reactive sites for these charge carriers. As such, *e*^−^ reacts with adsorbed oxygen molecules to form superoxide anion (•O_2_^−^), while *h^+^* reacts with water/hydroxyl molecules to yield hydroxyl radical (•OH) and H^+^, as well as hydrogen peroxide (H_2_O_2_). The •O_2_^−^, •OH and H_2_O_2_ are generally referred to as “reactive oxygen species”. ZnO has a wide bandgap of 3.37 eV, resulting in a sharp UV emission in luminescence spectrum [36,57]. By interacting ZnO with bacterial cells under UV irradiation, *e*^−^-*h^+^* pairs can be created readily for the ROS production. Those radical species are very effective in disrupting bacterial respiratory chain, DNA and protein functions, leading to cell death [58]. However, UV radiation accounts for less than 5% while visible light makes up 45% of the total solar spectrum [59]. Therefore it is necessary to extend the optical response of ZnO from UV into the visible light region for practical purposes. This can be achieved by doping nano-ZnO with metals and non-metals, modifying with carbon nanomaterials, and coupling with other oxide semiconductors to form heterostructures [60,61,62,63,64,65,66]. In a previous article, we summarized and discussed the toxic effects of nano-ZnO toward mammalian (eukaryotic) cells [67]. This article provides an updated comprehensive review on the synthesis and antibacterial activity of ZnO hierarchical nanostructures. Particular attention is paid to the the latest developments in the design and synthesis of ZnO heterostructures with visible-light photocatalytic bactericidal activity via metal and non-metal doping, carbon nanomaterial modification, and semiconductor heterojunction formation.

## 2. Photocatalytic Property of Nanostructured ZnO

The creation, recombination and separation of photoinduced charge carriers affect photocatalytic performance of nano-ZnO greatly. Moreover, photocatalytic behavior depends greatly on the size, structure, shape, and defects of ZnO nanostructures. Nanosized ZnO with enhanced surface area exhibits improved photocatalytic activity for bacterial inactivation than micro-ZnO. ZnO exhibits three crystalline structures including wurtzite (B4), zinc blende (B3), and rocksalt (B1). Wurtzite is the most common and thermodynamically stable crystal structure. ZnO zinc-blende phase is metastable under ambient conditions. The wurtzite structure of ZnO transforms into cubic rocksalt structure under high pressures ≥6 GPa [68,69,70,71]. Wurtzite ZnO is hexagonal with a space group of P6_3_mc having lattice parameters a = 0.32496 nm and c = 0.52042 nm; the c/a ratio is 1.6018, being close to that of an ideal hexagonal close-packed structure of 1.633 [72]. In the hexagonal unit cell, four oxygen ions are bounded with each tetrahedral Zn ion, and each tetrahedral oxygen ion is coordinated by four Zn ions. So the wurtzite-type ZnO consists of tetrahedrally coordinated O^2-^ and Zn^2+^ ions, which are piled alternatively along the c-axis [73]. The specific distribution of Zn cations and O^2-^ anions leads to the formation of polar Zn-(0001) and O-(0001) planes. The positively charged Zn-terminated (0001) surface is chemically active, while negatively charged O-terminated (0001) is relatively inert. Thus Zn-(0001) surface favors the growth of ZnO nanocrystals along the <0001> direction [74,75], giving rise to the formation of nanoparticles, nanorods and nanoflowers [74,76,77,78,79,80,81,82,83].

The photocatalytic activity of ZnO is limited by several factors including fast recombination of electron–hole pairs under UV irradiation, poor visible-light absorbance, and susceptibility to photocorrosion in aqueous environments. The photogenerated charge carriers can readily recombine either in the bulk or at the surface of ZnO through the dissipation of energy in form of light or heat. Photocorrosion arises from the dissolution of ZnO into Zn^2+^ ions in aqueous solutions under UV irradiation [84,85,86,87,88,89]. As such, oxygen atoms in the ZnO lattice are oxidized by photogenerated holes to release Zn^2+^. In other words, photogenerated holes on the ZnO surface attack the Zn-O bond, leading to the dissociation of Zn^2+^ from the surface [85,86]. So the holes would react with ZnO rather than with adsorbed surface water molecules to form hydroxyl radicals. Photocorrosion reactions of ZnO with photoinduced holes are expressed as follows [86,87,88],

ZnO + 2*h^+^* → Zn^2+^ + 0.5 O_2_ (1)

or

ZnO + H_2_O + 2*h^+^*→ Zn^2+^ + 2OH (2)

In addition, ZnO undergoes dissolution in strong acidic and alkaline solutions, thereby degrading its photocatalytic performance [85]. As such, photocatalytic activity of ZnO is limited in mild pH solutions only. The chemical reactions of ZnO in acidic and alkaline solutions are given by [87,88],

Acidic medium ZnO + 2H^+^ →Zn^2+^ + H_2_O (3)

Alkaline medium ZnO + H_2_O + 2OH^–^ → [Zn(OH)_4_]^2−^ (4)

Because of the above mentioned drawbacks, ZnO nanostructures have not been explored fully as TiO_2_ NPs with good photostability for photocatalytic applications. It remains a big challenge to develop nano-ZnO with excellent photocatalytic efficacy. In this respect, much efforts have been spent by the researchers to improve the photocatalytic performance of nano-ZnO by suppressing electron-hole pair recombination, extending the optical absorption edge to the visible region, and enhancing photocorrosion resistance [87,88,89,90,91,92]. Recombination of electron-hole pairs can be impeded by trapping photogenerated electrons or holes, thereby extending the lifetime of separated charge carriers. The photocatalytic activity of nano-ZnO under visible light can be improved through the induction of oxygen vacancy defects, the doping of metal/non-metal elements, the modification with carbon nanomaterials, and the formation of heterojunctions by coupling with other semiconductor oxides. In general, oxygen vacancies enhance optical absorbance of ZnO in the visible region. Those vacancies produce midgap state near the top of VB of ZnO, acting as a deep donor level [93,94,95,96].

### 2.1. Metal Dopants

Magnesium (alkaline earth metal), transition metals, and noble metals can be utilized to mofify photocatalytic activity of nano-ZnO. Magnesium ions with a smaller ionic radius can replace zinc ions in the ZnO lattice. Magnesium doping generally widens the bandgap of ZnO NPs [97,98,99]; the bandgap values increase with increasing Mg dopant concentrations [99]. As mentioned, ZnO with a wide bangap is ineffective to induce electron-hole pairs under visible light. However, magnesium doping induces many oxygen vacancy defects in ZnO NPs due to the size difference between Mg^2+^ (0.057 nm) and Zn^2+^ ions (0.074 nm) [97,100]. Those oxygen vacancies facilitate the generation of a high level of ROS in MgZnO NPs under dark, daylight and visible light. As a result, MgZnO NPs exhibit good antibacterial activity against *E. coli* with or without visible light/daylight [97].

#### 2.1.1. Transition Metal Doped ZnO

**Figure 3:**
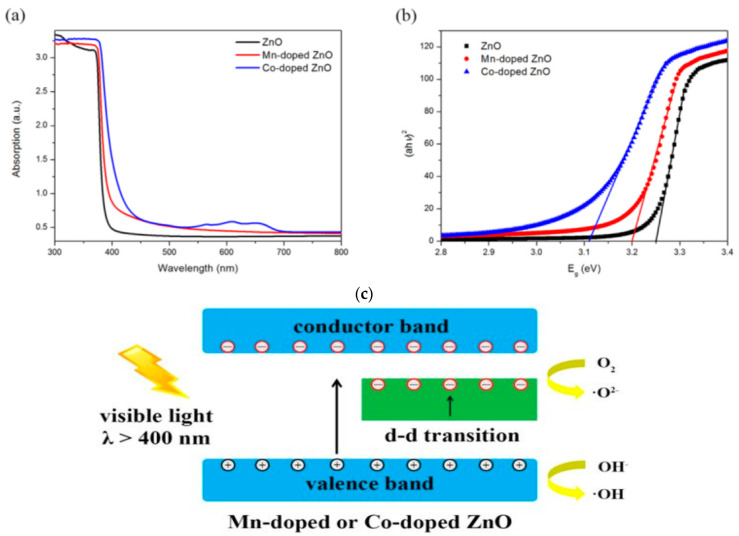
(**a**) UV-visible spectra of pure ZnO, Mn-doped ZnO and Co-doped ZnO nanowires, and (**b**) Tauc plots of (αh*v*)^2^ versus *hv* for ZnO-based nanowires showing the reduction of bandgap energy through transition metal doping. (**c**) The generation of superoxide anion and hydroxyl radicals on ZnO nanowires under visible light due to the creation of midgap states in the bandgap by doping with Mn or Co. Reproduced from [108] under the terms of the Creative Commons Attribution License (CC BY).

**Figure 4:**
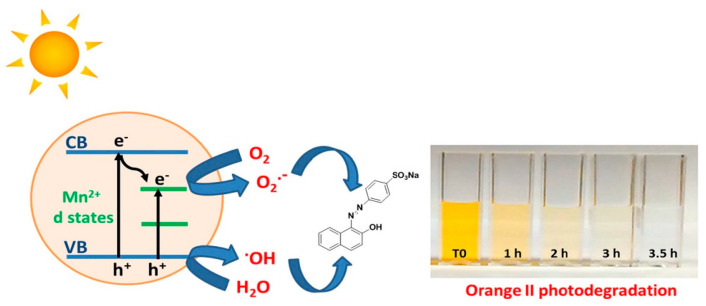
Formation of superoxide anion and hydroxyl radicals on Mn-doped ZnO nanoparticles for degrading Orange II dye under solar light irradiation at different intervals of time. Reproduced from [109] with permission of Elsevier.

The photocatalytic performance of ZnO nanostructures under visible light can be enhanced by doping with transition metals and noble metal NPs. Typical transition metal dopants include copper (Cu), iron (Fe), cobalt (Co), manganese (Mn), nickel (Ni) and chromium (Cr) [100,101,102,103,104,105,106,107,108,109,110,111,112], while noble metal NPs used are gold (Au), and silver (Ag) [113,114,115,116,117,118,119]. The ionic radius of Zn^2+^, Co^2+^, Cu^2+^, and Mn^2+^ is 0.074, 0.072, 0.073, and 0.080 nm, respectively [100,106]. Apparently, Co^2+^ and Cu^2+^ are the best transition metal dopants because of their similar ionic size with Zn^2+^. These cations can readily substitute for Zn^2+^ without causing a large lattice distortion. Figure 3a shows the UV-visible spectra of pure ZnO, Mn-doped ZnO and Co-doped ZnO nanowires. The bandgap energy of these nanowires determined from Tauc plots are shown in Figure 3b [108]. The bandgap of ZnO (3.26 eV) reduces to 3.20 eV and 3.11 eV by doping with Mn^2+^ and Co^2+^, respectively. The bandgap reduction can be attributed to the introduction of midgap states below the CB (Figure 3c). The midgap states serve as effective sites for trapping electrons to retard recombination of electron-hole pairs. A red shift of the optical absorption edge facilitates the formation of ROS on Co- and Mn-doped ZnO under visible light irradiation. Figure 4 shows an example of the formation of oxygen superoxide and hydroxyl radicals on Mn-doped ZnO NPs for degrading Orange II dye upon solar light irradiation [109].

As recognized, two or more types of metallic cations can substitute for Zn^2+^ in the ZnO lattice to further improve the phototocatalytic activity. This is commonly referred to as the ‘co-doping’ approach. Comparing with a single-element doping, enhanced photocatalytic performance of co-doped ZnO arises from the synergistic effect of each ionic dopants in increasing visible light absorption. As such, co-doping is a promising approach for tuning the dopant populations, electronic properties, and magnetic properties of metal oxide semiconductors [120]. Recently, Modwi et al. reported that doping ZnO with 5% Cu alone reduces its bandgap from 3.15 to 2.95 eV. Moreover, co-doping 5%Cu/ZnO NPs with 1% Fe, 1% %Ni, or 1% Ag further decreases the bandgap to 2.90, 2.83 and 2.79 eV, respectively [101].

#### 2.1.2. Noble Metal Doped ZnO

**Figure 5:**
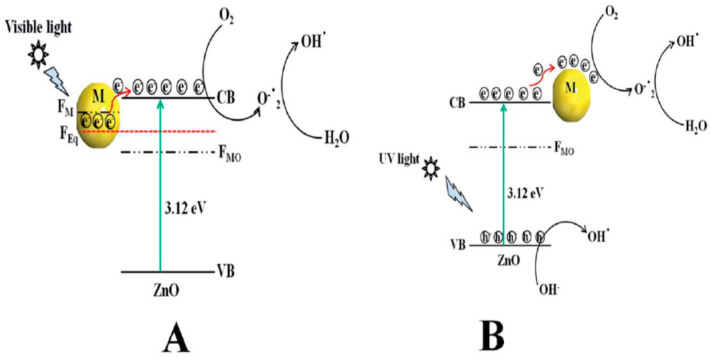
(**A**) Visible light induces localized surface plasmon resonance (LSPR) in AgNPs or AuNPs such that hot electrons are injected into the CB of ZnO for generating superoxide anion and hydroxyl radicals. (**B**) Under UV irradiation, photoexcited electrons are transferred from the CB of ZnO to AgNPs or AuNPs. VB = valence band, CB: conduction band, FMO: Fermi level of metal oxide, FM: Fermi level of metal, FEq: Fermi level of equilibrium, and M: Au and Ag. Reproduced from [127] with permission of the Royal Society of Chemistry.

The photocatalytic efficiency of ZnO under visible light can also be improved by doping with noble metals such as Au, and Ag [113,114,115,116,117,118]. By modifying with noble metals, a Schottky barrier is established at the interface junction that facilitates the electron-hole pair separation, thus retarding the recombination of photogenerated charge carriers [121]. Furthermore, noble metal NPs exhibit localized surface plasmon resonance (LSPR) resulting from a collective oscillation of free electrons in the electromagnetic field of the incident light. After excitation, the electron oscillations lose their collective nature in which LSPR decays into hot electrons and holes via Landau damping at a relatively short time scale [122,123,124]. The frequency range for inducing LSPR effect relies greatly on the chemical nature of noble metal nanoparticles. For instance, AgNPs and AuNPs absorb photons and induce LSPR upon visible light irradiation. In contrast, platinum (Pt) NPs display LSPR in the UV range, so exhibiting a strong absorption at ~380 nm [125]. Therefore, PtNPs induce a blue shift in the optical spectrum of Pt/ZnO nanocomposites [126]. In this respect, plasmonic AgNPs are commonly employed to enhance photocatalytic efficiency of ZnO under visible light [127,128]. As mentioned, a Schottky barrier is established at the metal/ZnO interface when noble metal comes in contact with ZnO. The Fermi energy level (FM) of AgNPs or AuNPs is higher than ZnO Fermi level (FMO), leading to the transfer of electron from the FM of AgNPs or AuNPs to the FMO until the attainment of a new equilibrium Fermi level (FEq) [127]. Under visible light, LSPR absorption causes a rapid heating of noble metal NPs.Thus hot electrons from AgNPs/AuNPs are injected into the CB of ZnO for reacting with adsorbed oxygen molecules to create superoxide anion radical. The superoxide anion then reacts with H_2_O to generate hydroxyl radicals. On the contrary, photoexcited electrons in the CB of ZnO are transferred to AgNPs or AuNPs upon exposure to UV light. This is because CB of ZnO is higher than the newly generated Fermi energy level of AgNPs/ZnO or AuNPs/ZnO. As such, AgNPs or AuNPs act as electron trapping sites, thus enhancing the charge separation efficiency of ZnO (Figure 5A,B) [127].

### 2.2. Non-Metal Dopants

Metal dopants have a certain limitation for achieving high photocatalytic activity under visible-light, especially at high concentrations. This is due to photogenerated charge carriers recombine at trapping defect sites, leading to a reduction in the photocatalytic efficiency. In this respect, non-metal dopants such as carbon, nitrogen, and sulfur can be used to improve visible-light photocatalytic activity of ZnO [129,130,131,132,133,134]. Non-metal dopants introduce an intermediate energy level just above the VB of ZnO. Accordingly, the dopants narrow the bandgap and facilitate the separation of photoexcited charge pairs in ZnO. For example, sulfur dopant can elevate the valence band maximum by hybridizing S-3p orbital with the VB of ZnO, leading to a bandgap reduction of ZnO from 3.22 eV to 2.85 eV [129]. Zhang et al. demonstrated that carbon doping is effective for narrowing bandgap of ZnO nanorods from ~3.19 to ~2.72 eV. Carbon doping induces the formation of oxygen vacancy defects in ZnO, thereby serving as the trapping sites for photogenerated electrons. In this respect, carbon doping and oxygen vacancies increase the separation efficiency of photogenerated electron−hole pairs, thus enhancing the photocatalytic activity of ZnO [130]. Similarly, carbon-doped ZnO nanorods and C-doped ZnO nanoparticles have been reported to have a reduced bandgap energy of 2.69 and 3.08 eV, respectively [131].

#### Metal/Non-Metal Co-Dopants

**Figure 6:**
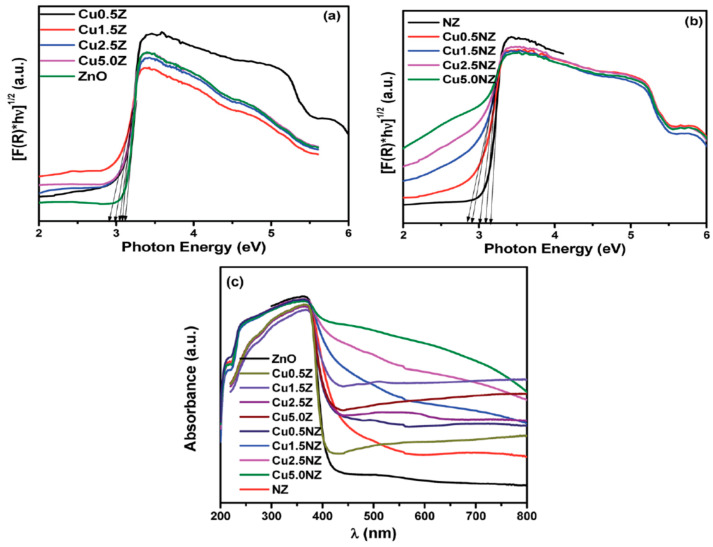
The plots of Kubelka–Munk function vs photon energy for evaluating bandgap energy of (**a**) ZnO and Cu0.5Z, Cu1.5Z, Cu2.5Z and Cu5.0Z, and (**b**) NZ, Cu0.5NZ, Cu1.5NZ, Cu2.5NZ and Cu5.0NZ. (**c**) Optical absorbance of ZnO, Cu0.5Z, Cu1.5Z, Cu2.5Z, Cu5.0Z, NZ, Cu0.5NZ, Cu1.5NZ, Cu2.5NZ and Cu5.0NZ. Reproduced from [135] with permission of the Royal Society of Chemistry.

In a recent study of Gupta et al., N and Cu co-dopants narrow the bandgap of ZnO NPs effectively [135]. Figure 6a shows the Tauc plots of the Kubelka–Munk function vs photon energy for ZnO NPs, and Cu-doped ZnO NPs with 0.5%, 1.5%, 2.5% and 5.0% Cu, denoting as Cu0.5Z, Cu1.5Z, Cu2.5Z and Cu5.0Z, respectively. The bandgap of ZnO NPs is 3.16 eV, while that of the Cu0.5Z, Cu1.5Z, Cu2.5Z and Cu5.0Z is 3.09, 3.00, 2.91 and 2.84 eV, respectively. By doping with nitrogen, the bandgap of N-doped ZnO slightly decreases to 3.11 eV (Figure 6b). Co-doping of ZnO with N and 0.5%, 1.5%, 2.5%, 5.0% Cu (i.e., Cu0.5NZ, Cu1.5NZ, Cu2.5NZ and Cu5.0NZ) leads to a further bandgap reduction having the energy value of 3.07, 2.91, 3.03 and 2.98 eV, respectively. The reduction of band gap is due to the hybridization of N-2p state with O-2p level. This creates a defect energy state just above the VB of ZnO, serving as the site for trapping the holes. Meanwhile, Cu^2+^-doping introduces a sub-energy level below the CB, acting as the site for trapping electrons. These defect states promote photogenerated charge separation, and improve photocatalytic activity in the visible region. Figure 6c reveals that the (N, Cu) co-doped Cu5.0NZ exhibits a higher absorbance than Cu5.0Z in the visible light region. This is due to the synergistic action of both Cu^2+^ and N dopants in Cu5.0NZ during the photoexcitation process [135]. Similarly, synergistic effects of both Mn^2+^ and N dopants also enhance the absorption of ZnO nanofibers in the visible-light region [136].

### 2.3. Carbon Nanomaterials Modified ZnO

#### 2.3.1. Graphene

Graphene exhibits a two-dimensional honeycomb lattice consisting of a single layer of sp^2^ hybridized carbon atoms. It is a building block of carbonaceous materials including zero-dimensional (0D) fullerene, one-dimensionl (1D) carbon nanotube (CNT), and three-dimensional (3D)-graphite [137]. A single graphene layer rolls into a cylindrical shape to form a single-walled carbon nanotube (SWCNT), while multiple graphene layers roll into a multi-walled carbon nanotube (MWCNT). Graphene and CNTs exhibit excessively high elastic modulus (~1 TPa), excellent electrical conductivity, high optical transparency, large surface area, and superior antimicrobial properties [13,137,138,139,140,141,142,143]. Therefore, graphene and its derivatives, as well as CNTs show attractive applications as transparent conducting films in optoelectronic devices, conducting fillers of functional polymer nanocomposites, active photocatalysts for water splitting, and drug delivery vehicles in the pharmaceutical field [50,51,52,53,54,144,145,146,147,148,149,150,151,152,153,154,155,156,157,158,159]. In the case of photocatalysis, graphene and CNTs improve the photocatalytic activity and reduce photocorrosion of ZnO markedly [89,160,161,162].

Large-area graphene layers for optoelectronic applications are generally prepared from chemical vapor deposition (CVD) [160,161]. However, the high cost of fabrication, tedious film transferring procedure, contamination and defect formation in the film during the transfer process, have limited the widespread use of graphene in optoelectronic industry [163,164,165,166]. In this respect, graphene oxide (GO) is considered as an alternative for graphene. It can be synthesized in large quantities by oxidation–exfoliation of graphite flakes in a mixture of concentrated sulfuric acid, sodium nitrate, and potassium permanganate, using a modified Hummers process [167]. The as-synthesized GO contains hydroxyl and epoxide groups on the graphene basal plane, with carboxyl and carbonyl groups at the edges [168]. Those oxygen functional groups impair electrical conductivity of graphene, causing GO to become an insulator. Therefore, the oxygenated groups of GO must be removed in order to restore its electrical conductivity. Several reducing agents, such as hydrazine hydrate, sodium borohydride, and hydroquinone can reduce GO into reduced graphene oxide (RGO) [169,170]. However, those agents remove oxygenated groups of GO to a certain degree. Thus RGO still bears a small fraction of residual oxygen groups. Among those reductants, hydrazine hydrate is commonly used for forming RGO, but it is highly toxic and explosive. Alternatively, RGO can be obtained through hydrothermal reduction of GO in an aqueous sulfuric acid suspension [171].

**Figure 7:**
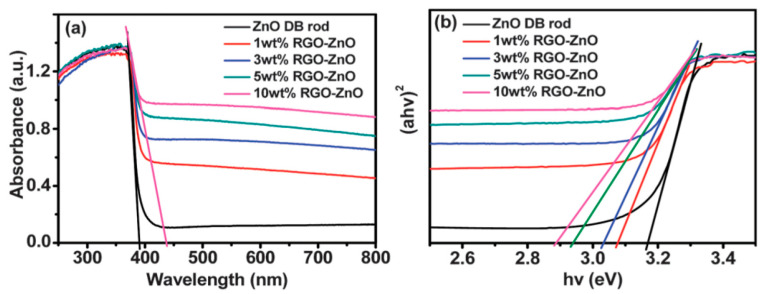
(**a**) UV-vis diffuse reflectance spectra, and (**b**) the plots of Kubelka–Munk function versus light energy for neat ZnO nanorod and RGO/ZnO nanocomposites. Reproduced from [172] with permission of the Royal Society of Chemistry.

Graphene oxide sheet with the lateral size up to several micrometers acts as a 2D template for nucleating ZnO crystals during the synthesis process. So oxygenated groups of GO favor the nucleation of ZnO nanocrystals by interacting with zinc salt precursor. As such, Zn^2+^ ions released from the Zn salt precursor tend to interact with the negatively charged oxygen functional groups on GO via electrostatic interactions. Consequently, oxygenated groups are removed from GO, leading to the formation of RGO-ZnO nanocomposites [161,172]. Figure 7a shows the typical optical absorbance of RGO/ZnO nanocomposites. The enhanced absorbance of nanocomposites with increasing RGO content in the visible light range can be readily observed. Without RGO, neat ZnO nanorod has a low absorbance in the visible light as expected. The bandgap of neat ZnO is 3.17 eV, and decreases to 3.07, 3.03, 2.94, and 2.88 by modifying with 1%, 3%, 5%, and 10% RGO, respectively (Figure 7b). Similarly, Wu et al. also indicated that strong interactions between ZnO and GO during hydrothermal synthesis favor the formation of visible-light active RGO-ZnO nanocomposites [173].

**Figure 8:**
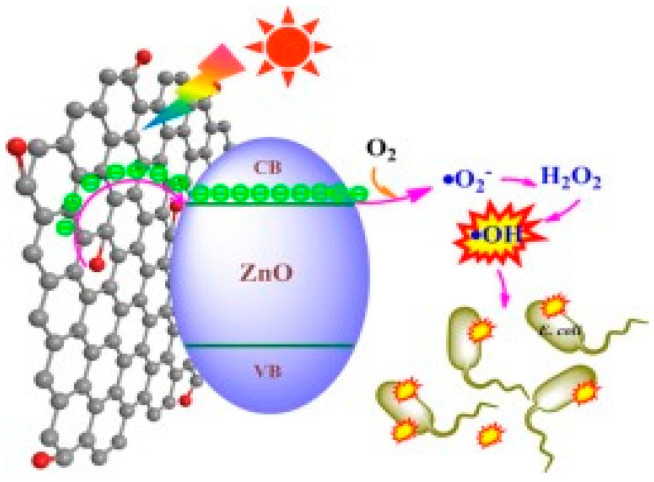
Electron transfer from graphene sheet to the conduction band of ZnO under visible light for generating ROS. Reproduced from [173] with permission of Elsevier.

As mentioned, graphene modification is an effective way to prevent photocorrosion of ZnO [89,160,161,162]. Under visible light irradiation, photoexcited electrons from the graphene sheet are injected into the CB of ZnO NPs. Consequently, those electrons reduce surface oxygen molecules through a series of reactions to produce superoxide anion, hydrogen peroxide and hydroxyl radical for bacterial inactivation (Figure 8) [173]. In this context, photocorrosion resulting from the ZnO degradation due to the attack of positively charged holes as described by the reaction (1) and reaction (2) can be avoided. Therefore, graphene sheet plays the role of a photosensitizer by directly injecting photoexcited electrons into the CB of ZnO under visible light without inducing positively charged holes. On the other hand, graphene with excellent electrical conductivity also acts as an electron acceptor for ZnO. By irradiating ZnO with UV light, photogenerated electrons are holes are created in the CB and VB, respectively. The electrons are then transferred from the CB of ZnO to graphene sheet, thereby inhibiting the recombination of electron-hole pairs, enhancing photocatalytic activity and photostability of ZnO nanostructures. On the other hand, photogenerated holes in the VB of ZnO react with the adsorbed hydroxyl group to give reactive hydroxyl species. As a result, photogenerated holes would not destruct the structural integrity of ZnO by degrading the Zn-O bond for releasing Zn^2+^ from the surface [87]. This implies that the photocorrosion of ZnO nanostructures can be prevented under UV irradiation effectively by modifying with graphene.

#### 2.3.2. Carbon Nanotubes

**Figure 9:**
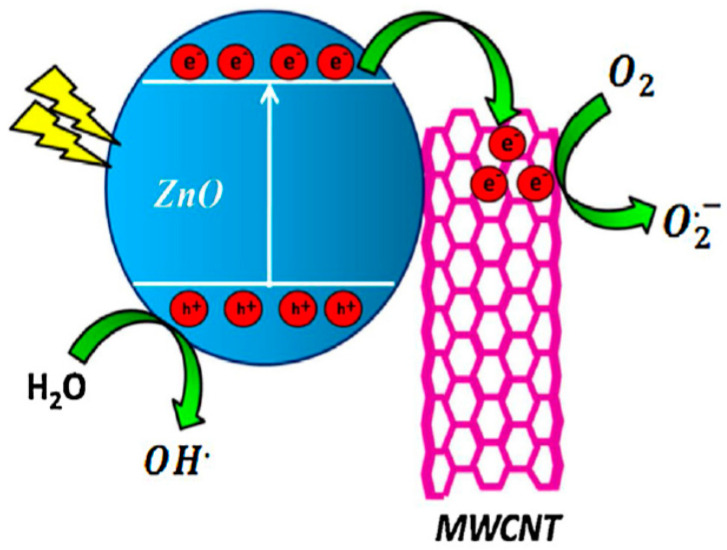
The transfer of photogenerated electron from the conduction band of ZnO to a single nanotube under UV irradiation. Reproduced from [175] with permission of Elsevier.

Carbon nanotubes with large aspect ratios are inert, exhibiting no oxygenated functional groups on their surfaces. However, hydroxyl and carboxyl functional groups can be formed on CNTs by oxidizing in a mixture of H_2_SO_4_/HNO_3_ (3:1; *v*/*v*) [174]. Analogously, those oxygen functional groups can react with Zn^2+^ ions deriving from the dissolution of ZnO salt precursor in an aqueous solution for nucleating ZnO nanocrystals. This leads to homogeneous dispersion of ZnO NPs on the CNTs. Under the UV exposure, photogenerated electron from the CB of ZnO is transferred to the CNT. Therefore, CNT acts as an effective electron trapping site, hindering the recombination of electron–hole pair (Figure 9) [175]. On the contrary, photoexcited electron is injected from the CNT to the CB of ZnO under visible light irradiation for triggering the formation of ROS.

### 2.4. Semiconductor Heterojunctions

Coupling ZnO with semiconductors of different bandgap energies is considered to be an effective strategy to form visible-light-driven photocatalysts. ZnO can form heterojunctions with other semiconductors such as copper oxide (CuO), titania (TiO_2_), tin oxide (SnO_2_), cadmium sulfide (CdS), and zinc sulfide (ZnS). Some semiconductors have a larger bandgap energy than ZnO. So they are unfavored materials for coupling with ZnO to form visible-light-activated photocatalysts. As an example, the bandgap energy of SnO_2_ is 3.67 eV [176], thus facilitating the formation of UV-responsive ZnO/SnO_2_ heterojunctions [177]. Titania is an UV-active semiconductor with a bandgap energy of 3.2 eV [178,179]. ZnS with a wide bandgap of 3.6–3.8 eV absorbs UV light only [180]. CdS is a visible-light active semiconductor with a narrow bandgap of 2.40 eV [180]. However, CdS is a highly toxic carcinogen, and thus unfavorable to form CdS/ZnO heterojunction based on public health considerations. On the other hand, CuO and iron oxide (α-Fe_2_O_3_) have a relatively narrow bandgap of 2.00 eV and 2.20 eV, respectively [180,181], so they are ideal metal-oxide semiconductors for coupling with ZnO to yield visible-light photocatalyts.

**Figure 10:**
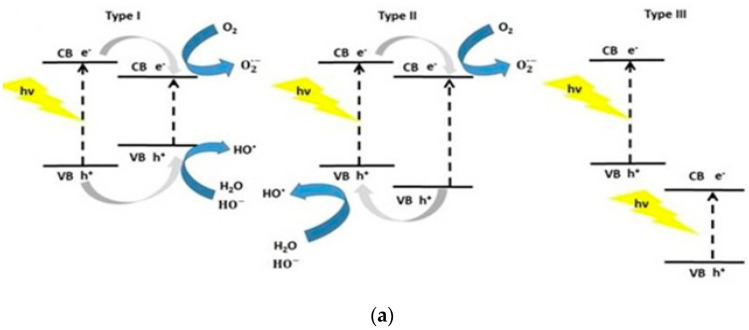

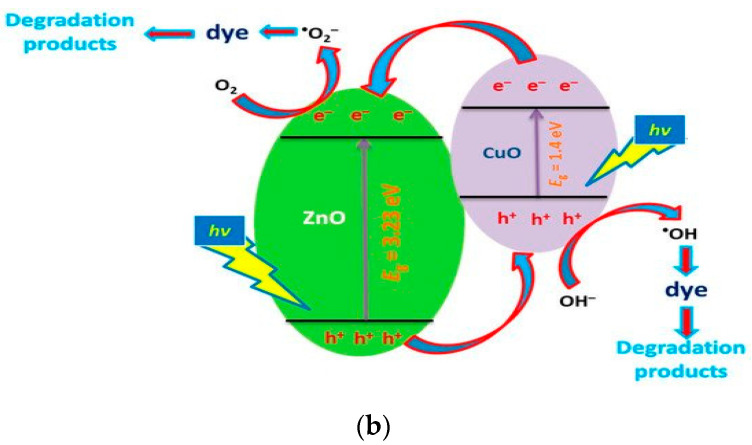
(**a**) Energy band alignments of type-I, type-II, and type-III heterojunctions. Reproduced from [186] with permission of Frontiers under the Creative Commons Attribution License. (**b**) The charge carrier transfer in CuO/ZnO photocatalyst under sunlight irradiation. Reproduced from [185] the terms of the Creative Commons Attribution License.

The visible-light photocatalytic performance of semiconductor heterojunctions depends greatly on the energy band alignment of the CB and VB of two dissimilar materials. Certain conditions must be met for achieving improved photocatalytic activity of semiconductor heterostructures under visible light. There exists three types of coupled heterojunctions, i.e., type-I, type-II and type-III in terms of the alignment of their energy levels (Figure 10a) [181,182,183,184,185,186]. In type-I heterostructure, the CB and VB levels of the smaller bandgap semiconductor lie between those of a wider bandgap semiconductor. Therefore, photoexcited electron and hole from the larger bandgap material migrate across the heterojunction and accumulate at the CB and VB of smaller bandgap semiconductor under visible light irradiation, producing ROS accordingly. In type-II heterostructure, both the CB and VB of narrow-gap semiconductor lie above those of large bandgap material. In this respect, photoexcited electron from the narrow-gap semiconductor moves to the large bandgap material, while the hole migrates from the large-gap material across the junction in the opposite direction. Thus type II heterojunction is very effective to promote the charge carrier separation, thereby reducing the recombination of electron-hole pairs. The CuO/ZnO and Cu_2_O/ZnO systems are typical examples of the type-II heterostructure [90,185]. For the CuO/ZnO system, photogenerated electron from the CB of CuO is transferred to the CB of ZnO for generating superoxide anion, while the hole from the VB of ZnO is migrated to the VB of CuO for creating hydroxyl radical (Figure 10b). The formation of type-II CuO/ZnO heterojunction improves the separation of photogenerated electron-hole pair, leading to enhanced photocatalytic activity under visible light [185]. In type-III heterostructure, the bandgaps of two dissimilar semiconductors do not overlap at all. So the transfer of electron and separation of electron-hole pair are unlikely to occur at the heterojunction.

## 3. Synthesis of ZnO Nanostructures

### 3.1. Vapor Phase Route

Zinc oxide of zero-, one-, two- and three-dimensional nanostructures can be synthesized by means of vapor-, liquid-, and solid-phase processing techniques. Conventional French process is a straightforward method to fabricate ZnO nanostructures by melting zinc at 1000–1400 °C in an air or oxygen atmosphere. As such, zinc vapor chemically reacts with oxygen to form ZnO with a purity of up to 99.9%, and sizes ranging from about 0.1 µm down to the nanoscale dimension. The ZnO nanostructures produced include nanorods, nanoplates, nanoboxes, polyhedral drums and irregularly-shaped particles. Those nanostructures generally grow either along the <0001> or <1010> plane directions [186]. The ratio between the Zn vapor pressure and oxygen pressure, or the Zn/O flux ratio must be carefully monitored for attaining the desired ZnO nanostructures [187]. Very recently, Hanif et al. reported a facile synthesis of spherical ZnO NPs (21.31 nm) with high crystallinity through the thermolysis of zinc acetate dihydrate [Zn(CH_3_COO)_2_.2H_2_O] under high temperatures and pressures [188].

The vapor-phase methods include physical vapor deposition (PVD), chemical vapor deposition (CVD), and molecular beam epitaxy (MBE). PVD techniques include sputtering, thermal evaporation and pulsed laser deposition [189]. Sputtering is a typical plasma-assisted PVD technique in which collisions between gaseous ions and the target material resulting in the ejection of atoms from the target surface [190]. A typical inert gas such as argon is ionized under the application of direct-current (DC), or radio-frequency (RF) voltages. The inclusion of additional magnetic field further increases the ionization rate of inert gas, and guides the ion flux towards the substrate. So magnetron sputtering employs strong electric and magnetic fields to trap electrons close to the target surface to deposit ZnO film on the substrate. Magnetron sputtering offers the advantages of high deposition rate and low substrate temperature, but suffers from the high equipment cost and high defect concentration in the deposited films [191]. Pulsed laser deposition (PLD) is based on the ablation of target material placed inside the vacuum chamber using a laser source [192]. A high-power laser beam strikes ZnO or pure Zn target, resulting in its local heating and evaporation. This leads to the deposition of high purity ZnO film on the substrate [193,194,195]. PLD is simple and versatile, however, the use of high vacuum deposition chamber and high-power laser source, as well as the particulate emission during the target ablation are the major drawbacks of this process [196,197].

**Figure 11:**
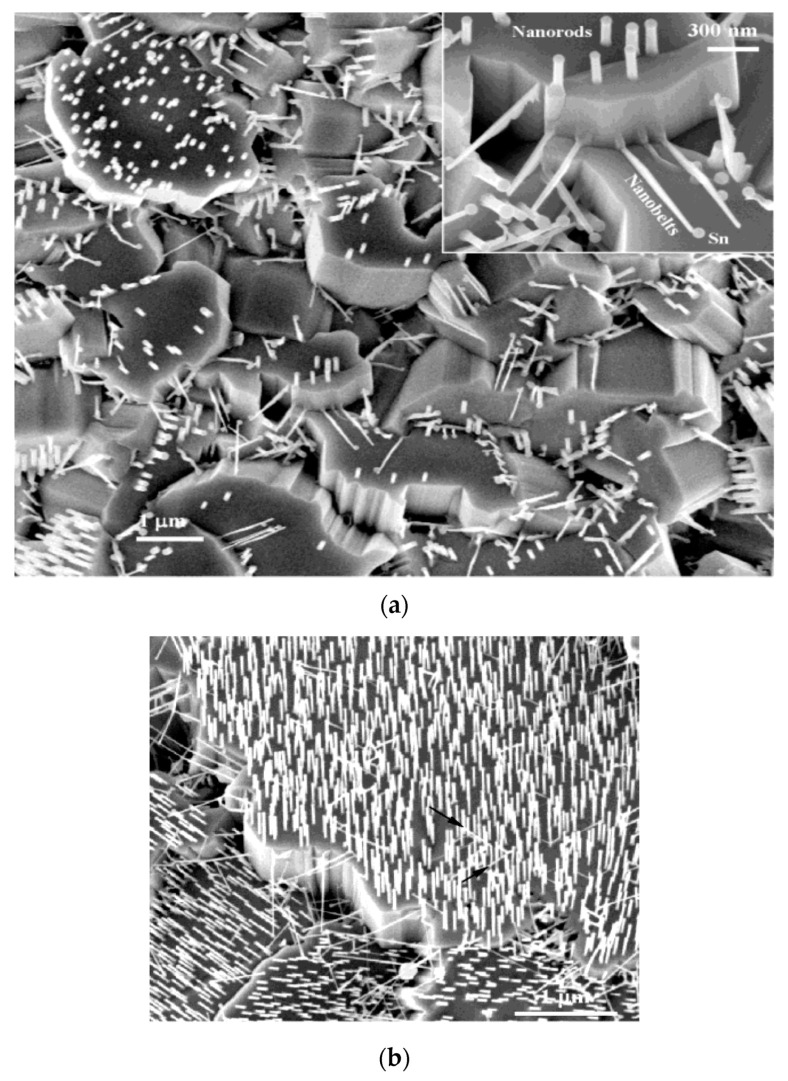
(**a**) Scanning electron microscope (SEM) image showing ZnO nanorods and nanobelts grown on the top (0001) and side surfaces of the ZnO microrods, respectively for 15 min. Inset: Magnified SEM image. (**b**) SEM image of ZnO nanorods and nanobelts after growing for 30 min at 1100 °C. Black arrows indicate nanobelts. Reproduced from [205] with permission of the American Chemical Society.

As it is known, atomic layer deposition (ALD) and chemical vapor deposition (CVD) are widely used for a large-scale production of thin films for electronic and optoelectronic device applications. In particular, ALD has been employed for forming a large range of metal oxide films including ZnO [198,199,200]. In the process, chemical precursor reactants (e.g., diethylzinc and water vapor) are alternatively introduced into the growth chamber, and react sequentially on desired substrate surfaces to form nano-ZnO films of uniform and reproducible thickness, even at low deposition temperatures of 25–300 °C [199,200]. Recently, 1D-ZnO nanostructures have received considerable attention because of their potential applications in advanced optoelectronic devices. The CVD method is an effective tool to fabricate 1D-ZnO using Zn source material, i.e., a mixture of ZnO/C powder in the presence of metal catalyst such as Au or tin. The process requires high-temperature heat unit deriving from thermal, microwave or radio frequency induced plasma [201]. Vaporization of ZnO/C is achieved through carbothermal reduction of ZnO/C powder at elevated temperatures. In the process, i.e., Zn or ZnO/C powder is placed inside a furnace quartz tube, and heated at 800–1000 °C to achieve zinc vapor saturation. The substrate (sapphire or silicon) is coated with Au catalyst. The growth of 1D-ZnO nanostructures has been ascribed to the vapor-liquid-solid (VLS) mechanism [202,203]. The condensation of zinc vapor on Au results in the formation of liquid Au-Zn alloy droplet. Subsequently, supersaturation of the liquid with Zn vapor leads to the nucleation of crystals at the liquid-solid interface, facilitating the growth of 1D-ZnO [204]. As mentioned, Zn-terminated (0001) surface with a higher surface energy (γ_zn_ = 2.49 J/m^2^) is more active than the O-(0001) plane having a lower surface energy (γ_O_ = 1.35 J/m^2^) [204]. Thus the Zn-(0001) plane provides an effective site for nucleating crystals, leading to anisotropic growth of ZnO nanorods along the c-axis direction (Figure 11a,b) [74,205]. In other words, the growth rate is faster on the Zn-(0001) plane having higher surface energy, and so vertically aligned nanorods grow preferentially along the <0001> direction.

**Figure 12:**
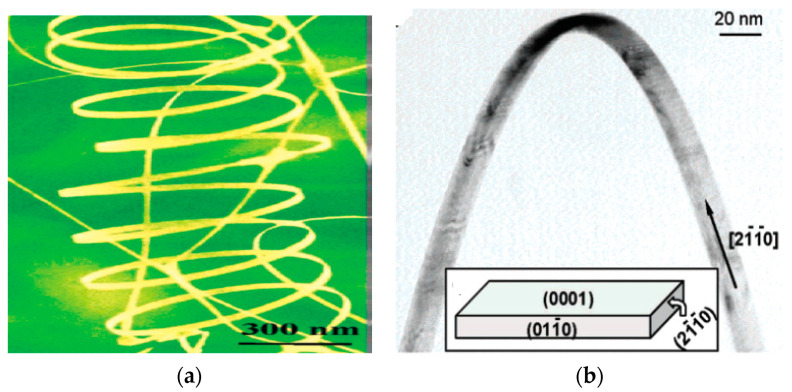
(**a**) SEM image of ZnO nanobelts. (**b**) Transmission electron micrograph of a helical nanobelt. Inset: structural model of ZnO nanobelt. Reproduced from [208] with permission of the American Chemical Society.

One-dimensional ZnO nanostructures can also be synthesized without using metal catalysts. In this respect, zin vapor condenses directly onto the surface of a solid substrate, and the vapor-solid (VS) mechanism is responsible for the growth of catalyst-free ZnO nanostructures [79,206,207]. As the VS grown ZnO nanostructures are catalysts free, thus it is more difficult to control over their morphology than those formed from the VLS mechanism using the catalysts. Several processing parameters such as temperature, oxygen and argon flow rate, amount of precursor, growth time, and type of the substrates, affect the morphology of nano-ZnO greatly. In particular, the growth temperature plays a key role to regulate the morphology of nano-ZnO ranging from polycrystalline thin films to nanowhiskers [207]. Very recently, Karnati et al. employed high pressure assisted pulsed laser deposition to form vertically aligned ZnO nanorods on various substrates. Temperature and number of pulsed laser shots during the deposition play important roles in the alignment of ZnO nanorods [79]. Kong and Wang reported that 1D-ZnO of various morphologies such as nanobelts, nanowires, nanorings, nanohelixes/nanosprings, nanocombs, nanobows, and nanocages can be synthesized under specific growth conditions using a solid-vapor phase thermal sublimation method [208,209]. Helical ZnO nanobelts with top and bottom flat surfaces consist of ±(0001) polar surfaces serve as the building blocks to form nanorings, nanohelixes and nanosprings (Figure 12) [208,209].

### 3.2. Liquid-Phase Route

#### 3.2.1. Co-Precipitation Method

The liquid phase synthesis can be used to fabricate ZnO nanomaterials in an aqueous or organic medium using a wide range of precursors under different processing conditions. Liquid phase synthesis has many advantages over vapor phase route for fabricating ZnO nanostructures in terms of simplicity, versatility, low cost, low temperature and scalability. The solution phase synthesis generally includes co-precipitation, sol-gel, hydrothermal/solvothermal treatment, and microemulsion. ZnO nanostructures with different dimensions such as spherical nanoparticles, nanoprisms, nanorods, nanowires, nanodisks and nanoflowers can be obtained by controlling the synthetic parameters such as the types of metal salt precursor and solvent, zinc salt concentration, solution pH and temperature, reaction time, surfactant type and concentration, ZnO seed layer utilization, etc.

Co-precipitation method involves the dissolution of a metal salt precursor in water/organic solvent for precipitating oxo-hydroxide in the presence of a strong alkali metal hydroxide (MOH; M = Li, Na, K, Cs), or weak hexamethylenetetramine (HMTA) [210,211,212,213,214]. In the process, zinc metal salt precursor decomposes in the solution to yield Zn^2+^ for forming Zn(OH)_2_. By adding MOH dropwise, Zn(OH)_2_ dissolves in the solution due to an increase of the basicity, and reacts with OH^−^ to give [Zn(OH)_4_]^2–^. The [Zn(OH)_4_]^2–^ complex is generally known as the growth unit, serving as the nucleation site for growing ZnO crystals by precipitating on positively charged Zn-terminated (0001) surface [76,77,210]. Nano-ZnO is formed via dehydration of [Zn(OH)_4_]^2−^. When hexamethylenetetramine [(CH_2_)_6_N_4_] is used instead of MOH, thermal decomposition of HMTA generates ammonia and formaldehyde (HCHO). Ammonia serves as a weak base by accepting hydrogen ions from water to produce ammonium NH_4_^+^ and OH^−^ ions. Hydroxyl ions are needed for triggering the precipitation reaction. Zinc ions from zinc salt precursor react with hydroxide ions to form Zn(OH)_2_ and [Zn(OH)_4_]^2−^. Furthermore, zinc ions also react with ammonia to yield tetraamminezinc(II) complex (Zn-tetraamine), i.e., [Zn(NH_3_)_4_]^2+^. The [Zn(NH_3_)_4_]^2+^ complex also acts as the growth unit for ZnO nuclei. Overall, the reactions are given as follows [210,211],

(CH_2_)_6_N_4_ + 6H_2_O → 6HCHO + 4NH_3_ (5)

NH_3_ + H_2_O → NH_4_^+^ + OH^−^ (6)

Zn(OH)_2−_+ 2OH^−^ →[Zn(OH)_4_]^2−^ (7)

[Zn(OH)_4_]^2−^ → ZnO + H_2_O + 2OH^−^ (8)

Zn^2+^ + 4NH_3_ → [Zn(NH_3_)_4_]^2+^ (9)

[Zn(NH_3_)_4_]^2+^ + 2OH^−^ → ZnO + 4NH_3_ + H_2_O (10)

**Figure 13:**
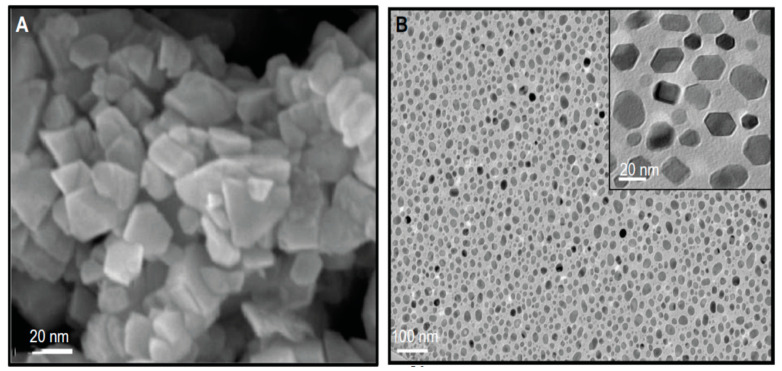
(**A**) SEM and (**B**) transmission electron microscope (TEM) images of ZnO nanoparticles (NPs) prepared by co-precipitation process using zinc acetate dihydrate and NaOH. Inset in (**B**) shows a high-magnified TEM image of hexagonal shaped ZnO NPs. Reproduced from [212] under a Creative Commons License.

Pourrahimi et al. dissolved several zinc salt precursors like zinc nitrate, chloride, sulfate and acetate in aqueous solutions in the presence of NaOH to synthesize nano-ZnO [215]. The acetate precursor produces nanoprismatic particles with a narrow size distribution having an average size of 25 nm. The chloride and sulfate precursors give rise to ZnO NPs with sizes of 10–30 nm, and larger particles (petals) with sizes of 80–100 nm, respectively. The petals derive from the self-assembly of nanoprisms along the c axis of ZnO. For zinc nitrate salt, the petals link each other into flower-shaped particles with an average size of 500 nm. Furthermore, acetate ions from zinc acetate are effective to stabilize nanoprisms during the crystal growth, thereby preventing their assembly to large petals. However, nitrate counter-ions favor the growth of nanoprisms into large petals and flower-like crystals. Akhtar et al. fabricated ZnO NPs by reacting zinc acetate dihydrate (Zn(CH_3_COO)_2_ · 2H_2_O) with NaOH [212]. Prismatic ZnO NPs with hexagonal/polygonal shapes having an average size of 21.34 nm are precipitated in the solution accordingly (Figure 13A,B) [212].

##### Biosynthesis

**Figure 14:**
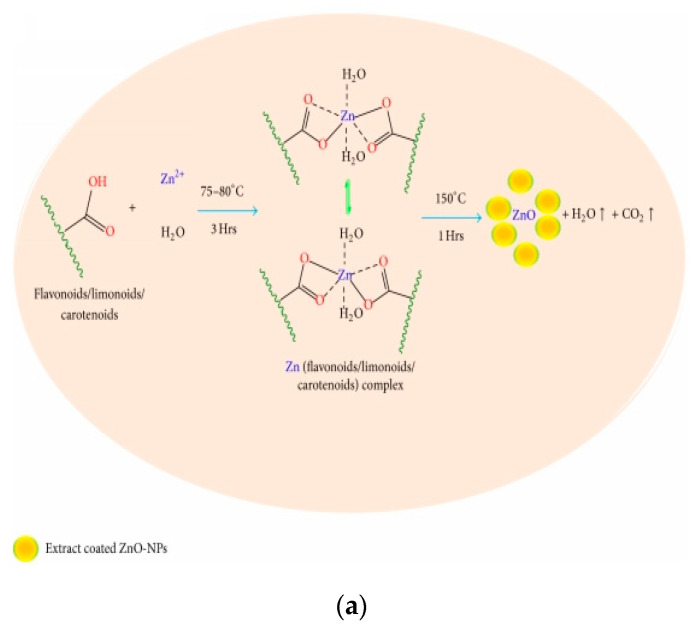

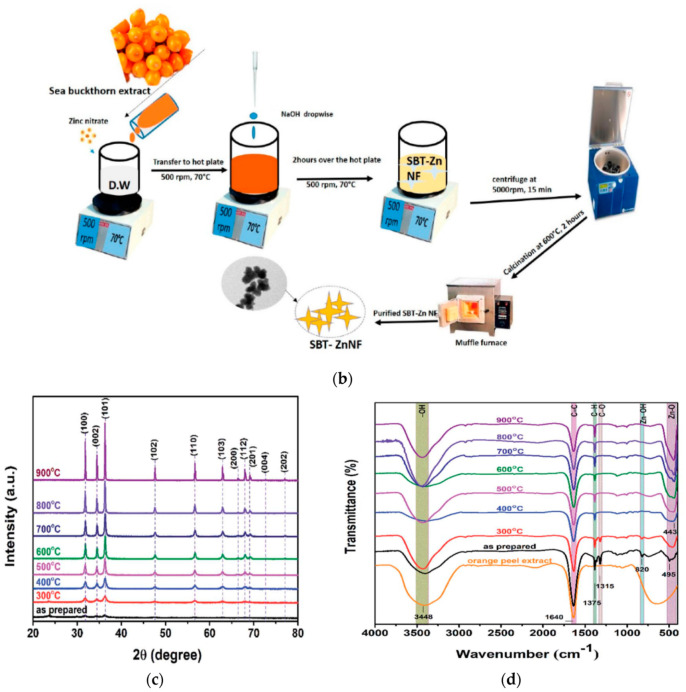
(**a**) Proposed reaction mechanism between functional groups of grapefruit peel extract and zinc ions from zinc sulfate in forming zinc-ligand complex and ZnO NPs after drying in an oven at 150 °C. Reproduced from [217] under the Creative Commons Attribution License. (**b**) A schematic showing biosynthesis of ZnO nanoflowers (NFs) using Zn(NO_3_)_2_.6H_2_O and *sea buckthorn* (SBT) fruit extract. Reproduced from [220] under the terms of the Creative Commons Attribution License. (**c**) X-ray diffraction patterns showing the wurtzite structure of as-synthesized ZnO nanoparticles calcined at different temperatures. ZnO NPs are biosynthesized from Zn(NO_3_)_2_.6H_2_O and orange peel extract. (**d**) Fourier transform infrared spectra of orange peel extract and the as-synthesized ZnO NPs heat treated at different temperatures. Reproduced from [219] under the terms of the Creative Commons Attribution-NonCommercial 3.0 Unported License.

Comparing with chemical reagents, toxic free and eco-friendly plant extracts are cost competitive agents for preparing ZnO NPs [216,217,218,219,220]. The plant-based organic compounds are abundant and renewable bioresources. The phytochemical constituents such as alkaloids, flavonoids, phenols, amino acids, polysaccharides, and tannins extracted from various parts of plants like leaves, fruits, seeds and flowers can be used for driving reduction reactions during the ZnO synthesis. They also act as a capping agent/stabilizer for forming ZnO NPs. In some cases, hydroxyl and carboxylic groups of citrus fruit peel extracts (e.g., flavonol and carotenoid) serve as natural ligand agents for zinc ions from zinc salt precursor [217,218,219]. For instance, Fumar et al. proposed the reactions between functional groups of grapefruit peel extract and zinc ions from zinc sulfate precursor in forming a zinc-ligand complex as given in Figure 14a [217]. Figure 14b shows a facile and eco-friendly process for synthesizing ZnO nanoflowers using zinc nitrate and *sea buckthorn* fruit extract [220]. Apprarently, biosynthesis of ZnO nanoflowers is a single step process via the reactions between the functional groups of *sea buckthorn* fruit extract and zinc nitrate precursor. The resultant mixture is collected by centrifugation at 5000 rpm for 15 min followed by calcination in a muffle furnace at 600 °C for 2 h to form ZnO nanoflowers. Very recently, Thi et al. employed an aqueous extract of orange peel and zinc nitrate to synthesize ZnO NPs for antibacterial purposes [219]. The orange peel extract phytochemicals (e.g., flavonoid, limonoid, carotenoid) act as natural ligand agents. So hydroxyl aromatic ring groups from the extract tend to form a ligand complex with zinc ions from zinc nitrate. The reaction product is then dried at 150 °C followed by calcination at 300–900 °C to yield ZnO NPs. During drying and calcining stages, zinc ligand complex converts into ZnO NPs accordingly. The as-synthesized ZnO NPs exhibit low crystallinity, and their crystallinity improves with increasing calcination temperatures (Figure 14c) [219]. Fourier transform infrared spectra also reveal the peak intensity associated with the stretching vibration of Zn–O at around 450 cm^−1^ increases while the band intensity due to organic functional groups C=C and C=O of orange peel extract at 1640 cm^−1^ decreases with increasing calcination temperatures. A broad band centered at 3448 cm^−1^ is ascribed to adsorbed water molecules on ZnO NPs (Figure 14d). Apparently, FTIR spectra confirms the presence of pell extract residuals on the nanoparticles upon calcination for different temperatures. Those phytochemicals are beneficial in assisting ZnO NPs for killing various bacterial strains.

##### ZnO Seed Layer

**Figure 15:**
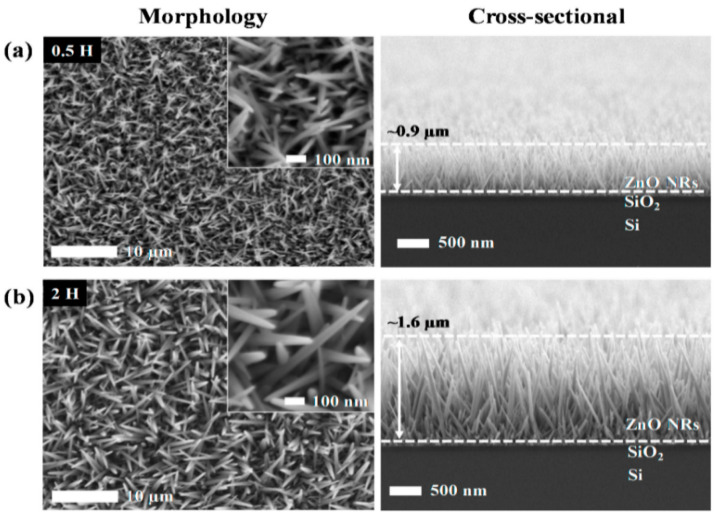
SEM images of top and cross-sectional views of ZnO nanorods formed on ZnO-seeded SiO_2_/Si substrate by immersing in zinc acetylacetonate hydrate and HMTA solution for (**a**) 0.5 h and (**b**) 2 h. Insets in left panels of (**a**,**b**): High-magnification plan-view SEM images. Right panels: cross-sectional SEM micrographs. Reproduced from [223] under the terms of the Creative Commons Attribution License (CC BY).

Generally, ZnO nanorods prepared from co-precipitation without using a seeded substrate are oriented randomly. Thus, a two-step approach, including the synthesis of a thin seed layer on the substrate and co-precipitation in aqueous solutions, has been adopted widely to prepare vertical aligned ZnO nanorods/nanowires. The seed layer serves as the nucleation site for vertical growth of dense ZnO nanostructures [200,210,221,222,223]. ZnO seed layer can be deposited on desired substrates using PLD, ALD, spin coating and sol-gel techniques [200]. For instance, Liu et al. first deposited a thin ZnO seed layer on the glass and indium tin oxide (ITO) respectively, followed by growing ZnO nanorods on those substrates upon immersion in a solution of Zn(NO_3_)_2_ and HMTA [221]. Without ZnO seeding, the sizes of ZnO nanorods grown on bare substrates are larger than those on ZnO-seeded substrates. This is due to few nucleation sites are available for forming crystals on bare substrates. In contrast, the coverage density of ZnO nanorod arrays is much higher on the seeded substrates as expected. More recently, Karim et al. synthesized ZnO nanorod arrays on ZnO-seeded SiO_2_/Si substrate by dipping in an aqueous solution of zinc acetylacetonate hydrate and HMTA at 85 °C for 0.5–4 h [223]. High-density of vertically aligned nanorods are grown on the ZnO-seeded substrate for 0.5 h. The length of ZnO nanorod array is ~0.9 µm (Figure 15a). By increasing immersion time to 2 h, the length of ZnO nanorods is increased to ~1.6 µm (Figure 15b). As aforementioned, aligned ZnO nanorods can be prepared using vapor-phase CVD process. However, CVD requires a relatively high synthetic temperature, long deposition time, and large energy consumption for forming ZnO nanorods. Apparently, the liquid-phase solution synthesis offers a good opportunity for forming aligned ZnO nanorods at relatively low temperatures and low cost.

Co-precipitation process generally yields a large particle size distribution, and poor control on the particle shape formation. Moreover, ZnO NPs tend to agglomerate into clusters during the synthesis. In this respect, capping agents or surfactants are added into zinc salt precursor solutions to control the morphology and size of ZnO nanostructures, and to prevent the aggregation of ZnO nanocrystals. Typical capping agents include cetyl trimethyl ammonium bromide (CTAB), sodium dodecyl sulfate (SDS), Triton X-100, oleic acid, elaidic acid, etc. [224,225,226]. Cationic CTAB and anionic SDS surfactants are typically used for stabilizing colloidal NPs. Ramimoghadam et al. demonstrated that the mixture of cationic-anionic CTAB and SDS surfactants can significantly modify the shape and size of ZnO NPs [225]. However, CTAB is highly toxic, and SDS is a hazardous agent [227]. Thus their use for stabilizing colloidal nanoparticles must be restricted. In this respect, recent attention has paid to the use of green chemical agents extracted from the plants such as ascorbic acid and citric acid for stabilizing colloidal ZnO NPs [228,229].

#### 3.2.2. Hydrothermal/Solvothermal Synthesis

**Figure 16:**
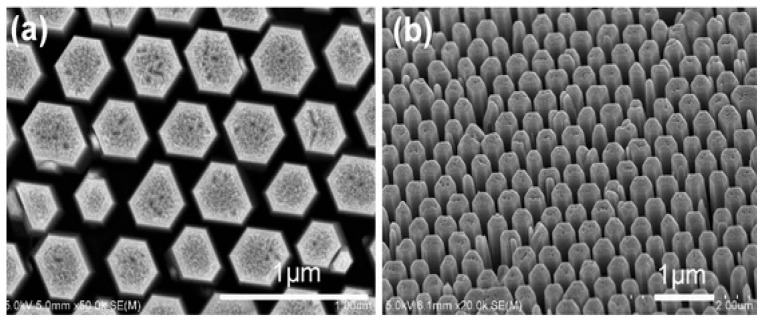
SEM images showing (**a**) top view and (**b**) 45° tilt view of ZnO nanorod arrays. Reproduced from [230] under the terms of the Creative Commons Attribution 4.0 International License.

The hydrothermal/solvothermal strategy utilizes water or organic solvent as the reaction medium for synthesizing ZnO nanostructures in a sealed autoclave system at moderate to high reaction temperatures. The recent studies on hydrothermal/solvothermal synthesis of ZnO nanostructures/nanocomposites of different geometries have been reported elsewhere [230,231,232,233,234,235,236,237]. In some cases, surfactants are added to control the size and shape of nano-ZnO synthesized under hydrothermal or solvothermal treatment [225,226,234]. ZnO nanostructures can be synthesized hydrothermally with or without ZnO-seeded substrates. For instance, Dong et al. prepared ZnO nanorod arrays using ZnO seeded ITO substrate, Zn(NO_3_)_2_·6H_2_O precursor and HMTA weak base [230]. Figure 16a,b are the SEM images showing the top view and 45° tilt view of ZnO nanorod arrays grown on ITO in 0.05 M Zn(NO_3_)_2_·6H_2_O and HMTA solution at 80 °C for 3 h. The nanorod arrays with a hexagonal feature are oriented normal to the substrate.

**Figure 17:**
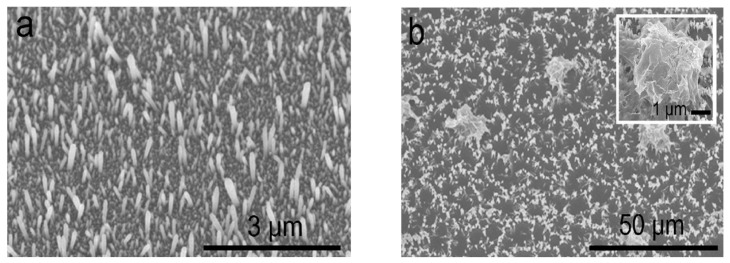

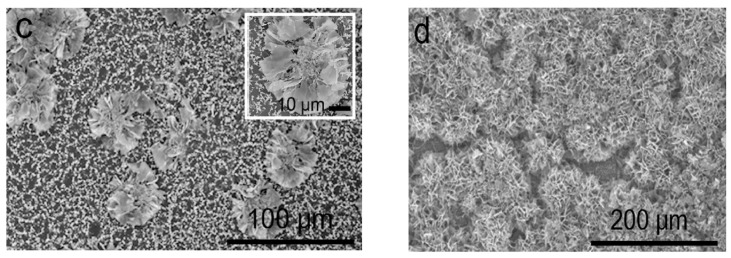
The effect of growth time on morphological evolution of flower-like ZnO nanostructures in preheated aqueous Zn(NO_3_)_2_·6H_2_O and HMTA solutions: (**a**) 2 h, (**b**) 4 h, (**c**) 6 h, and (**d**) 24 h. Reproduced from [231] under the Creative Commons Attribution License.

Qiu et al. prepared ZnO nanoflowes using a surfactant-free hydrothermal process. Thin-ZnO film coated glass slides, Zn(NO_3_)_2_·6H_2_O, and HMTA were used, and the mixed reagent solutions were preheated at 95 °C for 12h [231]. In the process, ZnO-coated glass substrates are immersed in preheated aqueous solutions for different time periods. As such, ZnO nanorods are grown on the seeded glass slide along <0001> direction after dipping for 2 h (Figure 17a). By increasing the growth time to 4 h, secondary nucleation takes place on nanorods, and the rods contact each other to form the petals [238]. Those petals then link together to form corolla (Figure 17b and inset). More flower-like ZnO can be readily observed as the growth time increases to 6h (Figure 17c and inset). Thus the formation of ZnO nanoflower derives from self-assembly of zincates into nonorods, and secondary nucleation on nanorods to form the petals. Further increasing the reaction time to 24 h leads to a complete formation of numerous nanoflowers on seeded glass substrate (Figure 17d).

**Figure 18:**
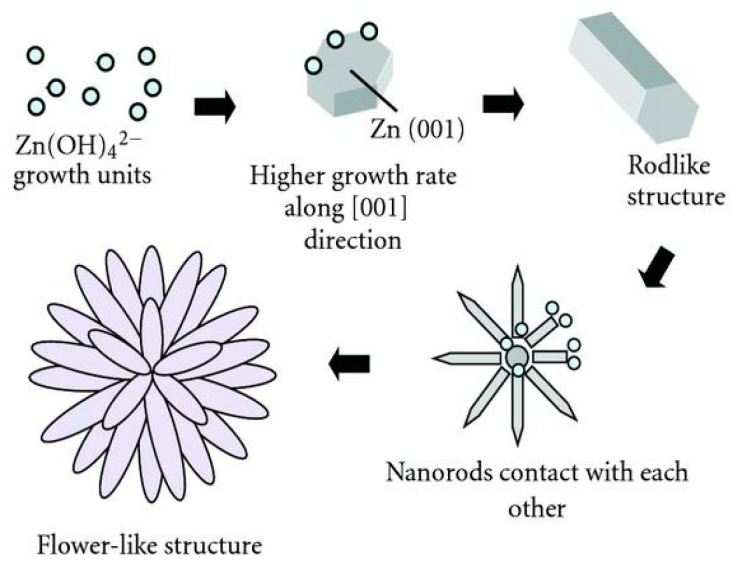
Schematic displaying the evolution of ZnO nanorods and ZnO nanoflowers from [Zn(OH)_4_]^2−^ growth units. Reproduced from [239] under the Creative Commons Attribution License.

As aforementioned, polar Zn-terminated ZnO (0001) with a higher surface energy and reactivity than polar O-(0001), non-polar (2110) and (0110). Thus Zn-terminated (0001) plane attracts negatively charged [Zn(OH)_4_]^2−^ to its surface during the synthesis process. The amount of [Zn(OH)_4_]^2^ deposited on Zn-(0001) surface increases with the growth time and temperature as expected. The growth units then self-assemble rapidly into nanoparticles on Zn-(0001) surface, and grow along the <0001> direction to form 1D nanorods as shown in Figure 17a. Secondary nucleation occurs on the nanorod surfaces as the crystallization time increases during hydrothermal synthesis [238]. These rods contact and link each other under a prolonged reaction time to form 3D-ZnO nanoflowers. Figure 18 is a schematic illustration showing the deposition of [Zn(OH)_4_]^2−^ on Zn-(0001) surface, the self-assembly and growth of crystals along the <0001> direction to form 1D nanorods, secondary nucleation on top of primary nanarods to yield flower flakes, and the final formation of ZnO nanoflowers [239].

**Figure 19:**
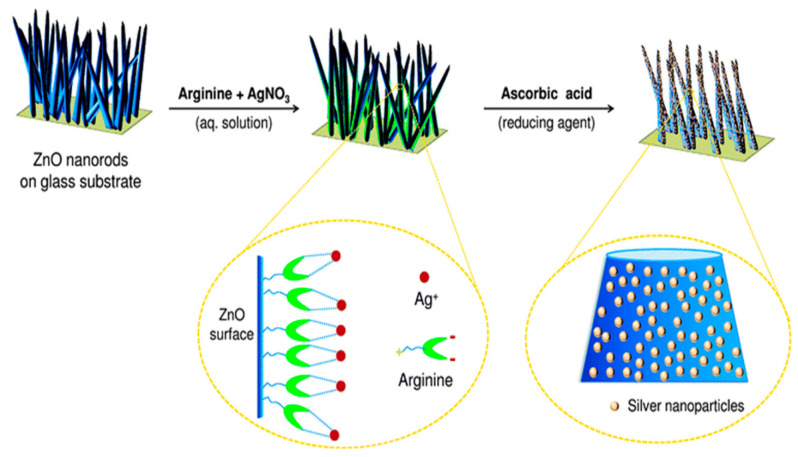
Schematic representation of the synthesis of AgNPs/ZnO nanocomposite using arginine as a linker to immobilize AgNPs on ZnO nanorods. Reproduced from [235] under a Creative Commons Attribution 3.0 Unported License.

**Figure 20:**
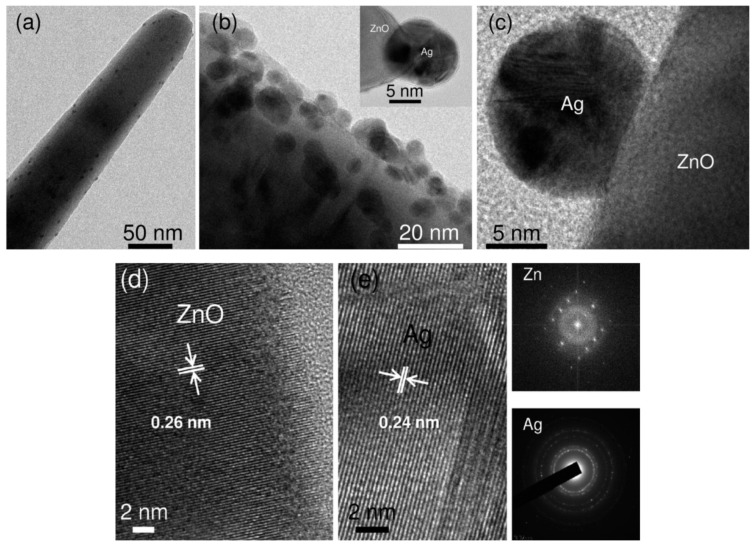
TEM images of (**a**) a single ZnO nanorod with uniformly dispersed AgNPs. (**b**) Side view of AgNPs/ZnO nanorod and immobilized AgNP at the tip of ZnO (inset). High-resolution TEM (HRTEM) images showing (**c**) a clear interface between AgNP and ZnO, (**d**) lattice fringes with characteristic d-spacing of ZnO, and (**e**) lattice fringes of AgNP. The corresponding electron diffraction patterns of ZnO (above panel) and AgNP (below panel) are also presented. Reproduced from [235] under a Creative Commons Attribution 3.0 Unported License.

Agnihotri et al. employed ZnO seeded microscope glass substrates to grow ZnO nanorods, followed by decorating ZnO nanorods with AgNPs [235]. In the process, ZnO seeded glass substrates were first immersed in an aqueous solution of zinc nitrate and HMTA at 90°C for 1h to produce ZnO nanorods. The as-grown ZnO nanorods were further hydrothermally treated in a mixed silver nitrate–arginine solution containing ascorbic acid to form AgNPs/ZnO nanocomposite. Ascorbic acid was used to reduce Ag^+^ ions to AgNPs, while arginine amino acid was added to immobilize AgNPs on ZnO nanorods (Figure 19). The morphology of AgNPs/ZnO nanorods and the dispersion of AgNPs on a single ZnO nanorod are shown in Figure 20a,b, respectively. High-resolution TEM (HRTEM) images of the AgNP-ZnO interface, lattice fringes of ZnO and AgNP are shown in Figure 20c,e, respectively.

**Figure 21:**
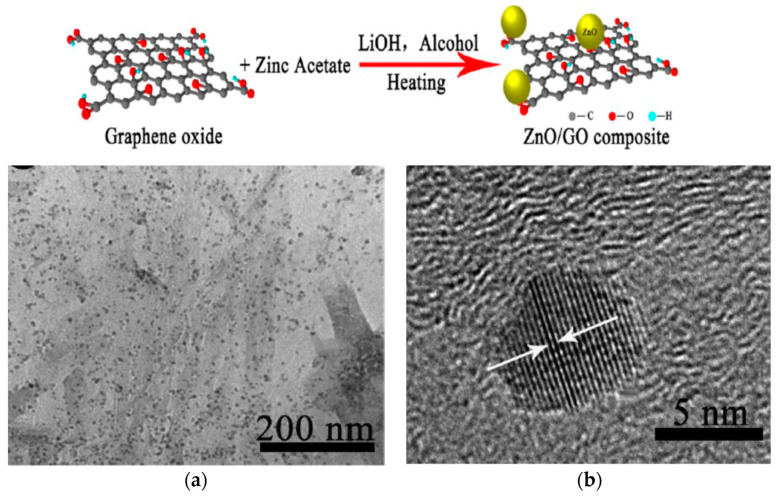
Formation of GO-ZnO nanocomposites using solvothermal process. (**a**) Transmission electron micrograph showing dispersion of ZnO nanoparticles on graphene sheet. (**b**) HRTEM image showing the lattice fringes of ZnO nanoparticle. Reproduced from [242] with permission of the American Chemical Society.

The formation of GO/ZnO nanocomposites is now considered. GO prepared by a modified Hummers process is thoroughly mixed with the solution of zinc salt and alkali hydroxide using either water or organic solvent [240]. The resulting solution is then treated hydrothermally [173,241], or solvothermally [242,243,244,245]. The oxygenated groups of GO are largely removed from graphene sheet during heating, leading to the formation of RGO [172,240,241,244]. In a certain case, ZnO NPs synthesized from co-precipitation is added directly to the GO solution [246]. Wang et al. synthesized GO/ZnO nanocomposites solvothermally by dissolving zinc acetate in alcohol followed by adding LiOH and GO (Figure 21). The obtained precipitate was centrifuged, washed and dried at 60 °C for 12 h in a vacuum oven. The GO/ZnO nanocomposites with a respective mass ratio of 1:3 and 1:2 were synthesized accordingly [242]. The interaction between the Zn^2+^ and negatively charged oxygenated groups of GO leads to the formation of finely dispersed ZnO NPs on the graphene sheet having a lateral dimension of micrometer scale (Figure 21a). High-resolution transmission electron micrograph (HRTEM) displays the lattice fringes of ZnO particle with a diameter of ~4 nm having a fringe distance of 0.28 nm (Figure 21b). This corresponds to the (100) spacing of ZnO.

#### 3.2.3. Hydrolysis and Condensation Process

The sol-gel process offers the advantage of simplicity for fabricating ZnO nanostructures of various morphologies [26,247,248,249,250,251,252]. Sol is the colloidal solution in which solid particles suspend in a liquid phase. The sol formation involves hydrolysis of metal alkoxide ((MOR)_x_) in an aqueous medium. The subsequent condensation reaction results in the formation of an M-O-M network. Conventionally, metal alkoxides are used as the precursors for sol-gel process. However, the high cost of zinc alkoxides limits their use for preparing ZnO. So zinc salts such as zinc acetate dihydrate and zinc nitrate are used alternatively because of their ease of hydrolysis.

The sol-gel process is versatile, so nano-ZnO can also be prepared using a modified strategy, terming as ‘Pechini process’. This strategy relies on the formation of metallic complexes deriving from the reaction of metal precursor with hydrocarboxylic acid, followed by a condensation reaction with polyalcohol, e.g., ethylene glycol (EG). Hydroxycarboxylic acid such as citric, glycolic or lactic acts as the chelating agent [248]. Polyol plays the role of the solvent and stabilizer by bridging molecules for metal salt precursor in forming a gel [19,253]. The advantages of this approach include the steric entrapment of metal cation complexes and the prevention of segregation of nano-ZnO.

#### 3.2.4. Microemulsion Process

Microemulsions are thermodynamically stable colloidal system consisting of transparent and isotropic mixtures of water, oil and surfactant. Microemulsions are typically classified into water in oil (W/O) and oil in water (O/W), which are stabilized by surfactant molecules. So small droplets (micelles) of one phase are dispersed throughout the continuous phase. The droplets are considered as nanoreactors to carry out chemical reactions for forming crystal nuclei. The steric stabilization due to surfactant molecules on the micelles prevents the nanoparticles from the agglomeration. Water-in-oil microemulsions are also known as reverse micelles. The synthesis of ZnO NPs using reverse micelles involves the mixing of two types of microemulsions, i.e., one with zinc salt precursor and another one with the precipitating agent. So the micelles undergo extensive collision and coalescence events, causing the exchange of reactants and subsequent reaction to form nuclei in micelles.

Hingorani et al. first reported the synthesis of ZnO NPs (5–40 nm) from W/O microemulsions using zinc nitrate, ammonium carbonate (precipitating agent), CTAB (surfactant), 1-butanol (co-surfactant), and n-octane (oil phase). The first micelle solution comprised of zinc nitrate, n-octane and CTAB/1-butanol. The second micelle solution comprised of ammonium carbonate, n-octane and CTAB/1-butanol. After mixing the two micelle solutions, continuous collision and coalescence led to the precipitation of zinc carbonate and its subsequent transformation to ZnO NPs upon heating [254]. Since then, the synthesis of ZnO NPs using W/O emulsions had been carried out by the researchers using different organic compounds, surfactants and zinc salt precursors [255,256]. The drawbacks of microemulsion-based synthesis include the employment of a large amount of organic compound (oil phase) and the use of surfactant/cosurfactant for stabilizing the droplets.

### 3.3. Solid State Route

#### Mechanochemical Process

Mechanochemical process involves the milling of precursor powders in a ball mill for reducing their sizes to the nanoscale. The raw materials break-down into smaller particles and react chemically during milling or subsequent heat treatment to form a mixture phase of nanoparticles. A diluent is usually added to the starting materials during the milling process to separate the nanoparticles and prevent their subsequent growth. This is a solid state approach for fabricating ZnO NPs. Various types of grinding balls, often termed as the milling media, are used for milling raw powders. These include zirconia, stainless steel, and glass balls [257]. Generally, the size and shape of ground ZnO NPs depend greatly on the milling time of starting materials, calcination temperature and time [258,259,260]. So a longer milling time favors the formation of fined ZnO NPs. The drawbacks of this approach are time consuming, and the possible contamination of ground powders by milling medium.

Manzoor et al. employed mechanochemical method to fabricate ZnO NPs using anhydrous ZnCl_2_, anhydrous Na_2_CO_3_ and stainless steel balls in the presence of NaCl acting as a diluent [258]. The milled powders were then calcined at 250–400 °C for 30 min. Spherical ZnO NPs (19.4 nm) with a narrow size distribution were achieved by heating the milled powders at 250 °C. However, calcination at higher temperatures of 350 °C and 400°C led to the formation of larger ZnO NPs with mean sizes of 28.9 nm and 30.7 nm, respectively. The reactions during ball milling and calcination were given as follows,

Milling ZnCl_2_ + Na_2_CO_3_ + 6NaCl → ZnCO_3_ + 8NaCl (11)

Calcination ZnCO_3_ → ZnO + CO_2_ (12)

Very recently, Arsalani et al. ball-milled ZnCl_2_, KOH and MWCNTs to intiate the formation of ZnO nuclei on the tubes followed by hydrothermal treatment at 150 °C for 15 h [260]. During the milling process, high-energy collision resulted in the dissociation of KOH and ZnCl_2_. As such, Zn^2+^ ions reacted with hydroxyl ions from KOH through electrostatic interactions, and then deposited on MWCNTs to yield ZnO nuclei. Further hydrothermal treatment facilitated the growth of ZnO nuclei into ZnO NPs with an average diameter of about 50 nm on the nanotubes.

## 4. Antibacterial Performance

### 4.1. ZnO-Bacterial Interactions

**Figure 22:**
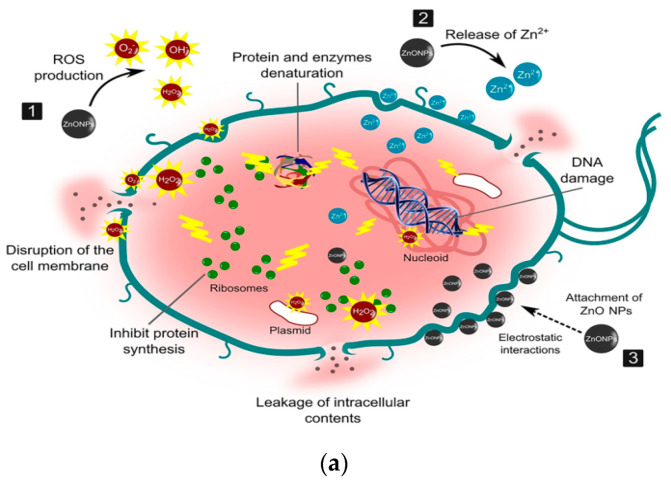

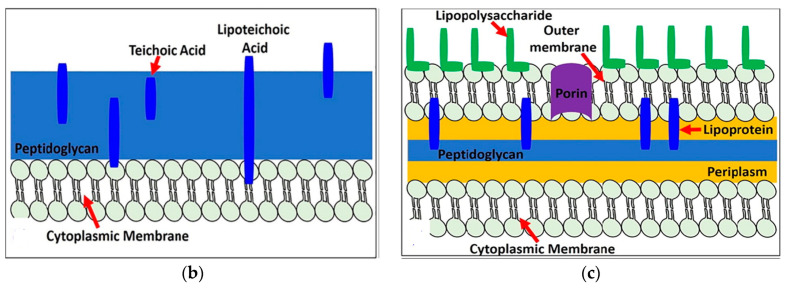
(**a**) Three possible mechanisms responsible for bactericidal activity of ZnO NPs: (1) The generation of reactive oxygen species (ROS), (2) release of Zn^2+^ ions, and (3) direct attachment on bacterial cell membrane. Reproduced from [261] under the terms of the Creative Commons Attribution 4.0 International License. Schematics showing the cell wall structures of (**b**) Gram-positive and (**c**) Gram-negative bacteria. Reproduced from [266] under the terms of the Creative Commons Attribution License.

ZnO NPs are well known to exhibit antibacterial activity against pathogens in the absence of photocatalytic activity. The exact bactericidal mechanism of ZnO NPs are not clearly known and still under debate. Several mechanisms have been proposed for bactericidal activities of ZnO NPs, including the direct contact with cell membrane, release of metallic ions, generation of ROS, and internalization of ZnO NPs (Figure 22a) [14,22,27,28,261,262]. For direct contact killing mechanism, ZnO NPs tend to disrupt the cell membrane function, and interfere electron transport chain upon attachment on the cell wall, leading to the the ROS production [263,264]. As recognized, pH at the point of zero charge (pzc) of ZnO NPs is 9.4, so producing a net positive surface charge on those nanoparticles in acidic, neutral and slightly alkaline solutions [265]. Accordingly, positively charged ZnO NPs can attach readily on the cell membranes of Gram-positive and Gram-negative bacteria due to the electrostatic interaction. This interaction disrupts the membrane structure and damages the cell integrity, leading to the leakage of intracellular contents [264]. In particular, ZnO NPs with very small sizes (ca ≤ 10 nm) can easily penetrate into cytoplasm, inducing the DNA damage and apoptosis. Generally, Gram-positive and Gram-negative bacteria have different sensitivity towards ZnO NPs due to the difference in their cell wall structures. Gram-positive bacteria have thick layers of peptidoglycan (20–80 nm), which are anchored to underlying cytoplasmic membrane via lipoteichoic acid (LTA). Likewise, wall bound teichoic acid is covalently attached to peptidoglycan via murein linkage units (Figure 22b) [266]. Peptidoglycan consists of repeated disaccharide units cross-linked by short peptides. In addition, peptidoglycan is relatively porous, and does not form a permeability barrier for small substrates. Together with teichoic acid and LTA containing phosphate groups, the cell wall of Gram-positive bacteria is a highly charged anionic polymer, thus favoring electrostatic attraction of positively charged nanoparticles [267]. In this respect, there is a greater affinity of ZnO to Gram-positive bacteria than Gram-negatives. On the contrary, peptidoglycan of Gram-negatives is thinner (<10 nm), and surrounded by an outer membrane containing lipopolysaccharides (LPS) (Figure 22c). LPS is a complex macromolecule, being impermeable to hydrophobic antibiotics. So outer membrane blocks the entry of numerous toxic compounds and prevents hydrophobic antibiotics from entering the organisms [268,269]. Therefore, Gram-negative bacteria with a thinner peptidoglycan layer and an outer membrane are more resistant to ZnO NPs than Gram-positives.

Joe et al. demonstrated that the antibacterial activity of ZnO NPs derives from an initial attachment of ZnO NPs on the bacterial cell walls, followed by release of Zn^2+^ ions and their subsequent penetration into the cytoplasm [22]. Once inside cytoplasm, Zn^2+^ ions interact with cellular organelles, thereby inhibiting the function of enzymes, deactivating proteins and DNA, and inducing oxidative stress [55]. Enzyme inhibition by zinc ions leads to an eventual cell death since enzymes are needed for performing essential metabolic pathways. Furthermore, Zn^2+^ ions also interact strongly with the protein-binding sites such as the sulfhydryl group of cysteine, thus inactivating the protein by forming Zn^2+^-cysteine complex [270]. Zn ion complex can also be formed by Zn^2+^ substitution into hydrogen bonds of deoxyribonucleic acid (DNA) base pairs, leading to the DNA damage and cell apoptosis [271]. Meanwhile, very fine ZnO NPs (ca ≤ 10 nm) can reach cytoplasm through the membrane disruption, resulting in enzyme inhibition as revealed by a marked suppression of the activity of β-galactosidase, a fundamental enzyme in bacterial metabolism [272].

The third possible mechanism for bactericidal activity of ZnO NPs is the ROS generation. Those reactive radicals interact with unsaturated fatty acids of phospholipid bilayer, leading to lipid peroxidation (LPO) that causes the loss of cellular functions [273]. As such, ROS induce oxidative stress and inactivate cellular surface proteins, resulting in a loss of membrane integrity and final cell death [274,275,276]. Thus ZnO NPs can induce ROS for killing bacteria even in the dark condition [262,277].

**Figure 23:**
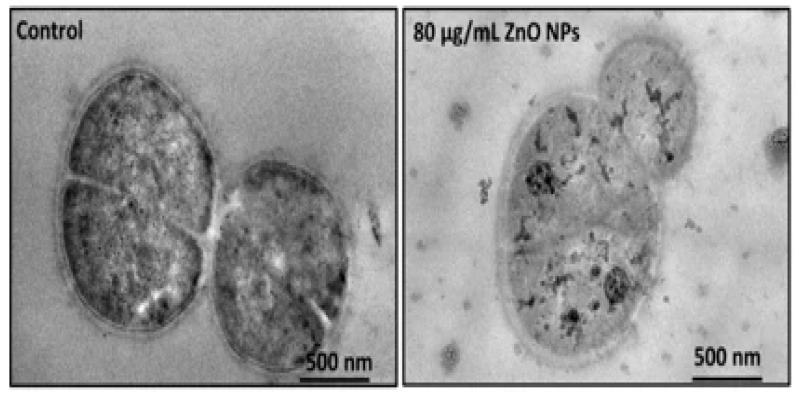
TEM images showing bare *D. radiodurans* cells (left panel), and internalization of ZnO NPs into bacterial cells (right panel). Reproduced from [276] under a Creative Commons Attribution 4.0 License.

**Figure 24:**
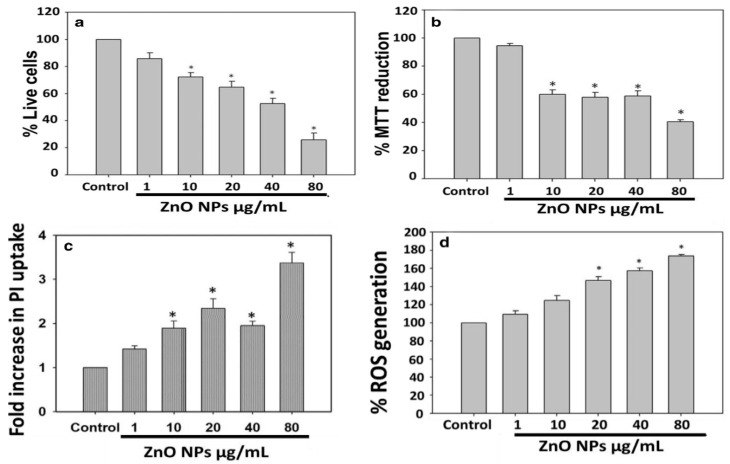
Reduction in viability of *D. radiodurans* upon exposure to ZnO NPs for 3 h as determined by: (**a**) Colony count method, (**b**) 3-(4,5-dimethylthiazol-2-yl)-2,5-diphenyltetrazolium bromide (MTT) assay, (**c**) propidium iodide (PI) uptake assay, and (**d**) ROS assay. The error bars are the standard deviation (*n* = 3); * denotes *p* < 0.05. Reproduced from [276] under a Creative Commons Attribution 4.0 International License.

Very recently, Singh et al. employed commercially available ZnO NPs for killing *Deinococcus radiodurans* with extraordinary radiation resistance [276]. TEM was employed to observe the internalization of ZnO NPs as shown in Figure 23. The antibacterial activity of ZnO NPs in vitro was determined from different bioassays, including conventional colony count method, 3-(4,5-dimethylthiazol-2-yl)-2,5-diphenyltetrazolium bromide (MTT) assay, and propidium iodide (PI) uptake assay. Colony forming units (CFU) were quantified in terms of the percentage of viable cells. PI staining protocol was employed for detection of bacterial cell death through the assessment of plasma membrane integrity using PI dye. Viable cells with intact membranes excluded PI, while the cells with a loss of membrane integrity facilitated uptake of PI. Colony count method indicated that ZnO NPs greatly reduced the viability of bacterial cells under a dose-dependent manner. At a dose of 80 μg/mL ZnO NPs, the viability reduced to ~25% (Figure 24a). A similar reduction trend in bacterial cell viability with increasing ZnO NPs concentrations was observed in the MTT assay (Figure 24b). Meanwhile, PI uptake assay showed an increase of PI uptake with increasing ZnO concentrations (Figure 24c). Thus ZnO NPs induced membrane damage by penetrating into cytoplasm, thereby facilitating the entry of PI dye inside the cells. The interaction of ZnO NPs with bacterial cell facilitates the ROS production in a concentration dependent manner (Figure 24d).

#### 4.1.1. Particle Size- and Dose-Dependence

The antimicrobial activity of ZnO NPs depends greatly on their sizes and concentrations. The smaller the nanoparticle size, the higher the activity is. Gram-positive *S. aureus* and Gram-negative *Escherichia coli* (*E. coli*) are typically selected as model bacteria strains for assessing antibacterial activity of ZnO NPs [261,271,275,276,277,278,279]. As previously mentioned, very fine ZnO NPs interact strongly with bacterial membrane, thus disrupting the cell wall function and producing high ROS level. In particular, small ZnO NPs with a large surface area attach on the cell walls, so releasing more Zn^2+^ ions and inducing higher ROS levels than large nanoparticles. Moreover, very fine nanoparticles can easily penetrate cell membranes into cytoplasm than larger particles. Raghupathi et al. reported that the antimicrobial activity of ZnO NPs (12–212 nm) against *S. aureus* is inversely proportional to their size. ZnO NPs with a size of 12 nm exhibited the highest bactericidal activity than those with sizes of 142 nm and 212 nm. The improved bactericidal effect resulted from the accumulation of very fine ZnO NPs on the outer cell membrane or in the cytoplasm, and the corresponding ROS generation [280]. Similarly, Mahamuni et al. also demonstrated that antibacterial and antibiofilm activities are inversely proportional to the size of ZnO NPs (~15−23 nm). ZnO NPs with the smallest size of ~15 nm exhibited excellent antibacterial and antibiofilm activities against Gram-positive *S. aureus* and Gram-negative *Proteus vulgaris* [255].

**Figure 25:**
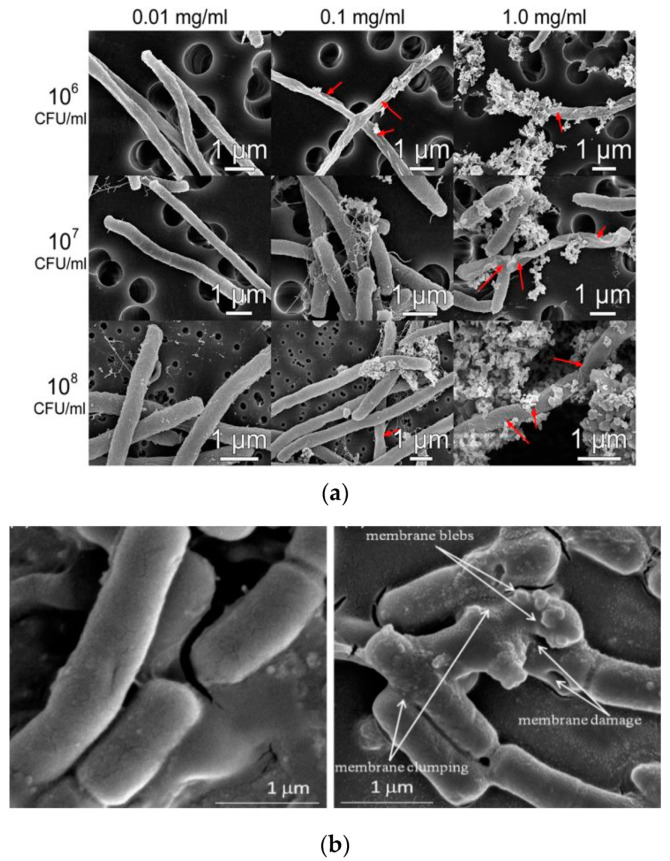

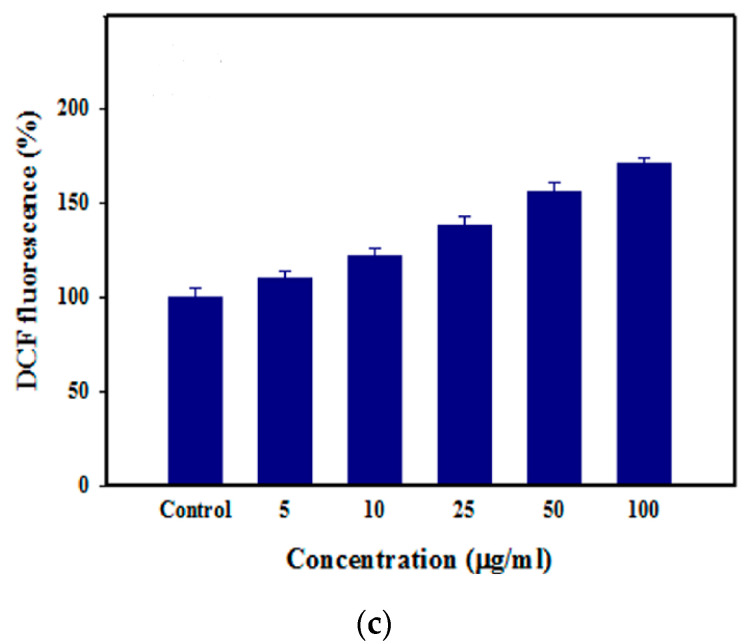
(**a**) SEM images of *E. coli* with concentrations of 10^6^, 10^7^ and 10^8^ CFU/mL upon exposure to ZnO nanoparticles of different doses (0.01, 0.1 and 1.0 mg/mL). Reproduced from [278] under a Creative Commons Attribution 4.0 International License. (**b**) SEM images of untreated *E. coli* cells (left panel) and ZnO NPs treated *E. coli* cells (right panel). Direct contact of ZnO NPs on bacterial membranes due to electrostatic interactions leads to membrane blebling, membrane damage, and membrane clumping as indicated by white arrows. Reproduced from [275] under a Creative Commons Attribution 4.0 International License. (**c**) Fluorescent 2,7-dichlorofluorescein (DCF) data showing the ROS level in *Pseudomonas aeruginosa* treated with ZnO NPs (5, 10, 25, 50 and 100 mg/mL) for 24 h. Reproduced from [29] under a Creative Commons Attribution License.

Leung et al. examined antimicrobial activity of commercial ZnO NPs with a mean size of 20 nm against *E. coli* using SEM imaging method [278]. Figure 25a shows that ZnO NPs interact with *E. coli* in a dose-dependent manner. At a low particle dose of 0.01 mg/mL, few ZnO NPs attach on the surface of *E. coli* as expected. By increasing nanoparticle dose to 1 mg/mL, many ZnO NPs adhere on *E. coli* due to electrostatic interactions, leading to the cell membrane damage in the form of holes as indicated by red arrows. The particle concentrations employed for SEM imaging are relatively high to facilitate the observation of ZnO-bacteria interactions. Recently, Arakha et al. demonstrated that direct contact of ZnO NPs with *E. coli* can result in the membrane blebbings, membrane damage, and membrane clumping leading to final cell death (Figure 25b) [275]. Dwivedi et al. investigated the effect of ZnO NPs (10 nm) concentrations on the ROS generation in *P. aeruginosa* (Figure 25c) [29]. Apparently, the ROS level increases with increasing ZnO NPs doses as expected. The dose-dependent effect of ZnO NPs on the ROS generation in *D. radiodurans* has been shown previously in Figure 24d.

#### 4.1.2. Minimal Inhibitory Concentration

The antimicrobial activities of ZnO nanostructures against various bacterial strains can be assessed by means of broth dilution and Kirby-Bauer disk diffusion techniques. The results are expressed in terms of minimum inhibitory concentration (MIC) and inhibition zone, respectively. More recently, da Silva et al. employed the sol-gel process using ethanolic zinc acetate solution and LiOH to synthesize ZnO NPs (5.3 nm); ZnO NPs were further heat treated at 600 °C to yield nanoparticles with sizes of 33.9 nm [26]. MIC values of the as-synthesized ZnO NPs and heat treated ZnO NPs against *S. aureus* were 78.2 and 208.2 µg/mL, while MIC values of the same samples against *E. coli* were 312.5 and 1250 µg/mL, respectively. Apparently, the as-synthesized ZnO NPs with a smaller size exhibited better bactericidal activity against both bacterial strains.

Generally, flavonoids and polyphenols extracted from natural plants are involved in the antibacterial activity of green ZnO nanostructures against various bacterial strains. Chen and coworkers synthesized ZnO NPs with a mean size of ~18 nm by reducing Zn(NO_3_)_2_·6H_2_O with *Geranium wallichianum* leaf extract. The bactericidal activity of spherical ZnO NPs against Gram positive *S. aureus*, *Bacillus subtilis*, Gram negative *E. coli*, *Pseudomonas aeruginosa*, and *Klebsiella pneumoniae* was investigated [31]. ZnO NPs were found to exhibit significant antimicrobial effect against those bacterial strains, especially Gram-positive bacteria. *B. subtilis* was the most vulnerable strain with an MIC value of 7.8 µg/mL, while *K. pneumoniae* was the least susceptible bacteria having MIC value of 125 µg/mL. The increased antibacterial efficacy of ZnO NPs was ascribed to the bioactive functional groups attached on the surface of nanoparticles.

Elumalai and Velmurugan biosynthesized ZnO NPs (mean size: ~16.66 nm) from *A. Indica* leaf extract, and investigated their antimicrobial activity against Gram-positive *S. aureus*, *B. subtilis*, Gram-negative *E. coli*, *P. aeruginosa*, and *Proteus mirabilis* [30]. Spherical ZnO NPs showed excellent antibacterial activity against those bacterial strains. Gram-positive bacteria, especially *S. aureus* is more sensitive to ZnO NPs. This can be ascribed to its cell wall structure containing porous and multilayered peptidoglycan as mentioned previously. The average values of zone of inhibition against *S. aureus* exposed to ZnO NPs with doses of 50 µg/mL and 200 µg/mL, are 14.4 ± 0.76 mm and 23 ± 0.50 mm, respectively. In contrast, the mean zones of inhibition against *E. coli* treated with ZnO NPs concentrations of 50 µg/mL and 200 µg/mL, are 12.6 ± 0.76 mm and 19.0 ± 0.50 mm, respectively. Moreover, MIC value of ZnO NPs against *S. aureus* and *B. subtilis* is 6.25 µg/mL, while a higher MIC value of 25 µg/mL is needed for killing Gram-negative *E. coli* and *P. aeruginosa.* So the inactivation of Gram-negative bacteria requires a higher concentration of ZnO NPs.

Abbasi et al. synthesized ZnO nanostructures from the callus extract of *Silybum marianum (L.) Gaern*. The as-synthesized ZnO exhibited a flower-like feature with a size of 44 nm, having MIC values of 33.33 and ≥100 μg/mL against *S. aureus* and *P. aeruginosa*, respectively [281]. Paduraru et al. prepared spherical ZnO NPs and flower-like ZnO using polyol process and microwave-assisted hydrothermal method. In polyol synthesis, zinc acetate was dissolved in ethylene glycol by refluxing at 160 °C to form a 1 M solution. The mixture was refluxed for further 12h at 160 °C, followed by centrifuging and drying in an oven to obtain spherical ZnO NPs with sizes of 14.63 ± 0.47 nm. The latter method involved microwave-assisted hydrothermal treatment of an aqueous solution of ZnSO_4_ and ammonia water at 120 °C for 10 min [27]. ZnO-nanoflowers were formed by sefl-assembly of ZnO nanorods (length: 10^3^ nm). The MIC values of ZnO-nanoflower against *E.coli* and *candida albicans* (fungus that causes human gut and skin diseases) were determined to be 1250 and 1250 μg/mL, respectively. However, MIC values against *E.coli* and *candida albicans* were largely reduced to 625 and 312 μg/mL respectively by treating with fined ZnO NPs (14.63 nm) prepared by polyol process. Their results were tabulated in Table 1.

Very recently, Liu et al. employed microwave-assisted solvothermal method to synthesize ZnO tapered rods with an average length of 1 µm using zinc acetate and ethylene glycol in the presence of CTAB. In addition, they also synthesized 7.7 mol% AgNPs/ZnO nanocomposite by dispersing ZnO tapered rods in an aqueous solution of AgNO_3_ and few drops of NaOH at 80 °C. The bactericidal efficacy of ZnO rods and AgNPs/ZnO nanocomposite against several bacterial strains was assessed accordingly [115]. In fact, AgNPs/ZnO nanocomposite exhibited a higher bactericidal activity than ZnO rods due to the presence of released silver ions from AgNPs and ROS generated from ZnO. As a result, AgNPs/ZnO nanocomposite had lower MIC values than ZnO rods as listed in Table 1.

Abinaya et al. prepared 5%Cu/ZnO prismatic nanoparticles (nanoplates) using zinc acetate, copper acetate monohydrate and NaOH at 160 °C for 5h by means of hydrothermal synthesis. They also hydrothermally synthesized 5%AgNPs/ZnO using zinc acetate, silver nitrate and NaOH at 160 °C for 5h [28]. In their study, 5%AgNPs/ZnO and 5%Cu/ZnO hybrids exhibited much lower MIC values than pure ZnO against *S. aureus* and *E. coli* (Table 1). The 5%AgNPs/ZnO and 5%Cu/ZnO hybrids would release silver and copper ions respectively for bactericidal activity. In particular, Ag^+^ ions from 5%AgNPs/ZnO hybrid were very effective for killing *E. coli* and *S. aureus* as evidenced by low MIC values of 38 and 40 µg/mL, respectively.

Zhong and Yun reported that GO/ZnO hybrid had very low MIC values toward several bacterial strains including *E. coli, Salmonella typhimurium*, *Enterococcus faecalis* and *B. subtilis*. In particular, GO/ZnO hybrid had the lowest MIC value of 6.25 µg/mL against both *E. coli* and *S. typhimurium* strains. In contrast, GO sheet and ZnO NPs exhibited a higher MIC value of 12.5 and 50 µg/mL respectively against *E. coli* and *S. typhimurium*. In other words, GO/ZnO hybrid caused more damage to *E. coli* and *S. typhimurium* than GO and ZnO NPs alone [240]. Excellent antibacterial activity of GO/ZnO hybrid was attributed to the synergistic effects of GO and ZnO NPs. Generally, graphene sheet showed three modes of bactericidal activity, i.e., membrane lipid peroxidation, nano- knife and wrapping effects. Graphene sheets were capable of oxidizing and extracting unsaturated lipids from bacterial membranes, leading to LPO. The sharp edges of graphene sheets acted as a nano-knife to cut and damage bacterial membranes. Furthermore, graphene sheets with a lateral dimension of several micrometers served as a blanket to wrap bacteria completely, thus isolating bacterial strains from external environment and limiting their access to nutrients [13]. In this respect, finely dispersed ZnO NPs on GO inactivated those bacterial strains through the release of Zn^2+^ ions and the production of ROS [240]. As such, GO sheet caused LPO while ZnO NPs induced ROS, resulting in the GO/ZnO hybrid with superior antibacterial activity (Table 1). Table 1 also summarized the MIC values of ZnO nanostructures prepared by different processes against various bacterial strains.

#### 4.1.3. Zone of Inhibition

**Figure 26:**
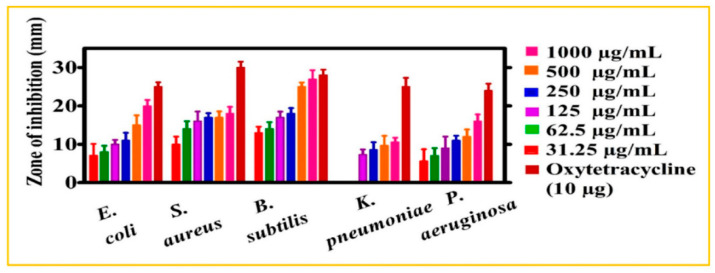
Growth inhibition zones for various bacterial strains exposed to green ZnO NPs of different concentrations. Oxytetracycline was used as a positive control. Reproduced from [31] under a Creative Commons Attribution License.

As mentioned, Chen and coworkers treated Gram positive (*S. aureus, B. subtilis*), Gram negative (*E. coli, P. aeruginosa,* and *K. pneumoniae*) strains with green ZnO NPs (~18 nm). *B. subtilis* was the most susceptible bacteria with an MIC value of 7.8 µg/mL, while *K. pneumoniae* was the least susceptible strain having an MIC value of 125 µg/mL [31]. Figure 26 showed the growth inhibition zones for those bacterial strains were dependent on ZnO NPs concentrations. Gram-positive *S. aureus* and *B. subtilis* exhibited larger values of growth inhibition zones than Gram-negative bacterial strains. At ZnO NPs doses of 500 and 1000 µg/mL, the greatest values of the growth inhibition zones were observed in *B. subtilis*, so ZnO NPs being the most efficient materials against this bacterial strain.

**Figure 27:**
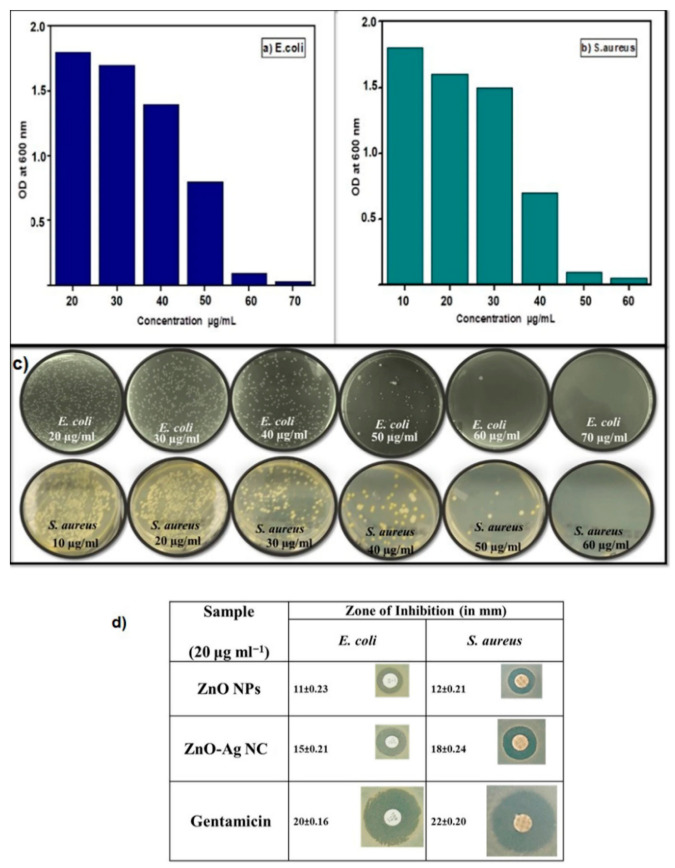
Effect of AgNPs/ZnO concentrations on the viability of (**a**) *E. coli*, (**b**) *S. aureus*, and (**c**) bacterial colony features cultured on agar plates. (**d**) Inhibition zone of AgNPs/ZnO nanocomposite (NC), ZnO NPs and Gentamicin. Gentamicin is an antibiotic specifically used for treating bacterial infections. OD in (**a**) denotes optical density. Reproduced from [282] under a Creative Commons Attribution 4.0 International License.

Zare et al. biosynthesized AgNPs/ZnO nanohybrid hydrothermally using *Thymus vulgaris* leaf extract, zinc nitrate, silver nitrate and sodium hydroxide reagents. The hydroxyl groups in flavonoids and phenols of leaf extract were effective to reduce zinc ions into ZnO NPs [282]. The antimicrobial activity of AgNPs/ZnO hybrid against *E. coli* and *S. aureus* was dose dependent (Figure 27a,b). The bacterial viability decreased with increasing nanohybrid doses, especially for *S. aureus*. At a dose of 50 µg/mL, the viability of *S. aureus* was considerably lower than that of *E*. *coli*. The features of bacterial colonies on agar plates, and the zone of inhibition determined from the disk diffusion assay were shown in Figure 27c,d, respectively. For a given bacterial strain, the diameter of zone of inhibition (ZOI) of AgNPs/ZnO nanohybrid was larger than that of pure ZnO NPs. In this respect, doped ZnO hybrid showed a higher antimicrobial activity than undoped ZnO. Furthermore, AgNPs/ZnO hybrid exhibited a stronger bactericidal effect against *S. aureus* compared to *E. coli*. The ZOI values of AgNPs/ZnO hybrid against *S. aureus* and *E. coli* were 18 ± 0.24 and 15 ± 0.21 mm, respectively. From the literature, susceptible organisms displayed relatively larger ZOI, while resistant bacterial strains had smaller or no ZOI upon exposure to nanomaterials [23]. Apparently, released Ag^+^ ions from AgNPs and Zn^2+^ ions from ZnO of AgNPs/ZnO hybrid in the culture medium were responsible for bactericidal activity. Gram-negative *E*. *coli* was less susceptible to AgNPs/ZnO than *S. aureus* owing to impermeable outer membrane consisting of LPS. Very recently, Chauhan et al. prepared green AgNPs/ZnO nanoparticles (38 nm) using Cannabis sativa leaf extract and zinc acetate. The composite nanoparticles also exhibited excellent bactericidal activity against MRSA and *S. aureus*. From the agar diffusion test results, the diameters of zone of inhibition for MRSA (16 ± 0.404 mm) and *S. aureus* (16 ± 0.818 mm) were larger than those of *E. coli* (14 ± 0.568 mm), *P. aeruginosa* (13 ± 0.458 mm) and *K. pneumoniae* (14 ± 0.36 mm). In another study, this same group also demonstrated that Gram-negative *E. coli*, *P. aeruginosa* and *K. pneumoniae* were more resistant to green ZnO nanoflowers (54.1 nm) than Gram-positive MRSA and *S. aureus* [283]. In the case of transition metal doped ZnO NPs, the Cr dopant also improved antibacterial activity of ZnO NPs against *K. pneumoniae* and *P. aeruginosa* on the basis of ZOI results [104].

#### 4.1.4. Bactericidal Efficacy

**Figure 28:**
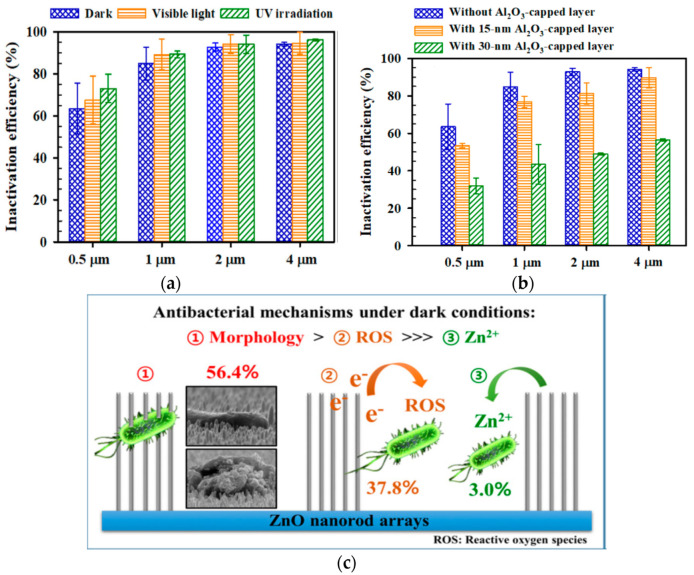
(**a**) Inactivation efficiency of *E. coli* treated with ZnO nanorod arrays of various lengths (0.5–4 μm) for 30 min under different conditions (dark, visible light, and UV irradiation). (**b**) Inactivation efficiency of *E. coli* treated with ZnO nanorod arrays of various lengths with different Al_2_O_3_ layer thicknesses for 30 min in the dark. The values are the average of triplicate measurements, and error bars denote the standard deviation. (**c**) Bactericidal mechanisms of ZnO nanorod arrays in the dark: membrane rupture due to the nanorod piercing (inset: SEM images) > ROS generation > released Zn^2+^ ions. Reproduced from [288] with permission of Elsevier.

One-dimensional ZnO nanorods/nanowires show potential applications as a bioimaging material, theranostic agent, antibacterial agent and biosensor in biomedical sector [284,285,286,287]. Jeong et al. hydrothermally synthesized ZnO nanorod arrays of different lengths ranging from 0.5 to 4 μm using ZnO seeded silicon wafers [288]. They reported that the array with long nanorods (4 μm) exhibit excellent inactivation efficiency against *E. coli* up to 94.2% in the dark for 30 min (Figure 28a). In contrast, short nanorods (0.5 μm) exhibit lower inactivation efficiency of 63.5%. The bactericidal potency of ZnO nanorods is attributed mainly to their mechanical piercing effect. ZnO nanorods attached to bacterial cell wall can pierce through the membrane completely, leading to the membrane rupture, and releasing intracellular contents; the direct contact killing is revealed by the SEM images. Meanwhile, ZnO nanorods also release zinc ions upon attachment on bacterial cell wall, and generate ROS for antibacterial activity. As mentioned, oxygen vacancies form easily in ZnO nanorods [20,21,289,290,291]. Those vacancies act as electron donors for the conduction band of ZnO, thus facilitating the ROS generation [277]. The ROS level can be further increased by irradiating with UV light. As such, ZnO nanorods absorb UV radiation to create reactive radicals as a result of electron-hole pair generation. The bactericidal efficiency of short nanorods (0.5 μm) reaches 73.1%, while that of long nanorods (4 μm) approaches 96.2% under UV irradiation (Figure 28a). When the nanorod arrays are coated with Al_2_O_3_ layer of different thicknesses, the inactivation efficiency is markedly decreased, especially for those with a thicker alumina layer. Thus alumina layer can suppress both the ROS generation and Zn^2+^ ion release effectively (Figure 28b). From these results, antibacterial efficacy of ZnO nanorod arrays in the dark condition derives mainly from the membrane rupture effect, followed by the ROS generation and released Zn^2+^ ions from nanorods. In other words, antibacterial activity of ZnO nanorod array is mainly due to the attachment of ZnO array to bacterial cell wall, leading to the membrane piercing effect and the release of Zn^2+^ ions as shown in Figure 28c.

**Figure 29:**
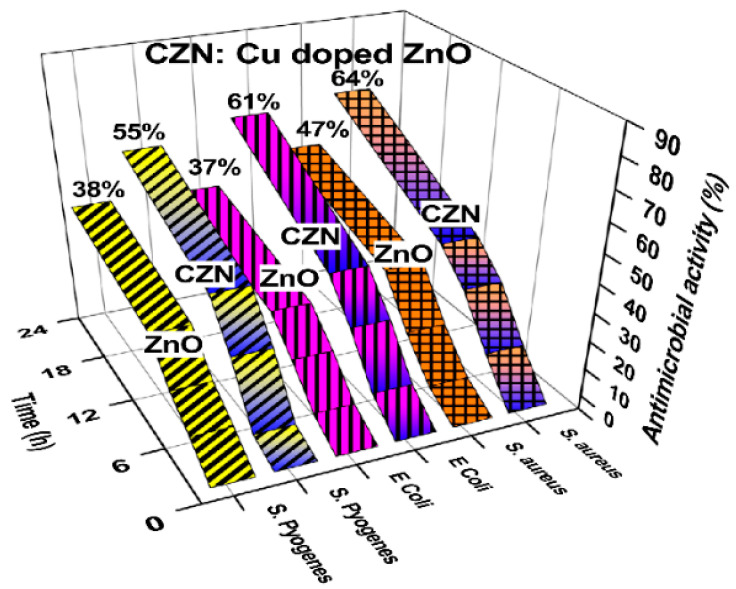
Antibacterial efficacy of pristine ZnO and Cu-doped ZnO nanorods using shake flask method. Reproduced from [103] with permission of Springer.

Bhuyan et al. studied antibacterial activity of pristine ZnO and Cu-doped ZnO nanorods (CZN) prepared by mechanochemical process against *E. coli* by means of shake flask method [103]. In the process, those samples were dispersed in bacterial suspensions and placed in an incubator shaker at 37 °C for different time periods. The results are depicted in Figure 29. Apparently, Cu-doped ZnO nanorods exhibit higher antimicrobial activity than pristine ZnO nanorods against *S. aureus*, *Streptococcus pyogenes* and *E. coli.* The improved bactericidal effect derives from the release of Cu^2+^ ions from Cu-doped ZnO. The killing effect of Cu-doped ZnO is more effective against *S. aureus* than *E. coli*. In addition, Gram-positive *S*. *pyogenes* appears to be more resistant to the attack of doped ZnO. *S*. *pyogenes* is a pathogen responsible for pharyngitis, impetigo, rheumatic fever, and acute glomerulonephritis [292].The cell wall structure of *S*. *pyogenes* consists of complex polysaccharides and proteins, i.e., peptidoglycan layer is overlaid with M protein and an outer hyaluronic acid capsule. The capsule is more resistant to the attack of ROS than peptidoglycan [293].

**Figure 30:**
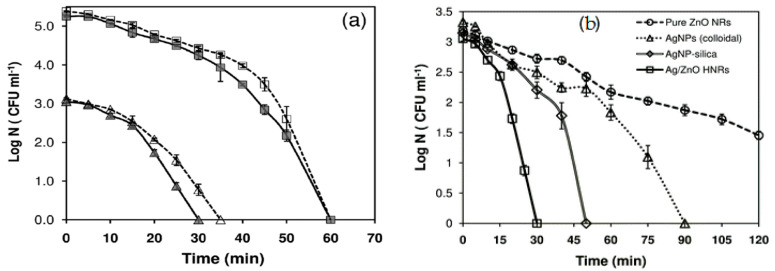
(**a**) The growth of bacterial populations as expressed by the logarithm of the number of cells (N) versus time. The unit of N is given as the colony forming unit (CFU) per mL. Bactericidal activity of AgNPs/ZnO nanorods against *E. coli* tested at an initial bacterial concentration of 10^3^ CFU/mL (triangle) and 10^5^ CFU/mL (square) in both deionized water (continuous line) and phosphate buffer medium (dashed line), respectively. (**b**) Bactericidal activity of AgNPs/ZnO nanorods, pure ZnO nanorods, colloidal AgNPs and AgNPs/silica against E. coli tested at an initial bacterial concentration of 10^3^ CFU/mL. Four independent tests were performed against E. coli, and the average values reported. Reproduced from [235] under a Creative Commons Attribution 3.0 Unported License.

**Figure 31:**
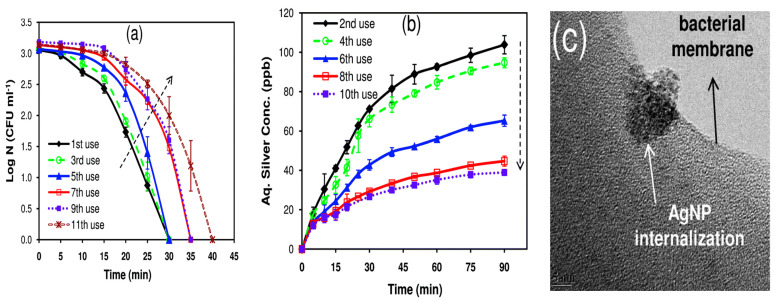
(**a**) Disinfection performance of AgNPs/ZnO nanorods after reusing for 11 runs against *E. coli* (10^3^ CFU/mL). (**b**) The corresponding released silver ions vs time profiles after every usage. (**c**) TEM image showing internalization of AgNP by *E. coli*. Reproduced from [235] under a Creative Commons Attribution 3.0 Unported License.

Agnihotri et al. studied bactericidal efficacy of AgNPs/ZnO nanorods against *E. coli* of different populations [235]. The time needed for full inactivation of *E. coli* at concentrations of 10^3^ and 10^5^ CFU/mL was determined (Figure 30a). The time for complete bacterial inactivation was relatively shorter (i.e., 30 min) by treating *E. coli* (10^3^ CFU/mL) with AgNPs/ZnO nanorods. Furthermore, AgNPs/ZnO nanorods also exhibited better bactericidal performance when compared to pure ZnO nanorods, colloidal AgNPs, and AgNPs/silica samples (Figure 30b). In addition, antibacterial activity and released Ag^+^ ions from the AgNPs/ZnO nanorods reused for several runs were also evaluated. The full bacterial inactivation time increased with increasing reused test runs due to a decline in silver ions released from the AgNPs/ZnO nanorods (Figure 31a,b). The AgNPs were found to translocate across the cell membrane under TEM examination (Figure 31c). Internalization of AgNPs facilitated the generation of ROS, dysfunction of cell respiration, and disruption of DNA replication of *E. coli*. Overall, they attributed good antimicrobial efficacy of AgNPs/ZnO nanorods to the contact killing and released Ag^+^ ions since Zn^2+^ ions were not detected in treated bacterial cells.

As mentioned, graphene oxide sheet with a lateral dimension of several micrometers serves as a template for anchoring ZnO NPs on its surface to form GO/ZnO nanohybrid (Figure 23). Thus ZnO NPs of nanohybrid inactivate pathogens through direct contact killing effect by releasing zinc ions and generating ROS [240]. GO also contributes to bactericidal activity by inducing lipid peroxidation [13]. Moreover, GO sheet with a large lateral size can wrap bacteria completely, thereby cutting off the supply of nutrients from the growth environment [13,294]. Accordingly, GO/ZnO hybrid exhibits low MIC values against several bacterial strains, i.e., *E. coli*, *S. typhimurium* and *B. subtilis* as listed in Table 1.

**Figure 32:**
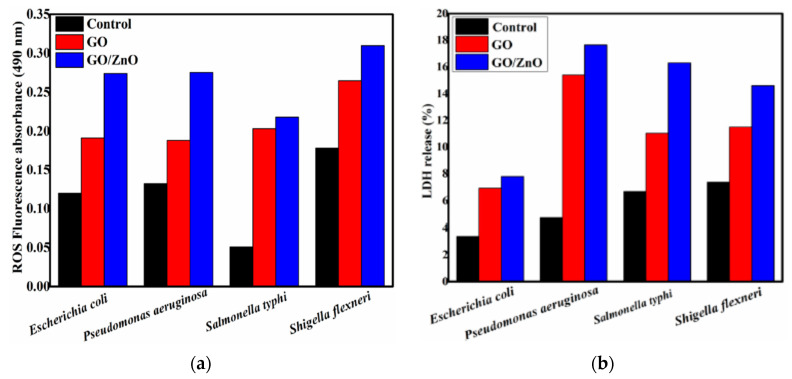
(**a**) ROS and (**b**) lactate dehydrogenase (LDH) levels of different Gram-negative bacteria strains treated with pure GO and GO/ZnO nanohybrid. Reproduced from [246] with permission of Springer Nature.

In a recent study of Prema et al., ZnO NPs prepared by co-precipitation process are added to GO solution to form GO/ZnO nanohybrid [246]. Figure 32a shows the ROS level in *E. coli*, *P. aeruginosa*, *S. tryphi*, and *Shigella flexneri* treated with pristine GO and GO/ZnO nanohybrid. Pristine GO induces a moderate ROS level in those Gram-negative bacteria. Meanwhile, ZnO NPs of nanohybrid release Zn^2+^ ions when they come in contact with all bacterial strains. Therefore, GO/ZnO hybrid exhibits a higher ROS level than pure GO as expected. The induction of ROS in Gram-negatives leads to the membrane disruption, resulting in the leakage of cytoplasmic enzymes as revealed by lactate dehydrogenase (LDH) assay (Figure 32b). LDH is a biomarker of damaged cell membrane integrity showing the percentage level of enzyme leakage from the cytoplasm.

**Figure 33:**
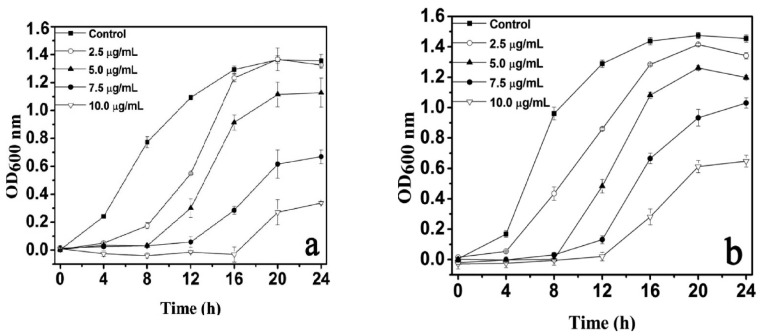

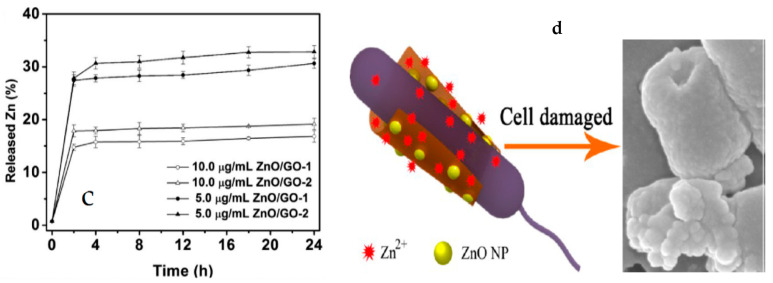
Growth curves of *E. coli* treated with (**a**) GO-1/ZnO and (**b**) GO-2/ZnO hybrids of different doses; (**c**) Zinc released from GO/ZnO composites; (**d**) Wrapping of graphene sheet around *E. coli* and released Zn^2+^ ions led to bacterial cell membrane damage. The experiments were performed in triplicates, and the results were given as mean ± standard deviation. Reproduced from [242] with permission of the American Chemical Society.

Recently, Wang et al. synthesized GO/ZnO nanohybrids solvothermally with different GO/ZnO mass ratios of 1:3 (denoting as GO-1/ZnO) and 1:2 (denoting as GO-2/ZnO). In nanohybrids, ZnO NPs with a size of about 4 nm were homogeneously anchored onto GO sheets [242]. Figure 33a,b show the growth curves over time of *E. coli* treated with both nanohybrids of different concentrations. The curves are obtained by monitoring the changes in optical density (OD) at 600 nm. Typical sigmoidal curves with three different stages (phases), i.e., a lag phase, an exponential (logarithmic) phase, and a plateau phase, can be readily seen for the growth of bacterial populations. At a low dose of 2.5 μg/mL, the lag phase is relatively short (4 h), after which *E. coli* populations grow rapidly with time in the exponential phase by treating with GO-1/ZnO and GO-2/ZnO hybrids. Both hybrids can suppress microbial growth more effectively by increasing the doses, especially at a high concentration of 10.0 μg/mL. The lag phase time is delayed by 16 h and 12 h, respectively at a dose of 10.0 μg/mL, which is followed by the exponential stage and final plateau phase with low bacterial popupations. Meanwhile, the close contact of homogeneously dispersed ZnO NPs with *E. coli* favors the release of zinc ions from nanohybrids (Figure 33c). The bactericidal efficacy derives from the synergistic effect of released zinc ions from the ZnO/GO nanohybrids, and the wrapping of graphene sheet around *E. coli* (Figure 33d).

### 4.2. Bactericidal Activity under Visible Light

#### 4.2.1. Metal Doping

**Figure 34:**
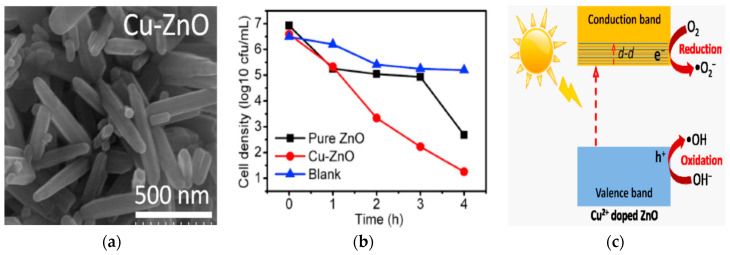
(**a**) SEM image showing morphology of Cu-doped ZnO nanorods; (**b**) Photocatalytic efficiency of ZnO NPs and Cu/ZnO nanorods against *E. coli* under simulated solar light irradiation; (**c**) Photocatalytic mechanism of Cu/ZnO nanorods under solar light. Reproduced from [298] with permission from Elsevier.

The bactericidal performance of ZnO nanostructures can be further enhanced by irradiating with electromagnetic waves. In general, photocatalytic ROS generation is mainly responsible for bactericidal activity of ZnO nanostructures with a wide bandgap under UV irradiation [295]. The bandgap of ZnO nanostructures can be greatly reduced by doping with transition metals. As such, ZnO can absorb photons of longer wavelength, thereby shifting its optical absorption edge from UV to visible region. Therefore, bactericidal activity of nano-ZnO under visible light can be improved by doping with metals [296,297,298,299]. More recently, Qi et al. synthesized pure ZnO NPs, and Cu-doped ZnO nanorods using co-precipitation process (Figure 34a) [298]. The antibacterial activity of nano-ZnO under simulated solar light was investigated. The bandgap of ZnO NPs was 3.20 eV, and reduced to 2.81 eV by doping with Cu. The Cu/ZnO nanorods exhibited better antibacterial activity than ZnO NPs under simulated sunlight illumination (Figure 34b). This was attributed to the generation of ROS on Cu/ZnO nanorods under sunlight. Copper dopant improved photocatalytic activity of ZnO by introducing midgap states below the CB of ZnO (Figure 34c). In this respect, photogenerated electrons were readily excited from the VB of ZnO to localized Cu midgap states, leading to the formation of superoxide anion and hydroxyl radicals for bactericidal activity. From Figure 34b, pure ZnO NPs displayed a sharp increase in bactericidal activity under solar light illumination for 4h. This was presumably caused by the presence of oxygen vacancies in ZnO NPs, thereby reducing the band-gap and enhancing solar light photocatalytic effect.

**Figure 35:**
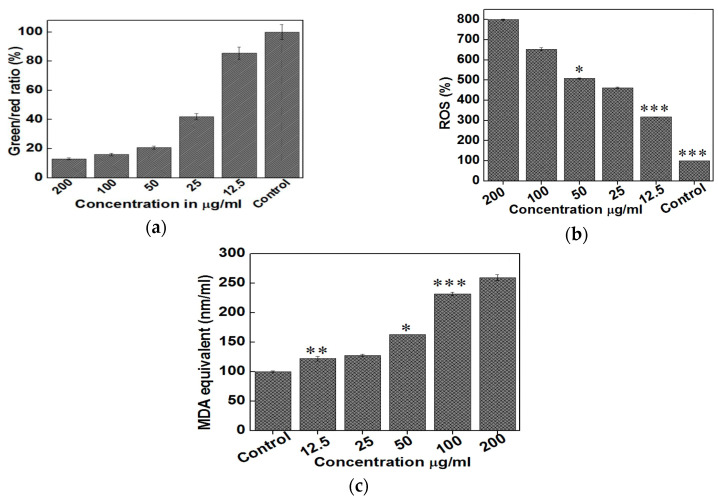
(**a**) Change in fluorescence intensity of green/red ratio of live/dead assay, (**b**) ROS and (**c**) MDA levels of *E.coli* treated with different Cu_5_/ZnO concentrations. The data were expressed as the mean ± SD (standard deviation) for three independent experiments (*n* = 3). *p* < 0.05 (*), 0.001 (**) and 0.0001 (***) were measured significant as compared to control. Reproduced from [102] with permission of the American Chemical Society.

**Figure 36:**
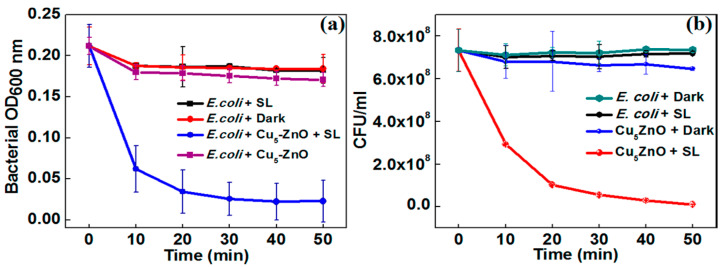
Photoinactivation of *E. coli* treated with Cu5/ZnO (200 µg/mL) under solar light (SL). The plots of (**a**) OD600 and (**b**) CFU/mL as a function of time. The experiments were performed in triplicates and data were expressed as the mean ± SD. Reproduced from [102] with permission of the American Chemical Society.

Gupta and Bahadur synthesized Cu-doped ZnO nanocomposites through hydrolysis and condensation of zinc acetate and copper acetate in a polyol medium, i.e., diethylene glycol (DEG) at 170−180 °C for 1h. Based on the nominal concentration of Cu, the resulting nanocomposites were designated as Cu_5_/ZnO (5% Cu) and Cu_10_/ZnO, respectively [102]. Figure 35a,c summarize the live/dead staining, ROS and malonaldehyde (MDA) results of *E. coli* treated with different Cu_5_/ZnO concentrations. A marked decline in green fluorescence at Cu_5_/ZnO concentrations ≥100 µg/mL demonstrates the excellent efficacy against *E. coli.* As recognized, a mixture of SYTO9 and propidium iodide (PI) fluorescent dyes is used in the live/dead assay to distinguish viable bacterial cells with intact plasma membranes from dead cells with damaged membranes. SYTO9 dye can penetrate cell membranes of both viable and dead cells, giving rise to green fluorescence. However, PI only penetrates cell membranes of dead cells, producing red fluorescence. Moreover, Cu_5_/ZnO also induces ROS and membrane lipid peroxidation in *E. coli*, resulting in the loss of membrane integrity (Figure 35b,c). Both ROS and MDA levels increase markedly as the Cu_5_/ZnO content increases. MDA is a biomarker for revealing lipid membrane peroxidation. Figure 36a,b show the effect of solar light irradiation on bactericidal potency of Cu_5_/ZnO (200 µg/mL) against *E. coli* as determined by OD600 and colony counting methods. A large amount of *E. coli* is inactivated due to the creation of ROS under solar light irradiation. From Figure 36b, inactivation of E. coli by Cu_5_/ZnO to a small extent in the dark can be readily seen. As mentioned, nano-ZnO possesses intrinsic oxygen vacancies that facilitate the formation of superoxide anion, hydroxyl radicals, singlet oxygen and H_2_O_2_ in the dark [20,21,300,301,302]. The amount of oxygen vacancies in ZnO NPs can be somewhat increased by doping with Cu [303]. Thus oxygen vacancies, released Cu, and Zn ions from Cu_5_/ZnO in the culture medium [22], are contributed to bactericidal activity to a certain degree in the dark. In another study, Gupta et al. also reported that Ag_5_/ZnO nanospheres exhibit superior bactericidal effect against *E. coli* under solar light [304]. The photocatalytic bactericidal effect derives from the creation of superoxide anion and hydroxyl radicals due to the surface plasmon effect of AgNPs under solar light as shown in Figure 5A. Both reactive species can damage bacterial cell membrane leading to cell death after 30 min of solar light illumination.

**Figure 37:**
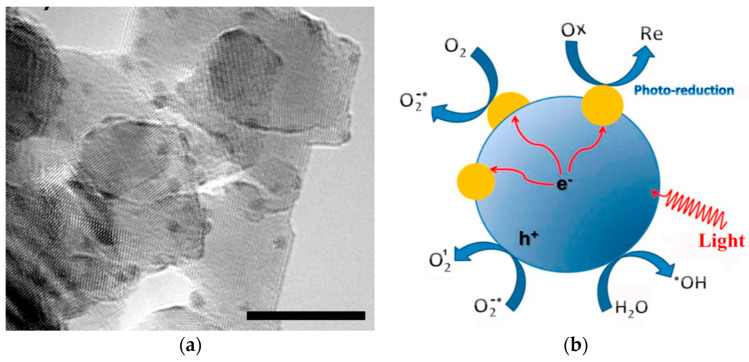

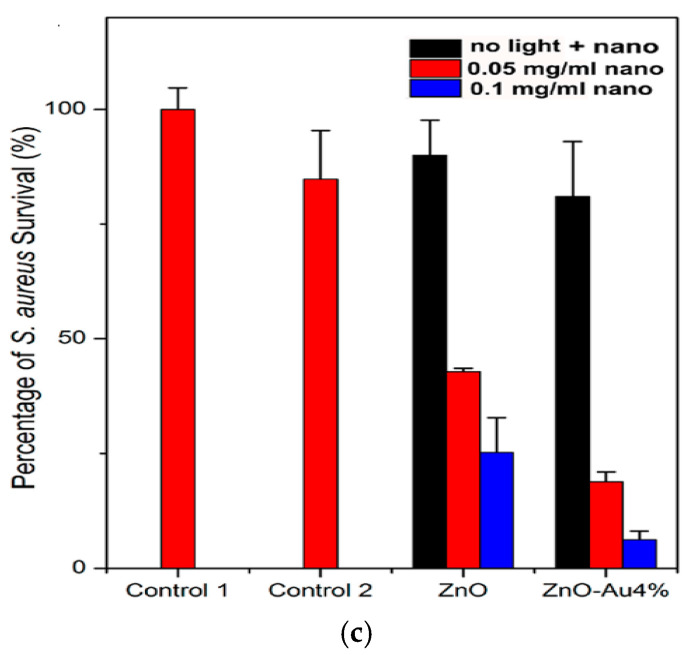
(**a**) TEM image and (**b**) schematic illustration of the production of hydroxyl, superoxide anion and singlet oxygen radicals on 4% AuNPs/ZnO exposed to simulated sunlight. Fine grey circles in (**a**) are AuNPs; scale bar is 20 nm. (**c**) A bar graph showing *staphylococcus aureus* survival upon exposure to ZnO NPs and 4% AuNPs/AgNPs at doses of 0.05 and 0.1 mg/mL without (black column) and with simulated sunlight illumination for 10 min. Control 1 is bacteria exposed to neither NPs nor light. Control 2 denotes bacteria exposed to simulated sunlight for 10 min in the absence of NPs. All tests are conducted in triplicates and repeated at least twice to obtain reproducibility. Reproduced from [305] with permission of the American Chemical Society.

For AuNPs/ZnO nanocomposites, hot electrons are injected from noble AuNPs into the CB of ZnO for reacting with adsorbed oxygen molecules to create ROS under visible light irradiation due to plasmonic oscillation. He et al. deposited 2–20% AuNPs (1–3 nm) onto ZnO NPs (30–40 nm), and reported a dramatic increase in the production of hydroxyl, superoxide and singlet oxygen radicals on the nanocomposites under simulated sunlight irradiation (Figure 37a,b) [305]. As a result, AuNPs/ZnO nanocomposite with a dose of 0.05 mg/mL exhibited good antibacterial activity against *S. aureus* under simulated sunlight exposure for 10 min. At a dose of 0.1 mg/mL AuNPs/ZnO, the percentage of survival of *S. aureus* decreased significantly due to the ROS generation (Figure 37c).

**Figure 38:**
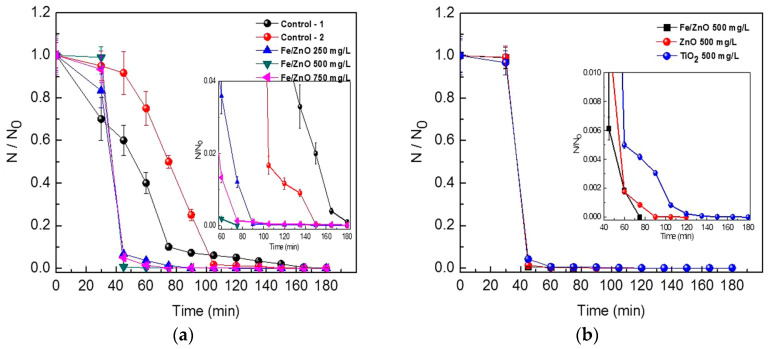
(**a**) Effect of Fe/ZnO NPs concentrations on solar-photocatalytic disinfection (PCD) kinetics of multidrug-resistant (MDR) *E. coli*. Control-1: bacteria exposed to Fe/ZnO NPs in the dark; Control-2: bacteria without Fe/ZnO NPs under solar irradiation. (**b**) Effect of different catalysts on the solar-PCD kinetics of *MDR E. coli* at a catalyst concentration of 500 mg/L. Initial MDR *E. coli* concentration = 1.2 × 10^7^ CFU/mL, temperature = 35 ± 2 °C, pH = 6.5. Error bars indicate standard deviation of replicates (*n* = 3). Reproduced from [306] under a Creative Commons Attribution 4.0 International License.

Das et al. investigated solar-photocatalytic disinfection (PCD) of spherical Fe-doped ZnO NPs against MDR *E. coli* isolated from wastewater of a rural healthcare center [306]. Fe-doped ZnO NPs with sizes of 80–100 nm were synthesized by chemical precipitation process. MDR *E. coli* was the main bacteria causing urinary tract infections and water borne diseases. Figure 38a shows the time dependent disinfection of MDR *E. coli* as a function of Fe/ZnO NPs doses under solar light. Apparently, bactericidal efficiency increases with increasing Fe/ZnO NPs dose up to 750 µg/mL; (Note that mg/L = µg/mL). At an optimal dose of 500 µg/mL, the time for complete disinfection for Fe/ZnO NPs was relatively shorter (i.e., 80 min) when compared to undoped ZnO (120 min) and TiO_2_ (180 min) as shown in Figure 38b (inset). By doping with Fe, Fe^3+^ ions replaced Zn^2+^ ions at tetrahedral sites of ZnO [307]. Under solar light irradiation, Fe^3+^ ions acted as the hole and electron traps, thus promoting the ROS generation. The photocatalytic reactions were given by [308],

Fe^3+^ + e^−^ → Fe^2+^ (electron trap) (13)

Fe^2+^ + O_2(ads)_ → Fe^3+^ + •O_2_^−^ (14)

Fe^3+^ + h^+^ → Fe^4+^ (hole trap) (15)

Fe^4+^ + OH^−^_(ads)_ → Fe^3+^ + •OH (16)

**Figure 39:**
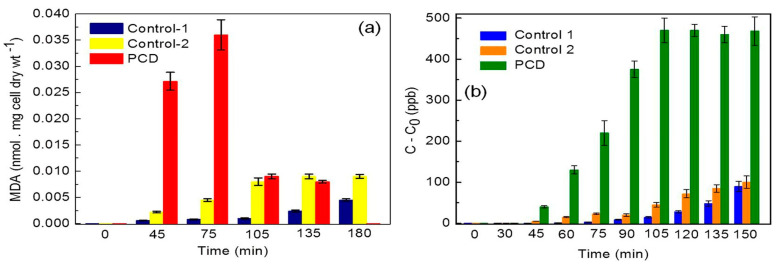
Plots of (**a**) malondialdehyde (MDA) content vs time and (**b**) leakage of K^+^ ion vs time for MDR *E. coli* treated with Fe/ZnO NPs. Initial MDR *E. coli* concentration = 1.2 × 10^7^ CFU/mL, Temperature = 35 ± 2 °C, pH = 6.5, [Fe/ZnO NPs] = 500 mg/L. Error bars indicate standard deviation of replicates (*n* = 3). Reproduced from [306] under a Creative Commons Attribution 4.0 International License.

**Figure 40:**
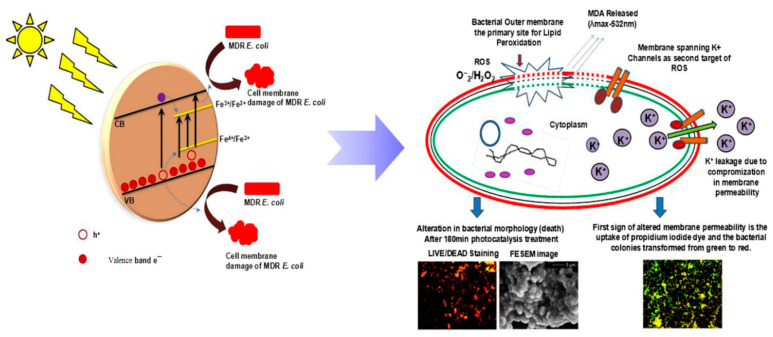
Schematic view of visible-light photocatalytic bactericidal activity of Fe/ZnO NPs against *MDR E. coli*. Reproduced from [306] under a Creative Commons Attribution 4.0 International License.

The decline in bacterial populations due to Fe/ZnO NPs was attributed to the generation of superoxide and hydroxyl radicals, leading to membrane lipid peroxidation as revealed by the MDA results (Figure 39a), and the leakage of of K^+^ ion from MDR *E. coli* (Figure 39b). Figure 40 is a schematic showing solar-photocatalytic disinfection of Fe/ZnO NPs against MDR *E. coli* (left panel), leading to the membrane damage due to lipid peroxidation and the leakage of K^+^ ions from cytoplasm (right panel).

#### 4.2.2. Non-Metal Doping

**Figure 41:**
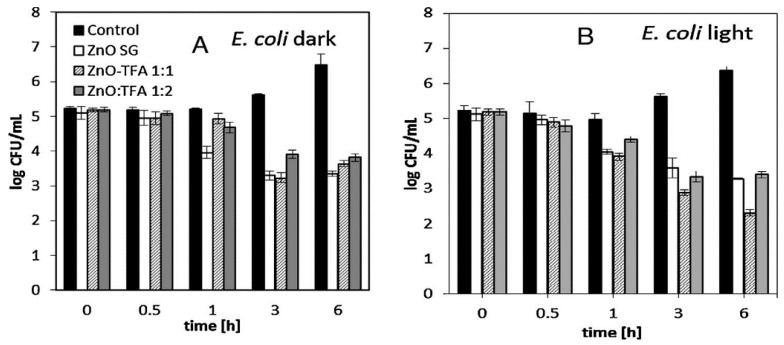

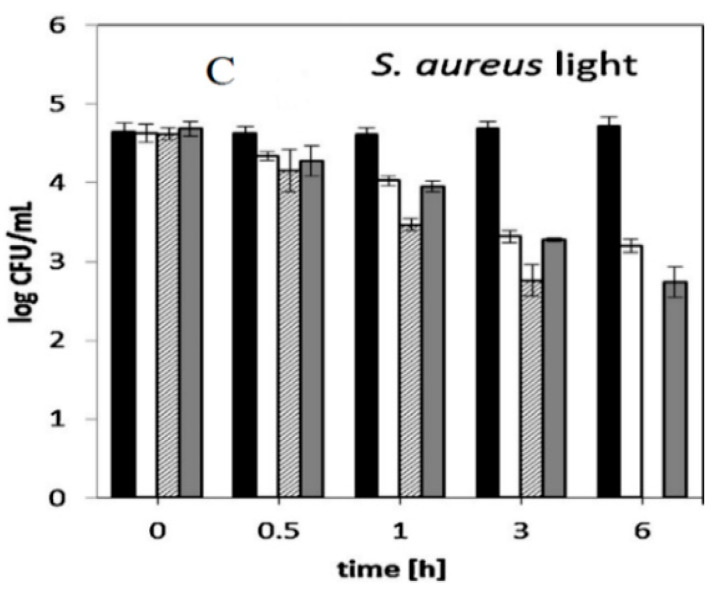
Log reduction of *E. coli* treated with undoped ZnO SG, ZnO:TFA 1:1 and ZnO:TFA 1:2 under (**A**) the dark and (**B**) visible light conditions. (**C**) Bactericidal activity of those samples against *S. aureus* under visible light. All tests were conducted in triplicates. Reproduced from [310] with permission of Elsevier.

Non-metal dopants can effectively extend the absorption band edge of ZnO to visible light region by introducing an intermediate energy level just above the VB of ZnO. The dopants narrow the bandgap and facilitate the separation of eletron-hole pairs [309]. Podporska-Carroll et al. studied antimicrobial behavior of F-doped ZnO nanopowders against *E. coli* and *S. aureus* under visible light illumination. F-doped ZnO NPs with two different molar ratios of ZnO: trifluoroacetic acid (TFA), i.e., ZnO:TFA 1:1 and ZnO:TFA 1:2, were prepared by the sol-gel process [310]. Figure 41A displays the bactericidal activity of undoped ZnO (ZnO SG), ZnO:TFA 1:1 and ZnO:TFA 1:2 nanopowders against *E. coli* in the dark. A few log reduction in bacterial density can be readily seen in these samples after testing for 3h and 6h. This is ascribed to the direct contact killing of undoped ZnO and F-doped ZnO through the release of Zn^2+^ ions and ROS generation. Under visible light irradiation for 6h, ZnO:TFA 1:1 produces a 2.88 log reduction (99.87%) in *E. coli* population, while it yields a 4.62 log reduction (over 99.99%) in *S. aureus* population (Figure 41B,C). The visible-light photocatalytic bactericidal activity derives from the generation of additional ROS as expected [310].

#### 4.2.3. Coupled Metal Oxide Semiconductors

**Figure 42:**
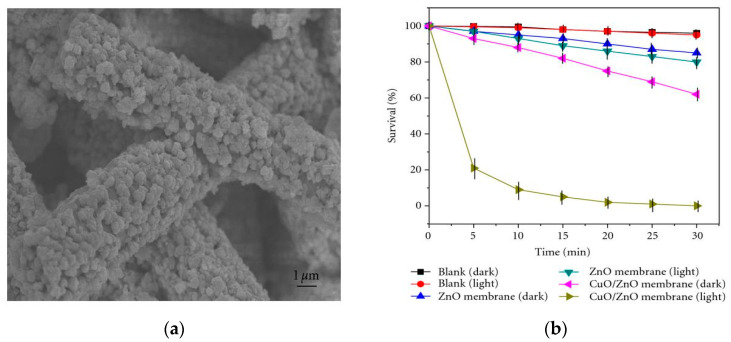
(**a**) SEM image showing ZnO nanorods decorated with CuO. (**b**) Survival rate vs visible light irradiation time curves of *E. coli* treated with CuO/ZnO membrane and ZnO membrane. Reproduced from [313] under the Creative Commons Attribution License.

Coupling nano-ZnO with CuO of a relatively narrow bandgap (~1.35–2.00 eV) can yield CuO/ZnO nanocomposite heterostructures with enhanced photocatalytic activity [311,312]. Liu et al. carried out antimicrobial study of pristine ZnO and CuO/ZnO nanocomposite membranes against *E. coli*. The composite membrane was prepared by mixing hydrothermally grown ZnO nanorods with an aqueous solution of copper sulfate and NaCl. The resulting ZnO nanorods were decorated with CuO NPs (~100 nm), showing a corn-like feature (Figure 42a) [313].Under dark condition, ZnO nanorod exhibited limited bactericidal activity associated with direct contact killing effect and the generation of a certain amount of ROS. The presence of intrinsic oxygen vacancies in ZnO nanorod favored the formation of superoxide anion, hydroxyl radicals, singlet oxygen and H_2_O_2_in the dark [20,21,302]. As such, ZnO nanorod inactivated *E. coli* around 15% in the dark after 30 min. In the case of CuO/ZnO under dark condition, the nanocomposite would kill around 40% *E. coli* after 30 min due to released Zn^2+^ ions, Cu^2+^ ions and the ROS generation. However, CuO/ZnO membrane inactivated *E. coli* completely after 25 min irradiation of visible light (Figure 42b). This was ascribed to the high generation of superoxide and hydroxyl radicals for killing *E. coli*. As mentioned previously, electrons were excited from the VB to CB of CuO under visible light illumination due to its small bandgap. Photoexcited electrons from the CB of CuO were then transferred to the CB of ZnO for producing superoxide anions, while the holes from the VB of ZnO were moved to the VB of CuO for creating hydroxyl radicals (Figure 10b).

#### 4.2.4. GO/ZnO Nanocomposites

As mentioned, photocorrosion of ZnO nanostructures can be prevented by modifying with carbonaceous nanomaterials such as graphene sheet and CNTs [89,160,161,162,314]. Furthermore, the optical absorption edge of nano-ZnO is red-shifted to the visible region by modifying with carbonaceous nanomaterials due to a significant decrease in their bandgap energy [172,314]. The main drawbacks of CNTs for fabricating CNTs/ZnO nanocomposites include the lack of solubility in aqueous media, and the absence of oxygenated functional groups for reacting with ZnO. By contrast, negatively charged oxygenated groups of GO react readily with released Zn^2+^ ions from the zinc salt precursor to produce GO/ZnO nanocomposites. This leads to the formation of finely dispersed ZnO NPs on graphene [242].

**Figure 43:**
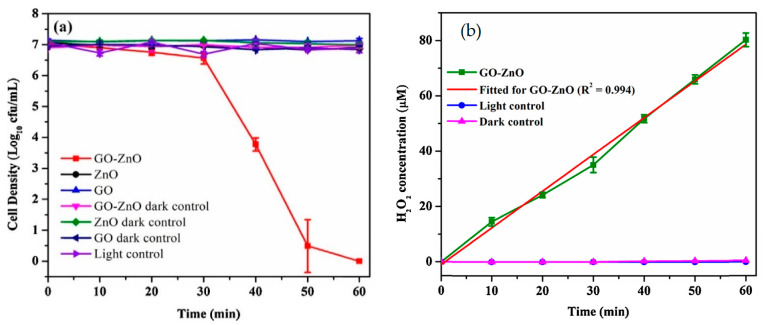
(**a**) Visible-light photocatalytic inactivation efficiency of *E. coli* (1 × 10^7^ CFU/ mL) treated with GO/ZnO composite, GO and ZnO; (**b**) H_2_O_2_ generation from GO/ZnO during the photocatalytic inactivation process under visible light. All the experiments and controls were conducted in triplicates. Reproduced from [173] with permisiion of Elsevier.

Wu et al. hydrothermally synthesized GO/ZnO hybrid using an aqueous solution of GO and ZnO, and reported its visible-light photocatalytic bactericidal activity [173]. Under visible light illumination, total inactivation of *E. coli* requires 60 min, with a 7-log reduction in bacterial cell density (Figure 43a). As such, electrons are injected from the graphene sheet into the CB of ZnO, generating superoxide anion on the GO/ZnO nanocomposite (Figure 8). Meanwhile, H_2_O_2_ is formed by the reduction of superoxide anion. Figure 43b shows the creation of H_2_O_2_ from GO/ZnO during the photocatalytic inactivation process under visible light illumination. The hydrogen peroxide content can reach as high as 80 µM within 60 min, leading to remarkable photocatalytic bactericidal activity accordingly. In general, negatively charged superoxides and hydroxyl radicals cannot pass through bacterial cell membrane with a negative surface charge. Therefore, these radicals reside mainly on the surface of bacterial cell wall for bacterial killing. However, neutral H_2_O_2_ molecule permeates the cell wall readily, which is followed by the subsequent oxidation of intracellular components.

## 5. Hemolysis

Malaria is caused by protozoa of the genus *Plasmodium* through the bites of female *Anopheles* mosquitoes. Protozoa are single celled organisms having membrane-bound nuclei. In particular, *Plasmodium falciparum* is the deadliest parasite causing human fatality globally. Those parasites invade mammalian erythrocytes (red blood cells), causing rupture of infected cells. So much efforts have been spent by the researchers to tackle this issue. Graphene and its derivatives GO/rGO have been reported to inhibit malaria invasion through physical barrier obstruction of *P. falciparum* parasites and nutrition depletion effects [315,316]. Recently, Paul et al. have employed CNT/ZnO hybrid for the detection of malaria biomarker, histidine rich protein II (HRP2) [317]. HRP2 is a 30 kDa water-soluble protein found on the surface of infected red blood cells (RBCs) [318]. So CNT/ZnO hybrid facilitates not only the formation of ROS for killing bacteria, but also acts as an effective biosensor for detecting malaria.

### 5.1. Red Blood Cells

**Figure 44:**
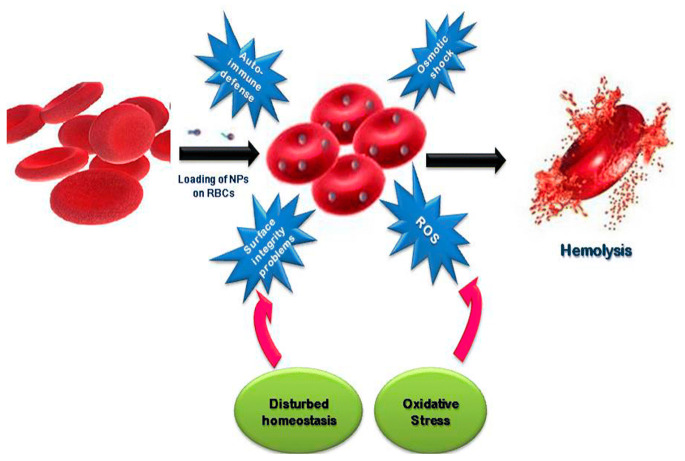
Hemolysis is caused by the binding or encapsulation of nanoparticles (NPs) in red blood cells (RBCs) leading to the generation of ROS, oxidative stress, and disturbed homeostasis as a result of osmotic shock and surface integrity problems. Reproduced from [320] with permission of Springer Nature.

In a previous article, we have reviewed the cytotoxic effects of ZnO nanostructures on mammalian cells [67]. As mentioned, ZnO NPs are widely used in cosmetics, suncreams, UV-absorbing packaging films and fabrics for food and medical textile applications, as well as therapeutic agents for cancer treatment [42,43,44,45,46,67]. In addition, ZnO NPs have also been added to animal (e.g., chicken) feed to promote the growth performance of the animals [319]. So ZnO NPs could enter the human body by different routes including the circulatory system. Once ZnO NPs enter the circulatory system, they come in direct contact with RBCs, neutrophils and immune cells. As it is known, mature erythrocytes do not have nucleus and rough endoplasmic reticulum in order to accommodate a large amount of hemoglobin in the cells. In this respect, erythrocytes are incapable of replacing damaged proteins and undergoing mitosis. However, camels, birds, and fish have nucleated red blood cells. By contacting RBCs, ZnO nanoparticles would anchor on their surfaces and penetrate through the cell membranes, resulting in the generation of ROS. In this respect, RBCs are vulnerable to oxidative stress damage. This leads to the disruption of erythrocyte membranes, which causes the leak of hemoglobin into surrounding medium. This is generally referred to as hemolysis (Figure 44) [320].

The biocompatibility of nanomaterials contacting blood in medical applications can be assessed using the ASTM standards. The hemoglobin released from damaged red blood cells due to nanomaterials is determined accordingly. From the ASTM E2524-08 standard, nanoparticulate materials cause damage to RBCs when hemolysis exceeds 5% [321]. ASTM F756 standard further describes hemolytic criteria of blood-contacting nanoparticulate materials, i.e., <2% as non-hemolytic, 2–5% as slightly hemolytic, and >5% as hemolytic [322].

**Figure 45:**
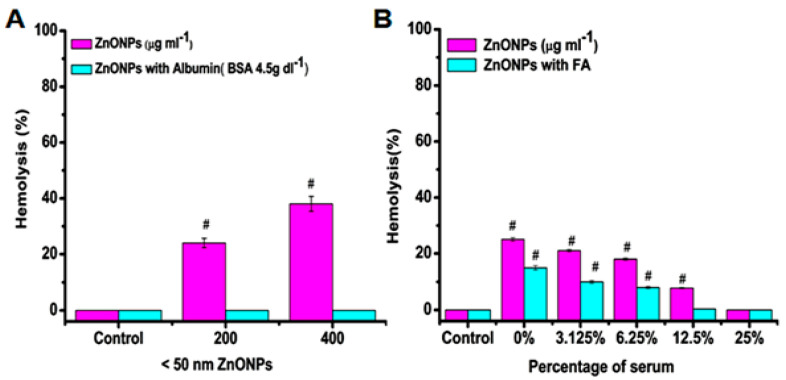
(**A**) Hemolytic effect of ZnO NPs (<50 nm) of two different doses with and without albumin (4.5 μg dl^−1^) on erythrocytes for 24 h. # denotes significant difference at *p* ≤ 0.05 of the samples compared to control. (**B**) The effect of ZnO NPs (<50 nm; 200 μg/mL) and ZnO NPs with ferulic acid (FA) exposure on hemolysis in the presence of fetal bovine serum of different concentrations (3.125%, 6.25%, 12.5% and 25%) for 24 h. # denotes significant difference at *p* ≤ 0.05 of the samples compared to control. Reproduced from [323] under Creative Commons Attribution License.

Preedia Babu et al. studied the cytotoxic effect of commercial ZnO NPs on erythrocytes. ZnO NPs of different sizes, i.e., <50 nm, 50–100 nm and >100 nm of were separated by means of density gradient centrifugation. The hemolytic effect of ZnO NPs (<50 nm) in the presence of albumin and ferulic acid (FA) was examined [323]. FA is a natural phytochemical from the plants, showing good anti-inflammatory response and antioxidant activity [324]. Figure 45A shows the hymolysis rate of chicken RBCs treated with ZnO NPs (<50 nm) of 200 and 400 µg/mL concentrations. A significant hemolytic activity of around 24% and 38% can be readily observed. This is due to the internalization of small ZnO NPs by RBCs as observed by TEM, leading to the ROS generation. However, no hemolysis is observed in the presence of albumin. Furthermore, hemolytic activity is also assessed at different fetal bovine serum (FBS) concentrations ranging from 3.125% to 25% (Figure 45B). Apparently, hemolysis decreases with increasing percentage of serum. Thus antioxidant FA also reduces ZnO NPs induced hemolysis.

**Figure 46:**
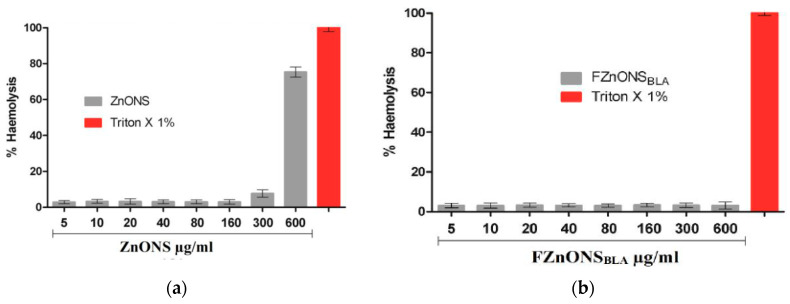
Hemocompatibility assay results of human erythrocytes treated with (**a**) ZnO nanostrucuture (ZnONS) and (**b**) bovine α-lactalbumin (BLA) functionalized ZnONS of different concentrations. Trixton X 1% was employed as a control. All the data were expressed as mean ± standard deviation, *n* = 3. Reproduced from [326] with permission of Elsevier.

Khan et al. synthesized ZnO nanorods by means of combustion of zinc acetate and citric acid gel. The percentage hemolysis due to the nanorods at doses of 50, 100 and 250 µg/mL was determined to be 20, 39.5 and 62.5%, respectively [325]. Mahanta et al. prepared ZnO NPs (100–250 nm; average size of 200 nm) using co-precipitation process, and then functionalized nanoparticles with a small protein bovine α-lactalbumin (BLA) to yield FZnONS_BLA_. BLA was used to enhance cytocompatibility of ZnO NPs (ZnONS) [326]. Hemocompatibility assay was employed to assess hemolytic behavior of human RBCs treated with ZnONS and FZnONS_BLA_ of different concentrations. Minimal hemolysis (<5%) was found at ZnONS doses below 300 μg/mL. Hemolysis rate rose to 7.7% and 75.3% by increasing ZnONS doses to 300 μg/mL and 600 μg/mL, respectively (Figure 46a). FZnONS_BLA_ showed negligible hemolysis (<3%) up to a concentration of 600 μg/mL), implying hemocompatible of FZnONS_BLA_ with doses from 5 to 600 μg/mL (Figure 46b).

#### Inhibition of Hemolysis

**Figure 47:**
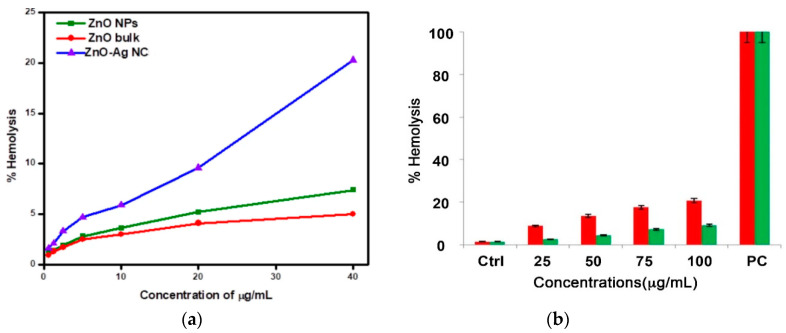
(**a**) The hemolytic activity of bulk ZnO, biosynthesized ZnO NPs and AgNPs/ZnO nanocomposite. Reproduced from [282] under the terms of Creative Commons License. (**b**) Percentage hemolysis of ZnO nanoparticles prepared by biosynthesis (green column) and co-precipitation (orange column). 0.1% Triton X-100 was employed as a positive control (PC). Reproduced from [328] with permission of Springer.

As discussed in Figure 26, biosynthesized AgNPs/ZnO hybrid exhibits excellent antibacterial activity than pure ZnO NPs against *S. aureus* and *E. coli*, especially for former bacterial strain. This is evidenced by bacterial viability and the zone of inhibition results [282]. The excellent bactericidal activity of green AgNPs/ZnO NPs hybrid derives from the release of silver ions from AgNPs and Zn^2+^ ions from ZnO to the culture medium. However, released Ag^+^ ions from AgNPs of the hybrid are also toxic to various mammalian cell lines [14]. Figure 47a shows the percentage hemolysis of RBCs upon exposure to bulk ZnO, green ZnO NPs and AgNPs/ZnO nanocomposite for 2h. It is apparent that AgNPs/ZnO hybrid possesses higher hemolytic activity than ZnO NPs and bulk ZnO. The hemolytic activity increases markedly with increasing AgNPs/ZnO doses, demonstrating dose-dependent hemolysis. Thus, AgNPs cause the lysis of RBCs rather than green ZnO from the hybrid. From the literature, AgNPs cause a dose-dependent hemolysis in RBCs due to the generation of ROS and oxidative stress [327]. In general, standalone ZnO NPs biosynthesized from the plant extracts are more effective to prevent hemolysis of RBCs than chemically prepared ZnO NPs. Very recently, Mahalakshmi et al. biosynthesized ZnO NPs using zinc acetate and *Sesbania grandiflora* leaf extract. In addition, ZnO NPs were also synthesized via co-precipitation method. They then treated human RBCs with green ZnO NPs and chemically prepared ZnO NPs [328]. The hemolytic activity of human RBCs exposed to both types of ZnO NPs was depicted in Figure 47b.

Jan et al. treated human erythrocytes with green ZnO NPs (19.58 nm); ZnO NPs were biosynthesized from zinc acetate dihydrate and leaf extract of *Aquilegia pubiflora* acting as an effective reducing and capping agent. The leaf extract contained several medicinal phytochemicals including flavonoids (orientin, isoorientin, isovitexin and vitexin), and hydroxycinnamic acid derivatives (ferulic acid, coumaric acid, and sinapic acid) [329]. The hemolytic activity of green ZnO NPs at doses of 50, 100, 200 and 400 μg/mL was reported to be 0.48 ± 0.23%, 0.73 ± 0.1%, 1.05 ± 0.12% and 1.24 ± 0.14%, respectively. Those phytochemicals rendered green ZnO NPs with insignificant hemolytic activity, i.e., biocompatible with erythrocytes. From a recent study of Rajapriya et al., spherical ZnO NPs (65.9 nm) synthesized from *Cynara scolymus* leaf extract at a dose of 100 μg/mL also had a low hemolytic activity of 0.5% for human RBCs [330]. Similarly, green ZnO NPs (26.55 nm) biosynthesized from aqueous zin acetate and *Costus igneus* leaf extract were biocompatible with goat RBCs as evidenced by low hemolytic activity. From hemolytic assay, the percentage hemolysis due to the nanoparticles at doses of 50, 100, 150 and 200 μg/mL was reported to be 0.536 ± 0.005, 0.583 ± 0.005, 0.595 ± 0.003, and 0.633 ± 0.005, respectively [331]. Overall, these results clearly demonstrate that ZnO NPs biosynthesized from the leaf extracts have no adverse effect towards RBCs. They can be classified as non-hemolytic on the basis of ASTM F756 standard, i.e., the percentage hemolysis is <2% [322]. Table 2 summarizes the percentage hemolysis of RBCs upon exposure to nano-ZnO prepared from different processes.

**Figure 48:**
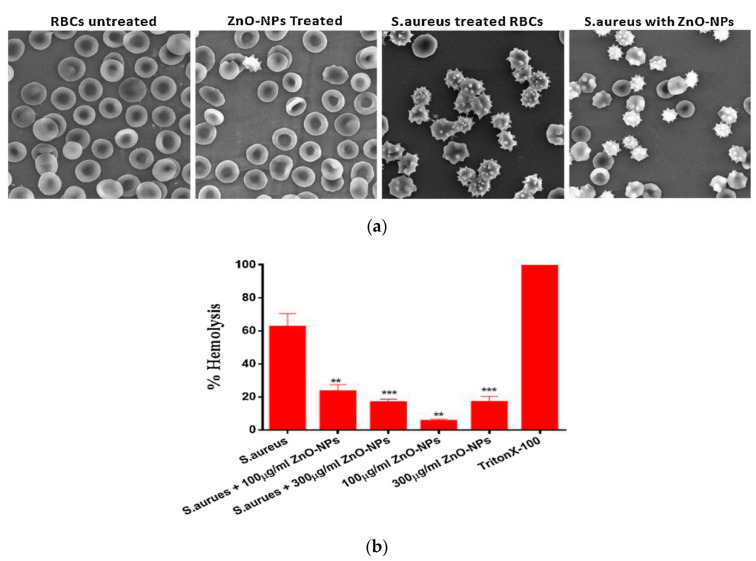
(**a**) SEM images showing lysis of RBCs by *S. aureus* and their inhibition by green ZnO NPs. Images from left to right: untreated RBCs, ZnO NPs treated RBCs, *S. aureus* infected RBCs, and RBCs co-cultured with *S. aureus* and ZnO NPs. (**b**) Inhibition of hemolysis in *S. aureus* infected RBCs by ZnO NPs. All data are expressed as the mean ± standard deviation. *** for *p* ≤ 0.001; ** for *p* ≤ 0.01. Reproduced from [332] under a Creative Commons License.

Finally, phytochemicals capped on ZnO NPs are also beneficial for reducing hemolysis of *S. aureus* infected RBCs. Very recently, Ahmar Rauf et al. biosynthesized ZnO NPs (10–50 nm) using zinc acetate and *Bougainvillea* flower extract. They then exposed RBCs to either green ZnO NPs, *S. aureus*, or combined *S. aureus* with ZnO NPs [332]. The virulence factor of *S. aureus* produces α-hemolysin, leading to the pore formation in the plasma membranes of RBCs (Figure 48a). As such, *S. aureus* infected RBCs undergo a high hemolysis rate of 61%. The hemolysis of RBCs reduces markedly to about 21% by co-culturing green ZnO NPs at a dose of 100 µg/mL with *S. aureus*. A further reduction in hemolysis of *S. aureus* infected RBCs can be achieved by treating with ZnO NPs at a dose of 300 µg/mL (Figure 48b).

## 6. Immune Cells

When ZnO NPs enter human body, they also come in direct contact with immune cells like macrophages, neutrophils, B-cells, T-cells, etc. The macrophages play an important role in innate and adaptive immunity for defending the host immune against foreign antigens through the recognition, processing and elimination. Macrophages remove foreign antigens from the tissues by means of phagocytosis [333]. In this respect, ZnO NPs with nanoscale dimension are phagocyted readily by macrophages and dissolved in lysosomes, releasing zinc ions accordingly. ZnO NPs can interact with white blood cells to activate innate immune system by triggering inflammatory responses and releasing pro-inflammatory cytokines such as tumor necrosis factor-alpha (TNF-α), interleukin-6 (IL-6), and interleukin-1β (IL-1β). The IL-1β activates macrophages at the inflammation site. This is followed by the subsequent secretion of other cytokines including TNF-α and IL-6. For instance, Chang et al. reported that ZnO NPs can induce lung inflammation by releasingTNF-α and IL-6 via toll-like receptor (TLR) signaling pathways. Instillation of ZnO NPs into the tracheas of mice causes increased neutrophils and macrophages in bronchoalveolar lavage fluid (BALF), bronchioles and peribronchiolar areas [334]. In a recent study, Alghsham et al. also reported that ZnO NWs induce the production of TNF-α and IL-6 in cultured mouse macrophage cell line (RAW 264.7) and bone marrow macrophages obtained from the mice hind legs. From in vivo mouse model results, ZnO NWs facilitated the recruitment of macrophages and eosinophils into the lung and air-pouch [335].

The mechanisms responsible for cytotoxicity of ZnO nanomaterials on immune cells include the generation of ROS, the release of zinc ions, and the membrane lipid peroxidation [336,337,338,339]. The extent of nanotoxicity depends on the size, shape and concentration of ZnO nanomaterials. In particular, spherical ZnO NPs (10–30 nm) are more toxic than ZnO nanorods as expected [337]. Johnson et al. indicated that release of Zn^2+^ ions from ZnO NPs triggers the generation of excessive intracellular ROS, resulting in autophagic death of immune cells [338]. Roy et al. treated mouse primary peritoneal macrophages with ZnO NPs (∼50 nm), resulting in the induction of ROS and membrane lipid peroxidation. These activated autophagy and apoptosis as evidenced by the cleavage of apoptotic caspases-3, -8, and -9. Furthermore, the apoptotic and autophagic cell death were inhibited considerably by treating with N-acetylcysteine (NAC) for blocking the generation of ROS [339]. NAC was reported to be an effective antioxidant for inhibiting ROS generation, thus acting as ROS scavenger for macrophages [340]. The cytotoxicity of ZnO NPs in human acute monocytic leukemia cell line (THP-1) was also reduced through surface modification with polyethylene glycols (PEGs). PEGylation of ZnO NPs led to a decreased uptake of modified nanoparticles in THP-1 monocytes and macrophages by inhibiting the binding of NPs to blood proteins and macrophages [341].

Apart from NAC and PEGylation, green ZnO NPs synthesized from the plant extracts exhibit excellent anti-inflammatory properties by reducing the secretion of proinflammatory cytokines dramatically [342,343,344]. The anti-inflammatory activity of ZnO NPs is comparatively higher than that of AgNPs with 79% and 69.1% respectively [343]. Meanwhile, green ZnO NPs synthesized from *Vernonia amygdalina* leaves show good anti-inflammatory properties in the mice model due to the presence of flavonoids and tannins in the plant extracts. As a result, the inflammatory activity and pro-inflammatory cytokine level in the mice are reduced substantially [344]. In this respect, green ZnO NPs with good antimicrobial and anti-inflammatory properties are promising nanomaterials for the treatment of bacterial infections. Considering ZnO as an additive to improve the flavor, color, nutritional value and shelf-life of food, and a source of zinc in supplements, the risks of using ZnO NPs can be minimized by using nanoparticles biosynthesized from natural plant derivatives.

## 7. Conclusions

This review presents a comprehensive summary of literature studies on the fabrication, antibacterial activity, and photocatalytic bacterial inactivation of ZnO heterostructures under visible light. ZnO nanostructures of different dimensions and shapes with hexagonal wurtzite lattice can be prepared by means of vapor-, liquid-, and solid-phase processing techniques. Among these, wet chemical processing techniques including co-precipitation, hydrothermal/solvothermal treatment, hydrolysis-condensation, and microemulsion are a facile route to synthesize ZnO nanomaterials on a large scale at lower temperatures and costs compared to vapor-phase method.

ZnO nanostructures with positive surface charge are able to adhere and attach on negatively charged membrane via electrostatic interaction when they come in contact with bacteria. This effect disrupts bacterial cell membrane function, interferes electron transport chain, and deactivates bacterial enzyme, leading to final cell death. Apart from contact killing effect, other mechanisms such as the ROS production and released zinc ions have also been reported to be responsible for bactericidal activity of ZnO nanomaterials. The antibacterial activity of ZnO nanorod arrays against *E. coli* derives from the combined effect of these three mechanisms in which direct contact killing effect predominates [288]. The antimicrobial activity of ZnO nanostructures is size-, shape-, and concentration- dependent. In particular, green ZnO nanoparticles have the lowest MIC values against Gram-positive and Gram-negative bacterial strains compared with ZnO NPs prepared by hydrothermal/solvothermal, sol-gel and polyol techniques (Table 1). This can be attributed to green ZnO NPs exhibiting high purity and possessing bioactive phytocompounds, such as flavonoids and polyphenols for bactericidal activity [216,217,229]. Furthermore, phytochemicals of the plant extracts are very effective to reduce hemolysis of *S. aureus* infected RBCs and inflammatory response of white blood cells.

The modifications in nano-ZnO can be employed to enhance photocatalytic activity for the ROS generation. ZnO heterostructures modified with metal/non-metal dopants, carbon nanomaterials and other semiconductors show excellent photocatalytic bacterial inactivation under visible light. In particular, coupling nano-ZnO with CuO can yield CuO/ZnO nanocomposite with enhanced photocatalytic activity under visible light. As such, photoexcited electrons are injected from CuO to the conduction band of ZnO NPs for generating reactive radical species. Consequently, 100% *E. coli* inactivation under visible light takes only 25 min [313]. Moreover, GO/ZnO nanocomposite also exhibits excellent photocatalytic bactericidal activity, showing a complete inactivation of *E. coli* in 60 min, with a 7-log reduction in bacterial cell density under visible light illumination [173].

## Figures and Tables

**Table 1:** Minimal inhibitory concentration (MIC) of ZnO nanostructures prepared by different processes.

Material	Size (nm)	Shape	Synthesis Process	Bacterial Strains	MIC, µg/mL	Reference
ZnO	~16.66	Spherical	Green	*Escherichia coli*	25	[30]
				*Pseudomonas aeruginosa*	25	
				*Staphylococcus aureus*	6.25	
				*Bacillus subtilis*	6.25	
ZnO	~18	Spherical	Green	*Escherichia coli*	15.625	[31]
				*Pseudomonas aeruginosa*	31.25	
				*Klebsiella pneumonia*	125	
				*Staphylococcus aureus*	15.625	
				*Bacillus subtilis*	7.8	
ZnO	44	Nanoflower	Green	*Pseudomonas aeruginosa*	≥100	[281]
				*Staphylococcus aureus*	33.33	
ZnO	14.63	Spherical	Polyol	*Escherichia coli*	625	[27]
ZnO	~10^3^ (length of nanorods)	Nanoflower formed by self-assembly of rods	Microwave-assited hydrothermal	*Escherichia coli*	1250	[27]
ZnO	170	Spherical	Co-precipitation	*Escherichia coli*	50	[240]
				*Salmonella typhimurium*	50	
				*Bacillus subtilis*	100	
				*Enterococcus faecalis*	200	
ZnO	19	Spherical	Sol-gel	*Escherichia coli*	500	[19]
				*Klebsiella pneumoniae*	500	
ZnO	ZnO: 5.3 Heat-treated ZnO: 33.9	Spherical Spherical	Sol-gel	*Escherichia coli*	312.5	[26]
				*Staphylococcus aureus*	78.2	
				*Escherichia coli*	1250	
				*Staphylococcus aureus*	208.2	
ZnO	100–250 (diameter); 50–80 (thickness)	Prismatic nanoplates	Hydrothermal	*Escherichia coli*	82	[28]
				*Staphylococcus aureus*	78	
ZnO	10^3^ (length)	Tapered rod (Spindle)	Microwave-assited solvothermal	*Escherichia coli*	180	[115]
				*Salmonella typhimurium*	160	
				*Pseudomonas aeruginosa*	>1000	
				*Proteus vulgaris*	>1000	
				*Staphylococcus aureus*	64	
				*Bacillus subtilis*	60	
7.7%AgNPs/ZnO	10^3^ (length) of ZnO nanorods	Dispersion of AgNPs on ZnO nanorods	Microwave-assited solvothermal & deposition	*Escherichia coli*	40	[115]
				*Salmonella typhimurium*	60	
				*Pseudomonas aeruginosa*	200	
				*Proteus vulgaris*	60	
				*Staphylococcus aureus*	16	
				*Bacillus subtilis*	20	
5%AgNPs/ZnO	180–220 (diameter); 70–80 (thickness)	Prismatic nanoplates	Hydrothermal	*Escherichia coli*	38	[28]
				*Staphylococcus aureus*	40	
5%Cu/ZnO	250–350 (diameter); 60–80 (thickness)	Prismatic nanoplates	Hydrothermal	*Escherichia coli*	72	[28]
				*Staphylococcus aureus*	62	
GO/ZnO	ZnO: 170	Dispersion of ZnO NPs on GO sheet	Solution mixing	*Escherichia coli*	6.25	[240]
				*Salmonella typhimurium*	6.25	
				*Bacillus subtilis*	12.5	
				*Enterococcus faecalis*	25	

**Table 2:** The percentage hemolysis of erythrocytes treated with ZnO nanostructures.

Material	Size, nm	Shape	Synthetic Process	Dose, µg/mL	Percentage Hemolysis	Ref.
ZnO	<50	Particles	Commercial	200	24	[323]
	<50	Particles	Commercial	400	38	[323]
ZnO + albumin	<50	Particles	Commercial	200	<2	[323]
ZnO + albumin	<50	Particles	Commercial	400	<2	[323]
ZnO	47.8–52.5 (width)	Rod	Gel combustion	50	20	[325]
	47.8–52.5 (width)	Rod	Gel combustion	100	39.5	[325]
	47.8–52.5 (width)	Rod	Gel combustion	250	65.2	[325]
ZnO	200	Particles	Co-precipitation	5 to 160	<5	[326]
	200	Particles	Co-precipitation	300	7.7	[326]
	200	Particles	Co-precipitation	600	75.3	[326]
FZnONS_BLA_	450	Particles	Co-precipitation	5 to 600	<3	[326]
ZnO	19.58	Particles	Green	50	0.48 ± 0.23	[329]
	19.58	Particles	Green	100	0.73 ± 0.1	[329]
	19.58	Particles	Green	200	1.05 ± 0.12	[329]
	19.58	Particles	Green	400	1.24 ± 0.14	[329]
ZnO	65.9	Particles	Green	100	0.5	[330]
ZnO	26.55	Particles	Green	50	0.536 ± 0.005	[331]
	26.55	Particles	Green	100	0.583 ± 0.005	[331]
	26.55	Particles	Green	150	0.595 ± 0.003	[331]
	26.55	Particles	Green	200	0.633 ± 0.005	[331]
AgNPs/ZnO hybrid	5 (AgNPs)	Particles	Bio-hydrothermal	10	~6.0	[282]
	5 (AgNPs)	Particles	Bio-hydrothermal	20	~9.1	[282]
	5 (AgNPs)	Particles	Bio-hydrothermal	40	~20.5	[282]

## References

[1] Fletcher S. (2015). Understanding the contribution of environmental factors in the spread of antimicrobial resistance. Environ. Health Prev. Med..

[2] Gill A.S., Morrissey H., Rahman A. (2018). A systematic review and meta-analysis evaluating antibiotic prophylaxis in dental implants and extraction procedures. Medicina.

[3] Pang Z., Raudonis R., Glick B.R., Lin T.J., Cheng Z. (2019). Antibiotic resistance in Pseudomonas aeruginosa: Mechanisms and alternative therapeutic strategies. Biotechnol. Adv..

[4] Levin-Reisman I., Brauner A., Ronin I., Balaban N.Q. (2019). Epistasis between antibiotic tolerance, persistence, and resistance mutations. Proc. Nat. Acad. Sci. USA.

[5] O’Gara J.P. (2017). Into the storm: Chasing the opportunistic pathogen Staphylococcus aureus from skin colonisation to life-threatening infections. Environ. Microbiol..

[6] Li B., Webster T.J. (2018). Bacteria antibiotic resistance: New challenges and opportunities for implant-associated orthopaedic infections. J. Orthop. Res..

[7] Craft K.M., Nguyen J.M., Berg L.J., Townsend S.D. (2019). Methicillin-resistant Staphylococcus aureus (MRSA): Antibiotic-resistance and the biofilm phenotype. Med. Chem. Commun..

[8] Beyth N., Houri-Haddad Y., Domb A., Khan W., Hazan R. (2015). Alternative antimicrobial approach: Nano-antimicrobial materials. Evid. Based Complement. Altern. Med..

[9] Hemeg H.A. (2017). Nanomaterials for alternative antibacterial therapy. Int. J. Nanomed..

[10] Choudhari P., Das S.K. (2019). Bio-reduced graphene oxide as a nanoscale antimicrobial coating for medical devices. ACS Omega.

[11] Tjong S.C., Chen H. (2004). Nanocrystalline materials and coatings. Mater. Sci. Eng. R Rep..

[12] Tjong S.C. (2013). Nanocrystalline Materials: Their Synthesis-Structure-Property Relationships and Applications.

[13] Liao C., Li Y., Tjong S.C. (2018). Graphene nanomaterials: Synthesis, biocompatibility, and cytotoxicity. Int. J. Mol. Sci..

[14] Liao C., Li Y., Tjong S.C. (2019). Bactericidal and cytotoxic properties of silver nanoparticles. Int. J. Mol. Sci..

[15] Limo M.J., Sola-Rabada A., Boix E., Thota V., Westcott J.C., Puddu V., Perry C.C. (2018). Interactions between metal oxides and biomolecules: From fundamental understanding to applications. Chem. Rev..

[16] Baptista P.V., McCusker M.P., Carvalho A.G., Ferreira D.A., Mohan N.M., Martins M., Fernandes A.R. (2018). Nano-strategies to fight multidrug resistant bacteria—“A battle of the titans”. Front. Microbiol..

[17] Yan X., He B., Liu L., Qu G., Shi J., Hu L., Jiang G. (2018). Antibacterial mechanism of silver nanoparticles in Pseudomonas aeruginosa: Proteomics approach. Metallomics.

[18] Chatterjee A.K., Chakraborty R., Basu T. (2014). Mechanism of antibacterial activity of copper nanoparticles. Nanotechnology.

[19] Ansari M.A., Khan H.M., Khan A.A., Sultan A., Azam A. (2012). Synthesis and characterization of the antibacterial potential of ZnO nanoparticles against extended-spectrum β-lactamases-producing Escherichia coli and Klebsiella pneumoniae isolated from a tertiary care hospital of North India. Appl. Microbiol. Biotechnol..

[20] Lipovsky A., Tzitrinovich Z., Friedmann H., Applerot G., Gedanken A., Lubart R. (2009). EPR study of visible light-induced ROS generation by nanoparticles of ZnO. J. Phys. Chem. C.

[21] Prasanna V.L., Vijayaraghavan R. (2015). Insight into the mechanism of antibacterial activity of ZnO: Surface defects mediated reactive oxygen species even in the dark. Langmuir.

[22] Joe A., Park S.H., Shim K.D., Kim D.J., Jhee K.H., Lee H.W., Heo C.H., Kim H.M., Jang E.S. (2017). Antibacterial mechanism of ZnO nanoparticles under dark conditions. J. Ind. Eng. Chem..

[23] Chauhan A., Verma R., Kumari S., Sharma A., Sandilya P., Li X., Batoo K.M., Imran A., Kulshrestha S., Kumar R. (2020). Photocatalytic dye degradation and antimicrobial activities of pure and Ag-doped ZnO using Cannabis sativa leaf extract. Sci. Rep..

[24] Liao C., Li Y., Tjong S.C. (2020). Visible-light active titanium dioxide nanomaterials with bactericidal properties. Nanomaterials.

[25] Wang L., Hu C., Shao L. (2017). The antimicrobial activity of nanoparticles: Present situation and prospects for the future. Int. J. Nanomed..

[26] Da Silva B.L., Caetano B.L., Chiari-Andréo B.G., Pietro R.C., Chiavacci L.A. (2019). Increased antibacterial activity of ZnO nanoparticles: Influence of size and surface modification. Colloids Surf. B Biointerfaces.

[27] Paduraru A., Ghitulica C., Trusca R., Surdu V.A., Neacsu I.A., Holban A.M., Birca A.C., Iordache F., Vasile B.S. (2019). Antimicrobial wound dressings as potential materials for skin tissue regeneration. Materials.

[28] Abinaya C., Marikkannan M., Manikandan M., Mayandi J., Suresh P., Shanmugaiah V., Ekstrum C., Pearce J.M. (2016). Structural and optical characterization and efficacy of hydrothermal synthesized Cu and Ag doped zinc oxide nanoplate bactericides. Mater. Chem. Phys..

[29] Dwivedi S., Wahab R., Khan F., Mishra Y.K., Musarrat J., Al-Khedhairy A.A. (2014). Reactive oxygen species mediated bacterial biofilm inhibition via zinc oxide nanoparticles and their statistical determination. PLoS ONE.

[30] Elumalai K., Velmurugan S. (2015). Green synthesis, characterization and antimicrobial activities of zinc oxide nanoparticles from the leaf extract of Azadirachta indica (L.). App. Surf. Sci..

[31] Abbasi B.A., Iqbal J., Ahmad R., Zia L., Kanwal S., Mahmood T., Wang C., Chen J.-T. (2020). Bioactivities of Geranium wallichianum leaf extracts conjugated with zinc oxide nanoparticles. Biomolecules.

[32] Paladini F., Pollini M. (2019). Antimicrobial silver nanoparticles for wound healing application: Progress and future trends. Materials.

[33] Tarannum N., Gautam Y.K. (2019). Facile green synthesis and applications of silver nanoparticles: A state-of-the-art review. RSC Adv..

[34] Gliga A.R., Skoglund S., Odnevall Wallinder I., Fadeel B., Karlsson H.L. (2014). Size-dependent cytotoxicity of silver nanoparticles in human lung cells: The role of cellular uptake, agglomeration and Ag release. Part. Fibre Toxicol..

[35] Kandpal K., Gupta N. (2016). Investigations on high-κ dielectrics for low threshold voltage and low leakage zinc oxide thin-film transistor, using material selection methodologies. J. Mater. Sci. Mater. Electron..

[36] Janotti A., Van de Walle C.G. (2009). Fundamentals of zinc oxide as a semiconductor. Rep. Prog. Phys..

[37] Wang B., Huang W., Chi L., Al-Hashimi M., Marks T.J., Facchetti A. (2018). High-k gate dielectrics for emerging flexible and stretchable electronics. Chem. Rev..

[38] Rauwel E., Galeckas A., Rauwel P. (2014). Photoluminescent cubic and monoclinic HfO_2_ nanoparticles: Effects of temperature and ambient. Mater. Res. Express.

[39] Zou Y., Zhang Y., Hu Y., Gu H. (2018). Ultraviolet detectors based on wide bandgap semiconductor nanowire: A review. Sensors.

[40] Chen C., Zhou P., Wang N., Ma Y., San H. (2018). UV-assisted photochemical synthesis of reduced graphene oxide/ZnO nanowires composite for photoresponse enhancement in UV photodetectors. Nanomaterials.

[41] Wu D., Wang X., Cao K., An Y., Song X., Liu N., Xu F., Gao Z., Jiang K. (2017). ZnO nanorods with tunable aspect ratios deriving from oriented-attachment for enhanced performance in quantum-dot sensitized solar cells. Electrochim Acta.

[42] Ghamsari M.S., Alamdari S., Han W., Park H.H. (2016). Impact of nanostructured thin ZnO film in ultraviolet protection. Int. J. Nanomed..

[43] Zhang G., Xiao Y., Yan J., Xie N., Liu R., Zhang Y. (2019). Ultraviolet light-degradation behavior and antibacterial activity of polypropylene/ZnO nanoparticles fibers. Polymers.

[44] Fouda A., Hassan S.E.D., Salem S.S., Shaheen T.I. (2018). In-vitro cytotoxicity, antibacterial, and UV protection properties of the biosynthesized Zinc oxide nanoparticles for medical textile applications. Microb. Pathog..

[45] Kim I., Viswanathan K., Kasi G., Sadeghi K., Thanakkasaranee S., Seo J. (2019). Poly(lactic Acid)/ZnO bionanocomposite films with positively charged ZnO as potential antimicrobial food packaging materials. Polymers.

[46] Abbas M., Buntinx M., Deferme W., Peeters R. (2019). (Bio)polymer/ZnO nanocomposites for packaging applications: A review of gas barrier and mechanical properties. Nanomaterials.

[47] Meng Y.Z., Tjong S.C. (1998). Rheology and morphology of compatibilized polyamide 6 blends containing liquid crystalline copolyesters. Polymer.

[48] Meng Y.Z., Tjong S.C., Hay A.S., Wang S.J. (2001). Synthesis and proton conductivities of phosphonic acid containing poly-(arylene ether)s. J. Polym. Sci. A Polym. Chem..

[49] Tjong S.C., Meng Y.Z. (1997). Morphology and mechanical characteristics of compatibilized polyamide 6-liquid crystalline polymer composites. Polymer.

[50] Liu C., Chan K.W., Shen J., Liao C., Yeung K.W.K., Tjong S.C. (2016). Polyetheretherketone hybrid composites with bioactive nanohydroxyapatite and multiwalled carbon nanotube fillers. Polymers.

[51] Chan K.W., Liao C., Wong H.M., Yeung K.W.K., Tjong S.C. (2016). Preparation of polyetheretherketone composites with nanohydroxyapatite rods and carbon nanofibers having high strength, good biocompatibility and excellent thermal stability. RSC Adv..

[52] Liao C., Li K., Wong H.M., Tong W.Y., Yeung K.W.K., Tjong S.C. (2013). Novel polypropylene biocomposites reinforced with carbon nanotubes and hydroxyapatite nanorods for bone replacements. Mater. Sci. Eng. C.

[53] Liao C., Wong H.M., Yeung K.W.K., Tjong S.C. (2014). The development, fabrication and material characterization of polypropylene composites reinforced with carbon nanofiber and hydroxyapatite nanorod hybrid fillers. Int. J. Nanomed..

[54] Liu C., Wong H.M., Yeung K.W., Tjong S.C. (2016). Novel electrospun polylactic acid nanocomposite fiber mats with hybrid graphene oxide and nanohydroxyapatite reinforcements having enhanced biocompatibility. Polymers.

[55] Dimapilis E.A., Hsu C.S., Mendoza R.M., Lu M.C. (2018). Zinc oxide nanoparticles for water disinfection. Sustain. Environ. Res..

[56] Tian C., Zhang Q., Wu A., Jiang M., Liang Z., Jiang B., Fu H. (2012). Cost-effective large-scale synthesis of ZnO photocatalyst with excellent performance for dye photodegradation. Chem. Commun..

[57] Raji R., Gopchandran K.G. (2017). ZnO nanostructures with tunable visible luminescence: Effects of kinetics of chemical reduction and annealing. J. Sci. Adv. Mater. Dev..

[58] Kumar S.G., Rao K.S. (2015). Zinc oxide based photocatalysis: Tailoring surface-bulk structure and related interfacial charge carrier dynamics for better environmental applications. RSC Adv..

[59] Sang Y., Liu H., Umar A. (2015). Photocatalysis from UV/vis to near-infrared light: Toward full solar-light spectrum activity. ChemCatChem.

[60] Ong C.B., Ng L.Y., Mohammad A.W. (2018). A review of ZnO nanoparticles as solar photocatalysts: Synthesis, mechanisms and applications. Renew. Sustain. Energy Rev..

[61] Saleh R., Djaja N.F. (2014). Transition-metal-doped ZnO nanoparticles: Synthesis, characterization and photocatalytic activity under UV light. Spectrochim. Acta Part A.

[62] Cardoza-Contreras M.N., Vásquez-Gallegos A., Vidal-Limon A., Romo-Herrera J.M., Aguila S., Contreras O.E. (2019). Photocatalytic and antimicrobial properties of Ga doped and Ag doped ZnO nanorods for water treatment. Catalysts.

[63] Naskar A., Lee S., Kim K.S. (2020). Antibacterial potential of Ni-doped zinc oxide nanostructure: Comparatively more effective against Gram-negative bacteria including multidrug resistant strains. RSC Adv..

[64] Azfar A.K., Kasim M.F., Lokman I.M., Rafaie H.A., Mastuli M.S. (2020). Comparative study on photocatalytic activity of transition metals (Ag and Ni)-doped ZnO nanomaterials synthesized via sol–gel method. R. Soc. Open Sci..

[65] Papadaki D., Mhlongo G.H., Motaung D.E., Nkosi S.S., Panagiotaki K., Chrsitaki E., Assimakopoulos M.N., Papadimitriou V.C., Rosei F., Kiriakidis G. (2019). Hierarchically porous Cu-, Co-, and Mn-doped platelet-like ZnO nanostructures and their photocatalytic performance for indoor air quality control. ACS Omega.

[66] Mohammadi-Aloucheh R., Habibi-Yangjeh A., Bayrami A., Latifi-Navid S., Asadi A. (2018). Enhanced anti-bacterial activities of ZnO nanoparticles and ZnO/CuO nanocomposites synthesized using Vaccinium arctostaphylos L. fruit extract. Artif. Cells Nanomed. B.

[67] Liao C., Jin Y., Li Y., Tjong S.C. (2020). Interactions of ZnO nanostructures with mammalian cells: Cytotoxicity and photocatalytic toxicity. Int. J. Mol. Sci..

[68] Wang Q., Li S., He Q., Zhu W., He D., Peng F., Lei L., Zhang L., Zhang Q., Tan L. (2018). Reciprocating compression of ZnO probed by X-ray diffraction: The size efect on structural properties under high pressure. Inorg. Chem..

[69] Yan X., Dong H., Li Y., Lin C., Park C., He D., Yang W. (2016). Phase transition induced strain in ZnO under high pressure. Sci. Rep..

[70] Razavi-Khosroshahi H., Edalati K., Wu J., Nakashima Y., Arita M., Ikoma Y., Sadakiyo M., Inagaki Y., Staykov A., Yamauchi M. (2017). High-pressure zinc oxide phase as visible-light-active photocatalyst with narrow band gap. J. Mater. Chem. A.

[71] Hidalgo-Jimenez J., Wang Q., Edalati K., Cubero-Sesin J.M., Razavi-Khosroshahi H., Ikoma Y., Gutierrez-Fallas D., Dittel-Meza F.A., Rodriguez-Rufino J.C., Fuji M. (2020). Phase transformations, vacancy formation and variations of optical and photocatalytic properties in TiO_2_-ZnO composites by high-pressure torsion. Int. J. Plast..

[72] Ozgur U., Alivov Y.I., Liu C., Teke A., Reshchikov M.A., Doganl S., Avrutin V., Cho J.S., Morkoc H. (2005). A comprehensive review of ZnO materials and devices. J. Appl. Phys..

[73] Kamble A.S., Sinha B.B., Chung K., Gil M.G., Burungale V., Park C.J., Kim J.H., Patil P.S. (2014). Effect of hydroxide anion generating agents on growth and properties of ZnO nanorod arrays. Electrochim. Acta.

[74] Wang Z.L. (2004). Nanostructures of zinc oxide. Mater. Today.

[75] Mora-Fonz D., Lazauskas T., Farrow M.R., Catlow R.A., Woodley S.M., Sokol A.A. (2017). Why are polar surfaces of ZnO stable?. Chem. Mater..

[76] Rana A.U., Lee J.Y., Shahid A., Kim H.-S. (2017). Growth method-dependent and defect density-oriented structural, optical, conductive, and physical properties of solution-grown ZnO nanostructures. Nanomaterials.

[77] Araujo E.A., Nobre F.X., da Silva Sousa G., Cavalcante L.S., Santos M.R., Souza F.L., de Matos J.M. (2017). Synthesis, growth mechanism, optical properties and catalytic activity of ZnO microcrystals obtained via hydrothermal processing. RSC Adv..

[78] Napi M.L., Sultan S.M., Ismail R., How K.W., Ahmad M.K. (2019). Electrochemical-based biosensors on different zinc oxide nanostructures: A review. Materials.

[79] Karnati P., Haque A., Taufique M.F.N., Ghosh K. (2018). A Systematic study on the structural and optical properties of vertically aligned zinc oxide nanorods grown by high pressure assisted pulsed laser deposition technique. Nanomaterials.

[80] Ching K.L., Li G., Ho Y.L., Kwok H.S. (2016). The role of polarity and surface energy in the growth mechanism of ZnO from nanorods to nanotubes. CrystEngComm.

[81] Leelavathi A., Madras G., Ravishankar N. (2013). Origin of enhanced photocatalytic activity and photoconduction in high aspect ratio ZnO nanorods. Phys. Chem. Chem. Phys..

[82] Samadi M., Zirak M., Naseri A., Kheirabadi M., Ebrahimi M., Moshfegh A.Z. (2019). Design and tailoring of one-dimensional ZnO nanomaterials for photocatalytic degradation of organic dyes: A review. Res. Chem. Intermed..

[83] Chaudhary S., Umar A., Bhasin K.K., Baskoutas S. (2018). Chemical sensing applications of ZnO nanomaterials. Materials.

[84] Ishioka J., Kogure K., Ofuji K., Kawaguchi K., Jeem M., Kato T., Shibayama T., Watanabe S. (2017). In situ direct observation of photocorrosion in ZnO crystals in ionic liquid using a laser-equipped high-voltage electron microscope. AIP Adv..

[85] Han J., Qiu W., Gao W. (2010). Potential dissolution and photo-dissolution of ZnO thin films. J. Hazard. Mater..

[86] Zhang L., Jeem M., Okamoto K., Watanabe S. (2018). Photochemistry and the role of light during the submerged photosynthesis of zinc oxide nanorods. Sci. Rep..

[87] Zhang L., Cheng H., Zong R., Zhu Y. (2009). Photocorrosion suppression of ZnO nanoparticles via hybridization with graphite-like carbon and enhanced photocatalytic activity. J. Phys. Chem. C.

[88] Han C., Yang M.Q., Weng B., Xu X.J. (2014). Improving the photocatalytic activity and anti-photocorrosion of semiconductor ZnO by coupling with versatile carbon. Phys. Chem. Chem. Phys..

[89] Peng Y., Ji J., Chen D. (2015). Ultrasound assisted synthesis of ZnO/reduced graphene oxide composites with enhanced photocatalytic activity and anti-photocorrosion. Appl. Surf. Sci..

[90] Zhang Y., Mandal R., Ratchford D.C., Anthony R., Yeom J. (2020). Si nanocrystals/ZnO nanowires hybrid structures as immobilized photocatalysts for photodegradation. Nanomaterials.

[91] Rodwihok C., Wongratanaphisan D., Ngo Y.L., Khandelwal M., Hur S.H., Chung J.S. (2019). Effect of GO additive in ZnO/rGO nanocomposites with enhanced photosensitivity and photocatalytic activity. Nanomaterials.

[92] Taylor C.M., Ramirez-Canon A., Wenk J., Mattia D. (2019). Enhancing the photo-corrosion resistance of ZnO nanowire photocatalysts. J. Hazard. Mater..

[93] Zhang Q., Xu M., You B., Zhang Q., Yuan H., Ostrikov K. (2018). Oxygen vacancy-mediated ZnO nanoparticle photocatalyst for degradation of methylene blue. Appl. Sci..

[94] Ansari S.A., Khan M.M., Kalathil S., Nisar A., Lee J., Cho M.H. (2013). Oxygen vacancy induced band gap narrowing of ZnO nanostructures by an electrochemically active biofilm. Nanoscale.

[95] Tang Y., Zhou H., Zhang K., Ding J., Fan T., Zhang D. (2015). Visible-light-active ZnO via oxygen vacancy manipulation for efficient formaldehyde photodegradation. Chem. Eng. J..

[96] Dash P., Manna A., Mishra N.C., Varma S. (2019). Synthesis and characterization of aligned ZnO nanorods for visible light photocatalysis. Physica E.

[97] Gupta J., Bahadur D. (2018). Defect-mediated reactive oxygen species generation in Mg-substituted ZnO nanoparticles: Efficient nanomaterials for bacterial inhibition and cancer therapy. ACS Omega.

[98] Mia M.N.H., Pervez M.F., Hossain M.K., Rahman M.R., Uddin M.J., Al Mashud M.A., Ghosh H.K., Hoq M. (2017). Influence of Mg content on tailoring optical bandgap of Mg-doped ZnO thin film prepared by sol-gel method. Results Phys..

[99] Kasi G., Seo J. (2019). Influence of Mg doping on the structural, morphological, optical, thermal, and visible-light responsive antibacterial properties of ZnO nanoparticles synthesized via co-precipitation. Mater. Sci. Eng. C.

[100] Ma Z., Ren F., Ming X., Long Y., Volinsky A.A. (2019). Cu-doped ZnO electronic structure and optical properties studied by first-principles calculations and experiments. Materials.

[101] Modwi A., Ghanem M.A., Al-Mayouf A.M., Houas M. (2018). Lowering energy band gap and enhancing photocatalytic properties of Cu/ZnO composite decorated by transition metals. J. Mol. Struct..

[102] Gupta J., Bahadur D. (2017). Visible light sensitive mesoporous Cu-substituted ZnO nano assembly for enhanced photocatalysis, bacterial inhibition, and noninvasive tumor regression,. ACS Sustain. Chem. Eng..

[103] Bhuyan T., Sharma R., Anand S. (2015). A comparative study of pure and copper (Cu)-doped ZnO nanorods for antibacterial and photocatalytic applications with their mechanism of action. J. Nanopart. Res..

[104] Rajivgandhi G.N., Ramachandran G., Alharbi N.S., Kadaikunnan S., Khaleed J.M., Manokaran N., Li W.J. (2021). Substantial effect of Cr doping on the antimicrobial activity of ZnO nanoparticles prepared by ultrasonication process. Mater. Sci. Eng. B.

[105] Jacob N.M., Madras G., Kottam N., Thomas T. (2014). Multivalent Cu-doped ZnO nanoparticles with full solar spectrum absorbance and enhanced photoactivity. Ind. Eng. Chem. Res..

[106] Tsuzuki T., He R., Dodd A., Saunders M. (2019). Challenges in determining the location of dopants, to study the influence of metal doping on the photocatalytic activities of ZnO nanopowders. Nanomaterials.

[107] Ma Q., Lv X., Wang Y., Chen J. (2016). Optical and photocatalytic properties of Mn doped flower-like ZnO hierarchical structures. Opt. Mater..

[108] Li W., Wang G., Chen C., Liao J., Li Z. (2017). Enhanced visible light photocatalytic activity of ZnO nanowires doped with Mn^2+^ and Co^2+^ ions. Nanomaterials.

[109] Achouri F., Corbel S., Balan L., Mozet K., Girot E., Medjahdi G., Said M.B., Ghrabi A., Schneider R. (2016). Porous Mn-doped ZnO nanoparticles for enhanced solar and visible light photocatalysis. Mater. Des..

[110] Han X., Wahl S., Russo P.A., Pinna N. (2018). Cobalt-assisted morphology and assembly control of Co-doped ZnO nanoparticles. Nanomaterials.

[111] Yin Q., Qiao R., Li Z., Zhang X.L., Zhu L. (2015). Hierarchical nanostructures of nickel-doped zinc oxide: Morphology controlled synthesis and enhanced visible-light photocatalytic activity. J. Alloy Compd..

[112] Singh P., Kumar R., Singh R.K. (2019). Progress on transition metal-doped ZnO nanoparticles and its application. Ind. Eng. Chem. Res..

[113] Bora T., Zoepfl D., Dutta J. (2016). Importance of plasmonic heating on visible light driven photocatalysis of gold nanoparticle decorated zinc oxide nanorods. Sci. Rep..

[114] Sarma B., Sarma B.K. (2017). Fabrication of Ag/ZnO heterostructure and the role of surface coverage of ZnO microrods by Ag nanoparticles on the photophysical and photocatalytic properties of the metal-semiconductor system. Appl. Surf. Sci..

[115] Liu Q., Liu E., Li J., Qiu Y., Chen R. (2020). Rapid ultrasonic-microwave assisted synthesis of spindle-like Ag/ZnO nanostructures and their enhanced visible-light photocatalytic and antibacterial activities. Catal. Today.

[116] Lyadov N.M., Gumarov A.I., Kashapov R.N., Noskov A.I., Valeev V.F., Nuzhdin V.I., Bazarov V.V., Khaibullin R.I., Faizrakhmanov I.A. (2016). Structure and optical properties of ZnO with silver nanoparticles. Semiconductors.

[117] Liu H., Hu Y., Zhang Z., Liu Z., Jia H., Xu B. (2015). Synthesis of spherical Ag/ZnO heterostructural composites with excellent photocatalytic activity under visible light and UV irradiation. Appl. Surf. Sci..

[118] Raji R., Sibi K.S., Gopchandran K.G. (2018). ZnO: Ag nanorods as efficient photocatalysts: Sunlight driven photocatalytic degradation of sulforhodamine B. Appl. Surf. Sci..

[119] Chamorro W., Ghanbaja J., Battie Y., Naciri A.E., Soldera F., Mücklich F., Horwat D. (2016). Local structure-driven localized surface plasmon absorption and enhanced photoluminescence in ZnO-Au thin films. J. Phys. Chem. C.

[120] Zhang J., Tse K., Wong M., Zhang Y., Zhu J. (2016). A brief review of co-doping. Front. Phys..

[121] Yan F., Wang Y., Zhang J., Lin Z. (2014). Schottky or Ohmic metal–semiconductor contact: Influence on photocatalytic efficiency of Ag/ZnO and Pt/ZnO model systems. ChemSusChem.

[122] Clavero C. (2014). Plasmon-induced hot-electron generation at nanoparticle/metal-oxide interfaces for photovoltaic and photocatalytic devices. Nat. Photonics.

[123] Furube A., Hashimoto S. (2017). Insight into plasmonic hot-electron transfer and plasmon molecular drive: New dimensions in energy conversion and nanofabrication. NPG Asia Mater..

[124] Krajczewski J., Kolataj K., Kudelski A. (2017). Plasmonic nanoparticles in chemical analysis. RSC Adv..

[125] Wang C.S., Lin H.Y., Lin J.M., Chen Y.F. (2012). Surface-plasmon-enhanced ultraviolet random lasing from ZnO nanowires assisted by Pt nanoparticles. Appl. Phys. Express.

[126] Pei J., Jiang D., Zhao M., Duan Q., Liu R., Sun L., Guo Z., Hou J., Qin J., Li B. (2016). Controlled enhancement range of the responsivity in ZnO ultraviolet photodetectors by Pt nanoparticles. Appl. Surf. Sci..

[127] Fageria P., Gangopadhyay S., Pande S. (2014). Synthesis of ZnO/Au and ZnO/Ag nanoparticles and their photocatalytic application using UV and visible light. RSC Adv..

[128] Ziashahabi A., Prato M., Dang Z., Poursalehi R., Naseri N. (2019). The effect of silver oxidation on the photocatalytic activity of Ag/ZnO hybrid plasmonic/metal-oxide nanostructures under visible light and in the dark. Sci. Rep..

[129] Zhang L., Zhu X., Wang Z., Yun S., Guo T., Zhang J., Hu T., Jiang J., Chen J. (2019). Synthesis of ZnO doped high valence S element and study of photogenerated charges properties. RSC Adv..

[130] Zhang X., Qin J., Hao R., Wang L., Shen X., Yu R., Limpanart S., Ma M., Liu R. (2015). Carbon-doped ZnO nanostructures: Facile synthesis and visible light photocatalytic applications. J. Phys. Chem. C.

[131] Lavand A.B., Malghe Y.S. (2015). Synthesis, characterization, and visible light photocatalytic activity of nanosized carbon doped zinc oxide. Int. J. Photochem..

[132] Gionco C., Fabbri D., Calza P., Paganini M.C. (2016). Photocatalytic tests of N-doped zinc oxide: A New interesting photocatalyst. J. Nanometer..

[133] Kumari R., Sahai A., Goswami N. (2015). Effect of nitrogen doping on structural and optical properties of ZnO nanoparticles. Prog. Nat. Sci-Mater..

[134] Lavand A.B., Malghe Y.S. (2015). Synthesis, characterization and visible light photocatalytic activity of nitrogen-doped zinc oxide nanospheres. J. Asian Ceram. Soc..

[135] Gupta R., Eswar N.K., Modak J.M., Madras G. (2016). Visible light driven efficient N and Cu co-doped ZnO for photoinactivation of Escherichia coli. RSC Adv..

[136] Wang Y., Cheng J., Yu S., Alcocer E.J., Shahid M., Wang Z., Pan W. (2016). Synergistic effect of N-decorated and Mn^2+^ doped ZnO nanofibers with enhanced photocatalytic activity. Sci. Rep..

[137] Georgakilas V., Perman J.A., Tucek J., Zboril R. (2015). Broad family of carbon nanoallotropes: Classification, chemistry, and applications of fullerenes, carbon dots, nanotubes, graphene, nanodiamonds, and combined superstructures. Chem. Rev..

[138] Nair R.R., Blake P., Grigorenko A.N., Novoselov K.S., Booth T.J., Stauber T., Peres N.M., Geim A.K. (2008). Fines structure constant defines visual transparency of graphene. Science.

[139] Hu L., Hecht D.S., Grüner G. (2010). Carbon nanotube thin films: Fabrication, properties, and applications. Chem. Rev..

[140] Kim H., Wang M., Lee S.K., Kang J., Nam J.D., Ci L., Suhr J. (2017). Tensile properties of millimeter-long multi-walled carbon nanotubes. Sci. Rep..

[141] Kumar P., Huo P., Zhang R., Liu B. (2019). Antibacterial properties of graphene-based nanomaterials. Nanomaterials.

[142] Karahan H.E., Wiraja C., Xu C., Wei J., Wang Y., Wang L., Liu F., Chen Y. (2018). Graphene materials in antimicrobial nanomedicine: Current status and future perspectives. Adv. Healthc. Mater..

[143] Al-Jumaili A., Alancherry S., Bazaka K., Jacob M.V. (2017). Review on the antimicrobial properties of carbon nanostructures. Materials.

[144] He L., Tjong S.C. (2016). Nanostructured transparent conductive films: Fabrication, characterization and applications. Mater. Sci. Eng. R Rep..

[145] He L., Tjong S.C. (2016). Aqueous graphene oxide-dispersed carbon nanotubes as inks for the scalable production of all-carbon transparent conductive films. J. Mater. Chem. C.

[146] He L., Liao C., Tjong S.C. (2018). Scalable fabrication of high-performance transparent conductors using graphene oxide-stabilized single-walled carbon nanotube inks. Nanomaterials.

[147] Ma Y., Zhi L. (2019). Graphene-based transparent conductive films: Material systems, preparation and applications. Small Methods.

[148] He L., Tjong S.C. (2017). Silver-decorated reduced graphene oxides as novel building blocks for transparent conductive films. RSC Adv..

[149] He L., Tjong S.C. (2013). Low percolation threshold of graphene/polymer composites prepared by solvothermal reduction of graphene oxide in the polymer solution. Nanoscale Res. Lett..

[150] Tjong S.C. (2011). Polymer nanocomposite bipolar plates reinforced with carbon nanotubes and graphite nanosheets. Energy Environ. Sci..

[151] Albero J., Mateo D., Garcia H. (2019). Graphene-based materials as efficient photocatalysts for water splitting. Molecules.

[152] Khazi-Syed A., Hasan M.T., Campbell E., Gonzalez-Rodriguez R., Naumov A.V. (2019). Single-walled carbon nanotube-assisted antibiotic delivery and imaging in S. epidermidis strains addressing antibiotic resistance. Nanomaterials.

[153] Bellamkonda S., Thangavel S., Hafeez H.Y., Neppolian B., Ranga Rao G. (2019). Highly active and stable multi-walled carbon nanotubes-graphene-TiO_2_ nanohybrid: An efficient non-noble metal photocatalyst for water splitting. Catal. Today.

[154] Rauwel P., Galeckas A., Ducroquet F., Rauwel E. (2019). Selective photocurrent generation in HfO_2_ and carbon nanotube hybrid nanocomposites under ultra-violet and visible photoexcitations. Mater. Lett..

[155] Bobrinetskiy A.I., Knezevic N.Z. (2018). Graphene-based biosensors for on-site detection of contaminants in food. Anal. Methods.

[156] Peña-Bahamonde J., Nguyen H.N., Fanouraki S.K., Rodriques D.F. (2018). Recent advances in graphene-based biosensor technology with applications in life sciences. J. Nanobiotechnol..

[157] Campbell E., Hasan M.T., Pho C., Callaghan K., Naumov A.V. (2019). Graphene oxide as a multifunctional platform for intracellular delivery, imaging, and cancer sensing. Sci. Rep..

[158] Maiti D., Tong X., Mou X., Yang K. (2019). Carbon-based nanomaterials for biomedical applications: A recent study. Front. Pharmacol..

[159] Plachá D., Jampilek J. (2019). Graphenic materials for biomedical applications. Nanomaterials.

[160] Mohamed R.M., Shawky A. (2018). CNT supported Mn-doped ZnO nanoparticles: Simple synthesis and improved photocatalytic activity for degradation of malachite green dye under visible light. Appl. Nanosci..

[161] Tie W., Bhattacharyya S.S., Wang Y., He W., Lee S.H. (2017). Facile in-situ synthesis of a zinc oxide crystals/few-layered graphene flake composite for enhanced photocatalytic performance. J. Photochem. Photobiol. A.

[162] Wang F., Zhou Y., Pan X., Lu B., Huang J., Ye Z. (2018). Enhanced photocatalytic properties of ZnO nanorods by electrostatic self-assembly with reduced graphene oxide. Phys. Chem. Chem. Phys..

[163] Polat E.O., Balci O., Kakenov N., Uzlu H.B., Kocabas C., Dahiya R. (2015). Synthesis of large area graphene for high performance in flexible optoelectronic devices. Sci. Rep..

[164] Moreno-Bárcenas A., Perez-Robles J.F., Vorobiev Y.V., Ornelas-Soto N., Mexicano A., García A.G. (2018). Graphene synthesis using a CVD reactor and a discontinuous feed of gas precursor at atmospheric pressure. J. Nanomater..

[165] Chen M., Haddon R.C., Yan R., Bekyarova E. (2017). Advances in transferring chemical vapor deposition graphene: A review. Mater. Horiz..

[166] Knapp M., Hoffmann R., Cimalla V., Ambacher O. (2017). Wettability investigations and wet transfer enhancement of large-area CVD-graphene on aluminum nitride. Nanomaterials.

[167] Guerrero-Contreras J., Caballero-Briones F. (2015). Graphene oxide powders with different oxidation degree, prepared by synthesis variations of the Hummers method. Mater. Chem. Phys..

[168] Dreyer D.R., Park S., Bielawski C.W., Ruoff R.S. (2010). The chemistry of graphene oxide. Chem. Soc. Rev..

[169] Park S., An J., Potts J.R., Velamakanni A., Murali S., Ruoff R.S. (2011). Hydrazine-reduction of graphite- and graphene oxide. Carbon.

[170] Dave K., Park K.H., Dhayal M. (2015). Two-step process for programmable removal of oxygen functionalities of graphene oxide: Functional, structural and electrical characteristics. RSC. Adv..

[171] Hayes W.I., Joseph P., Mughal M.Z., Papakonstantinou P. (2015). Production of reduced graphene oxide via hydrothermal reduction in an aqueous sulfuric acid suspension and its electrochemical behavior. J. Solid State Electrochem..

[172] Pan X., Yang M.Q., Xu Y.J. (2014). Morphology control, defect engineering and photoactivity tuning of ZnO crystals by graphene oxide—A unique 2D macromolecular surfactant. Phys. Chem. Chem. Phys..

[173] Wu D., An T., Li G., Wang W., Cai Y., Yip H.Y., Zhao H., Wong P.K. (2015). Mechanistic study of the visible-light-driven photocatalytic inactivation of bacteria by graphene oxide–zinc oxide composite. Appl. Surf. Sci..

[174] Osorio A.G., Silveira I.C., Bueno V.L., Bergmann C.P. (2008). H_2_SO_4_/HNO_3_/HCl—Functionalization and its effect on dispersion of carbon nanotubes in aqueous media. Appl. Surf. Sci..

[175] Chaudhary D., Singh S., Vankar V.D., Khare N. (2018). ZnO nanoparticles decorated multi-walled carbon nanotubes for enhanced photocatalytic and photoelectrochemical water splitting. J. Photochem. Photobiol. A.

[176] Ganose A.M., Scanlon D.O. (2016). Band gap and work function tailoring of SnO_2_ for improved transparent conducting ability in photovoltaics. J. Mater. Chem. C..

[177] Hamrouni A., Moussa N., Parrino F., Di Paola A., Houas A., Parmisano L. (2014). Sol–gel synthesis and photocatalytic activity of ZnO–SnO_2_ nanocomposites. J. Mol. Catal. A Chem..

[178] Xie W., Li R., Xu Q. (2018). Enhanced photocatalytic activity of Se-doped TiO_2_ under visible light irradiation. Sci. Rep..

[179] Kang X., Liu S., Dai Z., He Y., Song X., Tan Z. (2019). Titanium dioxide: From engineering to applications. Catalysts.

[180] Zhang F., Wang X., Liu H., Liu C., Wan Y., Long Y., Cai Z. (2019). Recent advances and applications of semiconductor photocatalytic technology. Appl. Sci..

[181] Maya-Trevino M.L., Guzman-Mar J.L., Hinojosa-Reyes L., Ramos-Delgado N.A., Maldonado M.I., Hernandez-Ramirez A. (2014). Activity of the ZnO–Fe_2_O_3_ catalyst on the degradation of Dicamba and 2,4-D herbicides using simulated solar light. Ceram. Int..

[182] Guo L., Wang Y., He T. (2016). Photocatalytic reduction of CO_2_ over heterostructure semiconductors into value added chemicals. Chem. Rec..

[183] Moniz S.J., Shevlin S.A., Martin D.J., Guo Z.X., Tang J. (2015). Visible-light driven heterojunction photocatalysts for water splitting—A critical review. Energy Environ. Sci..

[184] Wang Y., Wang Q., Zhan X., Wang F., Safdar M., He J. (2013). Visible light driven type II heterostructures and their enhanced photocatalysis properties: A review. Nanoscale.

[185] Sakib A.A., Masum S.M., Hinkis J., Islam R., Molla M.A. (2019). Synthesis of CuO/ZnO nanocomposites and their application in photodegradation of toxic textile dye. J. Compos. Sci..

[186] Isac L., Cazan C., Enesca A., Andronic L. (2019). Copper sulfide based heterojunctions as photocatalysts for dyes photodegradation. Front. Chem..

[187] Kołodziejczak-Radzimska A., Jesionowski T. (2014). Zinc oxide–from synthesis to application: A review. Materials.

[188] Hanif M.A., Lee I., Akter J., Islam M.A., Zahid A.A., Sapkota K.P., Hahn J.R. (2019). Enhanced Photocatalytic and antibacterial performance of ZnO nanoparticles prepared by an efficient thermolysis method. Catalysts.

[189] Baptista A., Silva F., Porteiro J., Míguez J., Pinto G. (2018). Sputtering physical vapour deposition (PVD) coatings: A critical review on process improvement and market trend demands. Coatings.

[190] Laurenti M., Cuda V. (2018). Porous zinc oxide thin Films: Synthesis approaches and applications. Coatings.

[191] Wen X., Zhang Q., Shao Z. (2019). Magnetron sputtering for ZnO:Ga scintillation film production and its application research status in nuclear detection. Crystals.

[192] Kim M., Osone S., Kim T., Higashi H., Seto T. (2017). Synthesis of nanoparticles by laser ablation: A review. Kona Powder Part J..

[193] Wisz G., Virt I., Sagan P., Potera P., Yavorskyi R. (2017). Structural, optical and electrical properties of zinc oxide layers produced by pulsed laser deposition method. Nanoscale Res. Lett..

[194] Luo C.Q., Ling F.C., Rahman M.A., Phillips M., Ton-That C., Liao C., Shih K., Lin J., Tam H.W., Djurisic A.B. (2019). Surface polarity control in ZnO films deposited by pulsed laser deposition. Appl. Surf. Sci..

[195] Kaassamani S., Kassem W., Tabbal M. (2019). X-ray diffraction lineshape analysis of pulsed laser deposited ZnO nano-structured thin films. Appl. Surf. Sci..

[196] Sportelli M.C., Izzi M., Volpe A., Clemente M., Picca R.A., Ancona A., Lugarà P.M., Palazzo G., Cioffi N. (2018). The pros and cons of the use of laser ablation synthesis for the production of silver nano-antimicrobials. Antibiotics.

[197] Ostrowski R., Barcikowski S., Marczak J., Ostendorf A., Strzelec M., Walter J., Nimmrichter J., Kautek W., Schreiner M. (2007). Health risks caused by particulate emission during laser cleaning. Lasers in the Conservation of Artworks, Springer Proceedings in Physics, Madrid, Spain, 17–21 September 2007.

[198] Rauwel E., Willinger M.G., Ducroquet F., Rauwel P., Matko I., Kiselev D., Pinna N. (2008). Carboxylic acids as oxygen sources for the atomic layer deposition of high-κ metal oxides. J. Phys. Chem. C.

[199] Lu Y., Hsiech C., Su G. (2019). The role of ALD-ZnO seed layers in the growth of ZnO nanorods for hydrogen sensing. Micromachines.

[200] Laube J., Nübling D., Beh H., Gutsch S., Hiller D., Zacharias M. (2016). Resistivity of atomic layer deposition grown ZnO: The influence of deposition temperature and post-annealing. Thin Solid Films.

[201] Lee B.J., Jo S.I., Jeong G.H. (2019). Synthesis of ZnO nanomaterials using low-cost compressed air as microwave plasma gas at atmospheric pressure. Nanomaterials.

[202] Yang P., Yan H., Mao S., Russo R., Johnson T., Saykally R., Morris N., Pham J., He R., Cho H.J. (2002). Controlled growth of ZnO nanowires and their optical properties. Adv. Funct. Mater..

[203] Wan H., Ruda H. (2010). A study of the growth mechanism of CVD-grown ZnO nanowires. J. Mater. Sci. Mater. Electron..

[204] Tang C., Spencer M.J., Barnard A.S. (2014). Activity of ZnO polar surfaces: An insight from surface energies. Phys. Chem. Chem. Phys..

[205] Gao P.X., Ding Y., Wang Z.L. (2003). Crystallographic orientation-aligned ZnO nanorods grown by a tin catalyst. Nano Lett..

[206] Zhao X., Shaymurat T., Pei T., Bai L., Cai B., Tong Y., Tang Q., Liu Y. (2012). Low-temperature, catalyst-free vapor–solid growth of ultralong ZnO nanowires. Mater. Chem. Phys..

[207] Hedrich C., Haugg S., Pacarizi L., Furlan K.P., Blick R.H., Zierold R. (2019). Low-temperature vapor-solid growth of ZnO nanowhiskers for electron field emission. Coatings.

[208] Ding Y., Gao P.X., Wang Z.L. (2004). Catalyst−nanostructure interfacial lattice mismatch in determining the shape of VLS grown nanowires and nanobelts: A case of Sn/ZnO. J. Am. Chem. Soc..

[209] Kong X.Y., Wang Z.L. (2003). Spontaneous polarization-induced nanohelixes, nanosprings, and nanorings of piezoelectric nanobelts. Nano Lett..

[210] Uekawa N., Yamashita R., Wu Y.J., Kakegawa K. (2004). Effect of alkali metal hydroxide on formation processes of zinc oxide crystallites from aqueous solutions containing Zn(OH)_4_^2−^ ions. Phys. Chem. Chem. Phys..

[211] He G., Huang B., Lin Z., Yang W., He Q., Li L. (2018). Morphology transition of ZnO nanorod arrays synthesized by a two-step aqueous solution method. Crystals.

[212] Akhtar M.J., Ahamed M., Kumar S., Khan M.M., Ahmad J., Alrokayan S.A. (2012). Zinc oxide nanoparticles selectively induce apoptosis in human cancer cells through reactive oxygen species. Int. J. Nanomed..

[213] Cao D., Gong S., Shu X., Zhu D., Liang S. (2019). Preparation of ZnO nanoparticles with high dispersibility based on oriented attachment (OA) process. Nanoscale Res. Lett..

[214] Ali A., Ambreen S., Javed R., Tabassum S., Haq I.U., Zia M. (2017). ZnO nanostructure fabrication in different solvents transforms physio-chemical, biological and photodegradable properties. Mater. Sci. Eng. C.

[215] Pourrahimi A.M., Liu D., Pallon L.K., Andersson R.L., Abad A.M., Lagaron J.M., Hedenqvist M.S., Strom V., Gedde U.W., Olsson R.T. (2014). Water-based synthesis and cleaning methods for high purity ZnO nanoparticles—Comparing acetate, chloride, sulphate and nitrate zinc salt precursors. RSC Adv..

[216] Nithya K., Kalyanasundharam S. (2019). Effect of chemically synthesis compared to biosynthesized ZnO nanoparticles using aqueous extract of C. halicacabum and their antibacterial activity. OpenNano.

[217] Kumar B., Smita K., Cumbal L., Debut A. (2014). Green approach for fabrication and applications of zinc oxide nanoparticles. Bioinorg. Chem. Appl..

[218] Nava O.J., Soto-Robles C.A., Gómez-Gutiérrez C.M., Vilchis-Nestor A.R., Castro-Beltrán A., Olivas A., Luque P.A. (2017). Fruit peel extract mediated green synthesis of zinc oxide nanoparticles. J. Mol. Struct..

[219] Thi T.U., Nguyen T.T., Thi Y.D., Thi K.H., Phan B.T., Pham K.N. (2020). Green synthesis of ZnO nanoparticles using orange fruit peel extract for antibacterial activities. RSC Adv..

[220] Rupa E.J., Kaliraj L., Abid S., Yang D.C., Jung S.K. (2019). Synthesis of a zinc oxide nanoflower photocatalyst from sea buckthorn fruit for degradation of industrial dyes in wastewater treatment. Nanomaterials.

[221] Liu Z., Ya J., Lei E. (2010). Effects of substrates and seed layers on solution growing ZnO nanorods. J. Solid State Electrochem..

[222] Tlemcani T.S., Justeau C., Nadaud K., Poulin-Vittrant G., Alquier D. (2019). Deposition time and annealing effects of ZnO seed layer on enhancing vertical alignment of piezoelectric ZnO nanowires. Chemosensors.

[223] Karim S.S., Takamura Y., Tue P.T., Tung N.T., Kazmi J., Dee C.F., Majlis B.Y., Mohamed M.A. (2020). Developing conductive highly ordered zinc oxide nanorods by acetylacetonate-assisted growth. Materials.

[224] Matei A., Dumitrescu L., Cernica I., Tucureanu V., Mihalache I., Bita B., Danila M., Manciulea I. (2015). Study of the influence of capping agents on the structural and optical properties of ZnO nanostructures. J. Optoelectron. Adv. M.

[225] Ramimoghadam D., Hussein M.Z., Taufiq-Yap Y.H. (2012). The effect of sodium dodecyl sulfate (SDS) and cetyltrimethylammonium bromide (CTAB) on the properties of ZnO synthesized by hydrothermal method. Int. J. Mol. Sci..

[226] Thilagavathi T., Geetha D. (2014). Nano ZnO structures synthesized in presence of anionic and cationic surfactant under hydrothermal process. Appl. Nanosci..

[227] Zhang Y., Newton B., Lewis E., Fu P.P., Kafoury R., Ray P.C., Yu H. (2015). Cytotoxicity of organic surface coating agents used for nanoparticles synthesis and stability. Toxicol Vitr..

[228] Fukui H., Iwahashi H., Nishio K., Hagihara Y., Yoshida Y., Horie M. (2017). Ascorbic acid prevents zinc oxide nanoparticle—Induced intracellular oxidative stress and inflammatory responses. Toxicol. Ind. Health.

[229] Hossain A., Abdalla Y., Ali M.A., Masum M.M., Li B., Sun G., Meng Y., Wang Y., An Q. (2019). Lemon-fruit-based green synthesis of zinc oxide nanoparticles and titanium dioxide nanoparticles against soft rot bacterial pathogen Dickeya dadantii. Biomolecules.

[230] Dong J., Wu J., Hao H., Xing J., Liu H., Gao H. (2017). Synthesis of ZnO nanocrystals and application in inverted polymer solar cells. Nanoscale Res. Lett..

[231] Qiu J., Weng B., Zhao L., Chang C., Shi Z., Li X., Kim H.K., Hwang Y.H. (2014). Synthesis and characterization of flower-like bundles of ZnO nanosheets by a surfactant-free hydrothermal process. J. Nanomater..

[232] Napi M.L., Sultan S.M., Ismail R., Ahmad M.K., Chai G.M. (2019). Optimization of a hydrothermal growth process for low resistance 1D fluorine-doped zinc oxide nanostructures. J. Nanomater..

[233] Zhou Q., Wen J.Z., Zhao P., Anderson W.A. (2017). Synthesis of vertically-aligned zinc oxide nanowires and their application as a photocatalyst. Nanomaterials.

[234] Zare M., Namratha K., Byrappa K., Surendra D.M., Yallappa S., Hungund B. (2018). Surfactant assisted solvothermal synthesis of ZnO nanoparticles and study of their antimicrobial and antioxidant properties. J. Mater. Sci. Technol..

[235] Agnihotri S., Bajaj G., Mukherji S., Mukherji S. (2015). Arginine-assisted immobilization of silver nanoparticles on ZnO nanorods: An enhanced and reusable antibacterial substrate without human cell cytotoxicity. Nanoscale.

[236] Garino N., Limongi T., Dumontel B., Canta M., Racca L., Laurenti M., Castellino M., Casu A., Falqui A., Cauda V. (2019). A microwave-assisted synthesis of zinc oxide nanocrystals finely tuned for biological applications. Nanomaterials.

[237] Wojnarowicz J., Chudoba T., Gierlotka S., Lojkowski W. (2018). Effect of microwave radiation power on the size of aggregates of ZnO NPs prepared using microwave solvothermal synthesis. Nanomaterials.

[238] Sounart T.L., Liu J., Voight J.A., Hoe M., Spoerke E.D., McKenzie B. (2007). Secondary nucleation and growth of ZnO. J. Am. Chem. Soc..

[239] Pung S.Y., Lee W.P., Aziz A. (2012). Kinetic study of organic dye degradation using ZnO particles with different morphologies as a photocatalyst. Int. J. Inorg. Chem..

[240] Zhong L., Yun K. (2015). Graphene oxide-modified ZnO particles: Synthesis, characterization, and antibacterial properties. Int. J. Nanomed..

[241] Tuan P.V., Phuong T.T., Tan V.T., Nguyen S.X., Khiem N. (2020). In-situ hydrothermal fabrication and photocatalytic behavior of ZnO/reduced graphene oxide nanocomposites with varying graphene oxide concentrations. Mater. Sci. Semicond. Process..

[242] Wang Y.W., Cao A., Jiang Y., Zhang X., Liu J.H., Liu Y., Wang H. (2014). Superior antibacterial activity of zinc oxide/graphene oxide composites originating from high zinc concentration localized around bacteria. ACS Appl. Mater. Interfaces.

[243] Rajveer R.S., Sharma V., Ronin R.S., Gupta D.K., Srivastava S., Agrawal K., Vijay Y.K. (2017). Synthesis, characterization and enhanced antimicrobial activity of reduced graphene oxide-zinc oxide nanocomposite. Mater. Res. Express.

[244] Zhang P., Li Z., Zhang S., Shao G. (2018). Recent advances in effective reduction of graphene oxide for highly improved performance toward electrochemical energy storage. Energy Environ Mater..

[245] Hsueh Y.-H., Hsieh C.-T., Chiu S.-T., Tsai P.-H., Liu C.-Y., Ke W.-J. (2019). Antibacterial property of composites of reduced graphene oxide with nano-silver and zinc oxide nanoparticles synthesized using a microwave-assisted approach. Int. J. Mol. Sci..

[246] Prema D., Prakash J., Vignesh S., Veluchamy P., Ramachandran C., Samal D.B., Oh D.H., Sahabudeen S., Venkatasubbu G.D. (2020). Mechanism of inhibition of graphene oxide/zinc oxide nanocomposite against wound infection causing pathogens. Appl. Nanosci..

[247] Khan M.F., Ansari A.H., Hameedullah M., Ahmad E., Husain F.M., Zia Q., Baig U., Zaheer M., Alam M.M., Khan A.M. (2016). Sol-gel synthesis of thorn-like ZnO nanoparticles endorsing mechanical stirring effect and their antimicrobial activities: Potential role as nano-antibiotics. Sci. Rep..

[248] Danks A.E., Hall S.R., Schnepp Z. (2016). The evolution of ‘sol–gel’ chemistry as a technique for materials synthesis. Mater. Horiz..

[249] Iannaccone G., Bernardi A., Suriano R., Bianchi C.L., Levi M., Turri S., Griffini G. (2016). The role of sol–gel chemistry in the low temperature formation of ZnO buffer layers for polymer solar cells with improved performance. RSC Adv..

[250] Deshmukh R., Niederberger M. (2017). Mechanistic aspects in the formation, growth and surface functionalization of metal oxide nanoparticles in organic solvents. Chem. Eur. J..

[251] Davis K., Yarbrough R., Froeschle M., White J., Rathnayake H. (2019). Band gap engineered zinc oxide nanostructures via a sol–gel synthesis of solvent driven shape controlled crystal growth. RSC Adv..

[252] Haque M.J., Bellah M.M., Hassan M.R., Rahman S. (2020). Synthesis of ZnO nanoparticles by two different methods & comparison of their structural, antibacterial, photocatalytic and optical properties. Nano Express.

[253] Rodrigues E.S., Silva M.S., Azevedo W.M., Feitosa S.S., Stingl A., Farias P.M. (2019). ZnO nanoparticles with tunable bandgap obtained by modified Pechini method. Appl. Phys. A.

[254] Hingorani S., Pillai V., Kumar P., Multani M.S., Shah D.O. (1993). Microemulsion mediated synthesis of zinc-oxide nanoparticles for varistor studies. Mater. Res. Bull..

[255] Pineda-Reyes A.M., Olvera M. (2018). Synthesis of ZnO nanoparticles from water-in-oil (w/o) microemulsions. Mater. Chem. Phys..

[256] Bumajdad A., Madkour M. (2015). In situ growth of ZnO nanoparticles in precursor-insensitive water-in-oil microemulsion as soft nanoreactors. Nanoscale Res. Lett..

[257] Loh J.H., Samanta A.K., Heng P.W. (2015). Overview of milling techniques for improving the solubility of poorly water-soluble drugs. Asian J. Pharm..

[258] Manzoor U., Siddique S., Ahmed R., Noreen Z., Bokhari H., Ahmad I. (2016). Antibacterial, structural and optical characterization of mechano-chemically prepared ZnO nanoparticles. PLoS ONE.

[259] Soldano G.J., Zanotto F.M., Mariscal M.M. (2016). Mechanochemical stability of sub-nm ZnO chains. Phys. Chem. Chem. Phys..

[260] Arsalani N., Bazazi S., Abuali M., Jodeyri S. (2020). A new method for preparing ZnO/CNT nanocomposites with enhanced photocatalytic degradation of malachite green under visible light. J. Photochem. Photobiol. A.

[261] Mohd Yusof H., Mohamad R., Zaidan U.H., Rahman N.A. (2019). Microbial synthesis of zinc oxide nanoparticles and their potential application as an antimicrobial agent and a feed supplement in animal industry: A review. J. Animal Sci. Biotechnol..

[262] Tiwari V., Mishra N., Gadani K., Solanki P.S., Shah N.A., Tiwari M. (2018). Mechanism of anti-bacterial activity of zinc oxide nanoparticle against carbapenem-resistant Acinetobacter baumannii. Front. Microbiol..

[263] Gold K., Slay B., Knackstedt M., Gaharwar A.K. (2018). Antimicrobial activity of metal and metal-oxide based nanoparticles. Adv. Ther..

[264] Ahmed B., Solanki B., Zaidi A., Khan M.S., Musarrat J. (2019). Bacterial toxicity of biomimetic green zinc oxide nanoantibiotic: Insights into ZnONP uptake and nanocolloid–bacteria interface. Toxicol. Res..

[265] Omar F.M., Aziz H.A., Stoll S. (2014). Stability of ZnO nanoparticles in solution. Influence of pH, dissolution, aggregation and disaggregation effects. J. Colloid Sci. Biotechnol..

[266] Tripathy A., Sen P., Su B., Briscoe W.H. (2017). Natural and bioinspired nanostructured bactericidal surfaces. Adv. Colloid Interface Sci..

[267] Caudill E.R., Hernandez R.T., Johnson K.P., O’Rourke J.T., Zhu L., Haynes C.L., Feng V., Pedersen J.A. (2020). Wall teichoic acids govern cationic gold nanoparticle interaction with Gram-positive bacterial cell walls. Chem. Sci..

[268] Bertani B., Ruiz N. (2018). Function and biogenesis of lipopolysaccharides. EcoSal Plus.

[269] Botos I., Noinaj N., Buchanan S.K. (2017). Insertion of proteins and lipopolysaccharide into the bacterial outer membrane. Philos. Trans. R. Soc. B.

[270] Pace N.J., Weerapana E. (2014). Zinc-binding cysteines: Diverse functions and structural Motifs. Biomolecules.

[271] Ishida T. (2017). Antibacterial mechanism of bacteriolyses of bacterial cell walls by zinc (II) ion induced activations of PGN autolysins, and DNA damages. J. Genes Proteins.

[272] Kadiyala U., Turali-Emre E.S., Bahng J.H., Kotov N.A., VanEpps J.S. (2018). Unexpected insights into antibacterial activity of zinc oxide nanoparticles against methicillin resistant Staphylococcus aureus (MRSA). Nanoscale.

[273] Dutta R.K., Nenavathu B.P., Gangishetty M.K., Reddy A.V. (2012). Studies on antibacterial activity of ZnO nanoparticles by ROS induced lipid peroxidation. Colloids Surf B Biointerfaces.

[274] Stark G. (2005). Functional consequences of oxidative membrane damage. J. Membrane Biol..

[275] Arakha M., Salem M., Mallick B.C., Jha S. (2015). The effects of interfacial potential on antimicrobial propensity of ZnO nanoparticle. Sci. Rep..

[276] Singh R., Cheng S., Singh S. (2020). Oxidative stress-mediated genotoxic effect of zinc oxide nanoparticles on Deinococcus radiodurans. 3 Biotech.

[277] Hirota K., Sugimoto M., Kato M., Tsukagoshi K., Tanigawa T., Sugimoto H. (2010). Preparation of zinc oxide ceramics with a sustainable antibacterial activity under dark conditions. Ceram. Int..

[278] Leung Y., Xu X., Ma A., Liu F., Ng A.M., Shen Z., Gethings L.A., Guo M.Y., Djurisic A.B., Lee P.K.H. (2016). Toxicity of ZnO and TiO_2_ to Escherichia coli cells. Sci. Rep..

[279] Jiang Y., Zhang L., Wen D., Ding Y. (2016). Role of physical and chemical interactions in the antibacterial behavior of ZnO nanoparticles against *E. coli*. Mater. Sci. Eng. C.

[280] Raghupathi K.R., Koodali R.T., Manna A.C. (2011). Size-dependent bacterial growth inhibition and mechanism of antibacterial activity of zinc oxide nanoparticles. Langmuir.

[281] Abbasi B.H., Shah M., Hashmi S.S., Nazir M., Naz S., Ahmad W., Khan I.U., Hano C. (2019). Green bio-assisted synthesis, characterization and biological evaluation of biocompatible ZnO NPs synthesized from different tissues of milk thistle (Silybum marianum). Nanomaterials.

[282] Zare M., Namratha K., Alghamdi S., Mohammad Y.H., Hezam A., Zare M., Drmosh Q.A., Byrappa K., Chandrashekar B.N., Ramakrishna S. (2019). Novel green biomimetic approach for synthesis of ZnO-Ag nanocomposite; antimicrobial activity against food-borne pathogen, biocompatibility and solar photocatalysis. Sci. Rep..

[283] Verma R., Chauhan A., Shandilya M., Li X., Kumar R., Kulshrestha S. (2020). Antimicrobial potential of ag-doped ZnO nanostructure synthesized by the green method using moringa oleifera extract. J. Environ. Chem. Eng..

[284] Zhang H., Chen B., Jiang H., Wang C., Wang H., Wang X. (2011). A strategy for ZnO nanorod mediated multi-mode cancer treatment. Biomaterials.

[285] Chang J.S., Strunk J., Chong M.N., Poh P.E., Ocon J.D. (2020). Multi-dimensional zinc oxide (ZnO) nanoarchitectures as efficient photocatalysts: What is the fundamental factor that determines photoactivity in ZnO?. J. Hazard. Mater..

[286] Hong H., Shi J., Yang Y., Zhang Y., Engle J.W., Nickles R.J., Wang X., Cai W. (2011). Cancer-targeted optical imaging with fluorescent zinc oxide nanowires. Nano Lett..

[287] Sadhukhan P., Kundu M., Rana S., Kumar R., Das J., Sil P.C. (2019). Microwave induced synthesis of ZnO nanorods and their efficacy as a drug carrier with profound anticancer and antibacterial properties. Toxicol. Rep..

[288] Jeong E., Kim C.I., Byun J., Lee J., Kim H.E., Kim E.J., Choi K.J., Hong S.W. (2020). Quantitative evaluation of the antibacterial factors of ZnO nanorod arrays under dark conditions: Physical and chemical effects on Escherichia coli inactivation. Sci. Total Environ..

[289] Li G.R., Hu T., Pan G.L., Yan T.Y., Gao X.P., Zhu H.Y. (2008). Morphology−function relationship of ZnO: Polar planes, oxygen vacancies, and activity. J. Phys. Chem. C.

[290] Tu Y., Chen S., Li X., Gorbaciova J., Gillin W.P., Krause S., Briscoe J. (2018). Control of oxygen vacancies in ZnO nanorods by annealing and their influence on ZnO/PEDOT:PSS diode behavior. J. Mater. Chem. C.

[291] Tam K.H., Cheung C.K., Leung Y.H., Djurisic A.B., Ling C.C., Beling C.D., Fung S., Kwok W.M., Chan W.K., Philips D.L. (2006). Defects in ZnO nanorods prepared by a hydrothermal method. J. Phys. Chem. B.

[292] Cunningham M.W. (2000). Pathogenesis of group A streptococcal infections. Clin. Microbiol. Rev..

[293] Hennigham A., Dohrmann S., Nizet V., Cole J.N. (2015). Mechanisms of group A Streptococcus resistance to reactive oxygen species. FEMS Microbiol. Rev..

[294] Akhavan O., Ghaderi E., Esfandiar A. (2011). Wrapping bacteria by graphene nanosheets for isolation from environment, reactivation by sonication and inactivation by near-infrared irradiation. J. Phys. Chem. B.

[295] Wang D., Zhao L., Ma H., Zhang H., Guo L.H. (2017). Quantitative analysis of reactive oxygen species photogenerated on metal oxide nanoparticles and their bacteria toxicity: The role of superoxide radicals. Environ. Sci. Technol..

[296] Guo B.L., Han P., Guo L.C., Cao Y.Q., Li A.D., Kong J.Z., Zhai H.F., Wu D. (2015). The antibacterial activity of Ta-doped ZnO nanoparticles. Nanoscale Res. Lett..

[297] Vijayalakshmi K., Sivaraj D. (2015). Enhanced antibacterial activity of Cr doped ZnO nanorods synthesized using microwave processing. RSC Adv..

[298] Qi K., Xing X., Zada A., Li M., Wang Q., Liu S.Y., Lin H., Wang G. (2020). Transition metal doped ZnO nanoparticles with enhanced photocatalytic and antibacterial performances: Experimental and DFT studies. Ceram. Int..

[299] Kumar R., Anandan S., Hembram K., Rao T.N. (2014). Efficient ZnO-based visible-light-driven photocatalyst for antibacterial applications. ACS Appl. Mater. Interfaces.

[300] Zhang X., Qin J., Xue Y., Yu P., Zhang B., Wang L., Liu R. (2014). Effect of aspect ratio and surface defects on the photocatalytic activity of ZnO nanorods. Sci. Rep..

[301] Fang J., Fan H., Ma Y., Wang J., Chang Q. (2015). Surface defects control for ZnO nanorods synthesized by quenching and their anti-recombination in photocatalysis. Appl. Surf. Sci..

[302] Prasanna V.K., Vijayaraghavan R. (2017). Chemical manipulation of oxygen vacancy and antibacterial activity in ZnO. Mater. Sci. Eng. C.

[303] Sajjad M., Ullah I., Khan M.I., Khan J., Khan M.Y., Qureshi M.T. (2018). Structural and optical properties of pure and copper doped zinc oxide nanoparticles. Results Phys..

[304] Gupta J., Mohapatra J., Bahadur D. (2017). Visible light driven mesoporous Ag-embedded ZnO nanocomposites: Reactive oxygen species enhanced photocatalysis, bacterial inhibition and photodynamic therapy. Dalton Trans..

[305] He W., Kim H.K., Wamer W.G., Melka D., Callahan J.H., Yin J.J. (2014). Photogenerated charge carriers and reactive oxygen species in ZnO/Au hybrid nanostructures with enhanced photocatalytic and antibacterial activity. J. Am. Chem. Soc..

[306] Das S., Sinha S., Das B., Jayabalan R., Suar M., Mishra A., Tamhankar A.J., Lundborg C.S., Tripathy S.K. (2017). Disinfection of multidrug resistant Escherichia coli by solar-photocatalysis using Fe-doped ZnO nanoparticles. Sci. Rep..

[307] Grotel J., Pikula T., Siedliska K., Ruchomski L., Panek R., Wiertel M., Jartych E. (2018). Structure and hyperfine interactions of Fe-doped ZnO powder prepared by co-precipitation method. Acta Phys. Pol. A.

[308] Cherifi Y., Chaouchi A., Lorgoilloux Y., Rguiti M., Kadri A., Courtois C. (2016). Electrical, dielectric and photocatalytic properties of Fe-doped ZnO nanomaterials synthesized by sol gel method. Process. Appl. Ceram..

[309] Kadi M.W., McKinney D., Mohamed R.M., Mkhalid I.A., Sigmund W. (2016). Fluorine doped zinc oxide nanowires: Enhanced photocatalysts degrade malachite green dye under visible light conditions. Ceram. Int..

[310] Podporska-Carroll J., Myles A., Quilty B., McCormack D.E., Fagan R., Hinder S.J., Dionysiou D.D., Pillai S.C. (2017). Antibacterial properties of F-doped ZnO visible light photocatalyst. J. Hazard. Mater..

[311] Pal S., Maiti S., Maiti U.N., Chattopadhyay K.K. (2015). Low temperature solution processed ZnO/CuO heterojunction photocatalyst for visible light induced photo-degradation of organic pollutants. CrystEngComm.

[312] Mageshwari K., Nataraj D., Pal T., Sathyamoorthy R., Park J. (2015). Improved photocatalytic activity of ZnO coupled CuO nanocomposites synthesized by reflux condensation method. J. Alloys Compd..

[313] Liu Z., Bai H., Sun D.D. (2012). Hierarchical CuO/ZnO membranes for environmental applications under the irradiation of visible light. Int. J. Photoenergy.

[314] Sapkota K.P., Lee I., Hanif M.A., Islam M.A., Hahn J.R. (2019). Solar-light-driven efficient ZnO–single-walled carbon nanotube photocatalyst for the degradation of a persistent water pollutant organic dye. Catalysts.

[315] Castilho C.J., Li D., Liu M., Liu Y., Gao H., Hurt R.H. (2019). Mosquito bite prevention through graphene barrier layers. Proc. Natl. Acad. Sci. USA.

[316] Kenry K., Lim Y.B., Nai M.H., Cao J., Loh K.P., Lim C.T. (2017). Graphene oxide inhibits malaria parasite invasion and delays parasitic growth in vitro. Nanoscale.

[317] Paul B., Panigrahi A.K., Singh V., Singh S.G. (2017). A multi-walled carbon nanotube–zinc oxide nanofiber based flexible chemiresistive biosensor for malaria biomarker detection. Analyst.

[318] Howard R.J., Uni S., Aikawa M., Aley S.B., Leech J.H., Lew A.M., Wellems T.E., Rener J., Taylor D.W. (1986). Secretion of a malarial histidine-rich protein (Pf HRP II) from Plasmodium falciparum-infected erythrocytes. J. Cell Biol..

[319] Zhao C., Tan S.X., Xiao X., Qiu X.S., Pan J.Q., Tang Z.X. (2014). Effects of dietary zinc oxide nanoparticles on growth performance and antioxidative status in broilers. Biol. Trace Elem. Res..

[320] Wadhwa R., Aggarwal T., Thapliyal N., Kumar A., Yadav P., Kumari V., Reddy B.S., Chandra P., Maurya P.K. (2019). Red blood cells as an efficient in vitro model for evaluating the efficacy of metallic nanoparticles. 3 Biotech.

[321] (2013). ASTM E2524-08 (2013): Standard Test Method for Analysis of Hemolytic Properties of Nanoparticles.

[322] (2017). ASTM F756: Standard Practice for Assessment of Hemolytic Properties of Materials.

[323] Babu E.P., Subastri A., Suyavaran A., Premkumar K., Sujatha V., Aristatile B., Alshammari G.M., Dharuman V., Thirunavukkarasu C. (2017). Size dependent uptake and hemolytic effect of zinc oxide nanoparticles on erythrocytes and biomedical potential of ZnO-ferulic acid conjugates. Sci. Rep..

[324] Kumar N., Pruthi V. (2014). Potential applications of ferulic acid from natural sources. Biotechnol. Rep..

[325] Khan M., Naqvi A.H., Ahmad M. (2015). Comparative study of the cytotoxic and genotoxic potentials of zinc oxide and titanium dioxide nanoparticles. Toxicol. Rep..

[326] Mahanta S., Prathap S., Ban D.K., Paul S. (2017). Protein functionalization of ZnO nanostructure exhibits selective and enhanced toxicity to breast cancer cells through oxidative stress-based cell death mechanism. J. Photochem. Photobiol. B.

[327] Bian Y., Kim K., Ngo T., Kim I., Bae O.N., Lim K.M., Chung J.H. (2019). Silver nanoparticles promote procoagulant activity of red blood cells: A potential risk of thrombosis in susceptible population. Part. Fibre Toxicol..

[328] Mahalakshmi S., Hema N., Vijaya P.P. (2020). In vitro biocompatibility and antimicrobial activities of zinc oxide nanoparticles (ZnO NPs) prepared by chemical and green synthetic route—A comparative study. Bionanoscience.

[329] Jan H., Shah M., Usman H., Khan M.A., Zia M., Hano C., Abbasi B.H. (2020). Biogenic synthesis and characterization of antimicrobial and antiparasitic zinc oxide (ZnO) nanoparticles using aqueous extracts of the Himalayan Columbine (Aquilegia pubiflora). Front. Mater..

[330] Rajapriya M., Sharmili S.A., Baskar R., Balaji R., Alharbi N.S., Kadaikunnan S., Khaled J.M., Alanzi K.F., Vaseeharan B. (2020). Correction to: Synthesis and characterization of zinc oxide nanoparticles using Cynara scolymus leaves: Enhanced hemolytic, antimicrobial, antiproliferative, and photocatalytic activity. J. Clust. Sci..

[331] Vinotha V., Iswarya A., Thaya R., Govindarajan M., Alharbi N.S., Kadaikunnan S., Khaled J.M., Al-Anbr M.N., Vaseeharan B. (2019). Synthesis of ZnO nanoparticles using insulin-rich leaf extract: Anti-diabetic, antibiofilm and anti-oxidant properties. J. Photochem. Photobiol. B.

[332] Rauf M.A., Oves M., Rehman F.U., Khan A.R., Husain N. (2019). Bougainvillea flower extract mediated zinc oxide’s nanomaterials for antimicrobial and anticancer activity. Biomed. Pharmacother..

[333] Hirayama D., Iida T., Nakase H. (2018). The phagocytic function of macrophage-enforcing innate immunity and tissue homeostasis. Int. J. Mol. Sci..

[334] Chang H., Ho C.C., Yang C.S., Chang W.H., Tsai M.H., Tsai H.T., Lin P. (2013). Involvement of MyD88 in zinc oxide nanoparticle-induced lung inflammation. Exp. Toxicol. Pathol..

[335] Alghsham R.S., Satpathy S.R., Bodduluri S.R., Hegde B., Jala V.R., Twal W., Burlison J.A., Sunkara M., Haribabu B. (2019). Zinc oxide nanowires exposure induces a distinct inflammatory response via CCL11-mediated eosinophil recruitment. Front. Immunol..

[336] Shen C., James S.A., de Jonge M.D., Turney T.W., Wright P.F., Feltis B.N. (2013). Relating cytotoxicity, zinc ions, and reactive oxygen in ZnO nanoparticle–Exposed human immune cells. Toxicol. Sci..

[337] Song W., Zhang J., Guo J., Zhang J., Ding F., Li L., Sun Z. (2010). Role of the dissolved zinc ion and reactive oxygen species in cytotoxicity of ZnO nanoparticles. Toxicol. Lett..

[338] Johnson B.M., Fraietta J.A., Gracias D.T., Hope J.L., Stairiker C.J., Patel P.R., Mueller Y.M., McHugh M.D., Jablonowski L.J., Wheatley M.A. (2015). Acute exposure to ZnO nanoparticles induces autophagic immune cell death. Nanotoxicology.

[339] Roy R., Singh S.K., Chauhan L.K., Das M., Tripathi A., Dwivedi P.D. (2014). Zinc oxide nanoparticles induce apoptosis by enhancement of autophagy via PI3K/Akt/mTOR inhibition. Toxicol. Lett..

[340] Yan G., Guo Y., Guo J., Wang W., Wang C., Wang X. (2020). N-Acetylcysteine attenuates lipopolysaccharide-Induced osteolysis by restoring bone remodeling balance via reduction of reactive oxygen species formation during osteoclastogenesis. Inflammation.

[341] Luo M., Shen C., Feltis B.N., Martin L.L., Hughes A.E., Wright P.F., Turney T.W. (2014). Reducing ZnO nanoparticle cytotoxicity by surface modification. Nanoscale.

[342] Nagajyothi P.C., Cha S.J., Yang I.J., Sreekanth T.V., Kim K.J., Shin H.M. (2015). Antioxidant and anti-inflammatory activities of zinc oxide nanoparticles synthesized using Polygala tenuifolia root extract. J. Photochem. Photobiol. B Biol..

[343] Thatoi P., Kerry R.G., Gouda S., Das G., Pramanik K., Thatoi H., Patra J.K. (2016). Photo-mediated green synthesis of silver and zinc oxide nanoparticles using aqueous extracts of two mangrove plant species, Heritiera fomes and Sonneratia apetala and investigation of their biomedical applications. J. Photochem. Photobiol. B Biol..

[344] Liu H., Kang P., Liu Y., An Y., Hu Y., Jin X., Cao X., Qi Y., Ramesh T., Wang X. (2020). Zinc oxide nanoparticles synthesised from the Vernonia amygdalina shows the anti-inflammatory and antinociceptive activities in the mice model. Artif. Cells Nanomed. Biotechnol..

